# The effects of traditional Chinese mind-body training on physical health in university students: a multilevel meta-analysis

**DOI:** 10.3389/fphys.2026.1792981

**Published:** 2026-04-10

**Authors:** Yaochi Cui, Jinxuan Liu, Xuwei Jin

**Affiliations:** 1 Institute of Physical Education and Training, Capital University of Physical Education and Sports, Beijing, China; 2 College of Education, Beijing Sport University, Beijing, China

**Keywords:** multilevel meta-analysis, physical health, taichi, traditional Chinese mind-body training, university students

## Abstract

**Objective:**

To evaluate the effects of traditional Chinese mind-body practices on university students’ physical health and examine whether training characteristics such as duration and frequency influence these outcomes.

**Methods:**

This systematic review and meta-analysis followed PRISMA guidelines. Randomized controlled trials (RCTs) were identified from PubMed, Web of Science, Embase, the Cochrane Library, and China National Knowledge Infrastructure (CNKI) based on PICOS criteria. Effect sizes were reported as mean difference (MD) or standardized mean difference (SMD). Data were synthesized using multilevel random-effects models. Heterogeneity was assessed using the I² statistic and Cochran’s Q test. Subgroup analyses and two-level random-effects meta-regression were conducted to explore potential moderators, including intervention type, frequency, session duration, intervention cycle, and total training dose. Sensitivity analyses and publication bias assessments were performed, and the certainty of evidence was evaluated using the GRADE framework.

**Results:**

Eighteen RCTs were included. Traditional Chinese mind-body practices were associated with improvements in several physical health indicators, including vital capacity (SMD = 0.34, 95% CI: 0.11 to 0.56, P = 0.003, GRADE: Moderate), BMI (MD = −0.77, 95% CI: −1.48 to −0.06, P = 0.034, GRADE: Moderate), resting heart rate (MD = −1.16, 95% CI: −2.29 to −0.04, P = 0.043, GRADE: Moderate), sit-and-reach test (MD = 2.99, 95% CI: 1.59 to 4.38, P = 0.000, GRADE: Moderate), and pull-up/sit-up performance (SMD = 0.40, 95% CI: 0.12 to 0.67, P = 0.005, GRADE: Low). The three-level model suggested improvement in the standing long jump, but this was not robust in the two-level sensitivity analysis. No significant changes were observed in handgrip strength, step test index, 50-m Sprint, or single-leg stance test (SLST). Subgroup analyses indicated that Baduanjin combined with Yijinjing, session durations of 45–60 minutes, training three to seven times per week, and intervention periods of 12–16 weeks were associated with larger improvements, although these findings were exploratory.

**Conclusion:**

Traditional Chinese mind-body training may improve several physical health indicators among university students, though effects varied across outcomes and interventions. Further large, well-designed RCTs are needed to confirm the consistency and long-term significance of these findings.

**Systematic Review Registration:**

https://www.crd.york.ac.uk/PROSPERO/view/CRD420251266434, identifier CRD420251266434.

## 1 Introduction

University students are at a critical stage of their academic and career development and often face multiple challenges, including academic stress, employment pressure, and irregular lifestyles. Consequently, many struggle with low levels of physical activity, declining fitness, and a lack of awareness about health management, which collectively contribute to various health risks (Morrell et al., 2013; Kang et al., 2014). Health status not only influences academic performance and quality of life but also plays a crucial role in the long-term development and health literacy of the youth population nationwide. Research shows that over 25% of adults globally do not meet the World Health Organization’s minimum physical activity guidelines (Guthold et al., 2018). This trend is particularly pronounced among university students. In the U.S., less than half of university students participate in at least 30 minutes of moderate exercise several times a week (Li et al., 2015), while adolescents in various countries are showing declines in endurance, strength, and flexibility (Kaster et al., 2020; Tomkinson et al., 2021). These observations underscore the pressing need to improve students’ physical health.

Physical activity is strongly associated with overall health. Activities ranging from moderate to vigorous intensity have been shown to help prevent obesity while improving both muscular strength and aerobic fitness (Clavero-Jimeno et al., 2024). Conversely, a lack of movement and inadequate sleep can negatively affect physical fitness (Fonseca et al., 2021). Recently, the benefits of traditional Chinese mind-body practices have gained significant recognition for their role in promoting health. These practices combine mental concentration, controlled breathing, and moderate physical activity, focusing on muscle stretching, relaxation, coordination, and the harmonious integration of breath with movement (Wu et al., 2019; Qi et al., 2022). Practices such as Tai Chi, Qigong, Baduanjin, Wuqinxi, Liuzijue, and Yijinjing (Dong and Bergren, 2016; Fan et al., 2020; Qi et al., 2022) are known for their gentle movements, flexible timing, and minimal space requirements. Despite differences in style, these practices share core features, including slow, stretching-based movements and coordinated breathing and mental focus, which justifies combining them in a meta-analysis. They tend to be more appealing and sustainable for university students than high-intensity workouts or extended aerobic sessions. Research has shown that mindfulness-based practices such as Baduanjin enhance health through synchronized breathing and movement (Dong and Bergren, 2016), while meta-analyses have indicated that Qigong significantly improves flexibility and cardiorespiratory fitness in university students (Lin et al., 2022). These results offer initial empirical support for the use of traditional Chinese mind-body practices to promote health among this demographic.

Previous research has largely focused on individual training methods, often constrained by limited participant numbers and inconsistent intervention protocols (Qi et al., 2022; Wang et al., 2025). There has been a notable lack of comprehensive studies examining the varying impacts of different training approaches and the ideal combinations of intervention parameters, such as duration and frequency. This gap has hindered the generation of robust evidence to inform practice. Consequently, this meta-analysis systematically gathered and analyzed randomized controlled trials to assess the overall impact of traditional Chinese mind-body training on various aspects of physical health among university students, including cardiorespiratory fitness, body composition, and overall physical fitness. Furthermore, the study investigated the potential moderating effects of training methods and intervention protocols to provide precise, evidence-based recommendations for developing health intervention programs tailored for university students, thereby laying a strong theoretical and empirical foundation for the promotion and application of traditional Chinese mind-body training.

## 2 Materials and methods

### 2.1 Study design

The research involved a comprehensive analysis of RCTs that adhered to the Preferred Reporting Items for Systematic Reviews and Meta-Analyses (PRISMA) guidelines (Moher et al., 2015). Before evaluating the search results, the study’s protocol was registered in advance with the International Prospective Register of Systematic Reviews (PROSPERO; ID: CRD420251266434) and was conducted in accordance with the PRISMA standards.

### 2.2 Study inclusion criteria

The inclusion criteria were as follows: RCTs that examined the impact of traditional Chinese mind-body practices on physical health outcomes among university students. To qualify, studies had to meet all of these conditions: (1) participants were university students, irrespective of their health conditions; (2) the intervention involved traditional Chinese mind-body practices, such as Tai Chi, Qigong, Baduanjin, Wuqinxi, Liuzijue, and similar activities. (3) the control group received no intervention (blank control), standard care, or alternative exercise programs, including aerobic workouts or stretching; (4) outcomes measured included physical health indicators such as fitness levels, lung capacity, and heart rate; and (5) the studies were published as randomized controlled trials in either English or Chinese.

The criteria for exclusion included studies that were not experimental in nature, such as theoretical discussions or case studies, as well as non-clinical research, including animal or cellular investigations. Additionally, secondary research, including systematic reviews and meta-analyses, was excluded, along with non-original works, such as duplicate articles or conference summaries. Gray literature, consisting of non-peer-reviewed documents, was also disregarded. Studies that did not focus on traditional Chinese mind-body practices, integrated these practices with other methods, lacked full-text access, or provided insufficient data for evaluation were excluded. Although systematic reviews were not included in the final selection, their reference lists were examined for potential qualifying studies.

### 2.3 Search strategy

A comprehensive literature search was conducted through databases such as PubMed, Web of Science, Embase, the Cochrane Library, and CNKI through November 20, 2025. The aim was to identify randomized controlled trials examining the effects of traditional Chinese mind–body practices on physical health in university students. The search methodology was structured around the PICOS criteria, with university students as the target population (P), traditional Chinese mind-body practices as the intervention (I), no intervention or alternative exercise methods as the comparator (C) physical health outcomes including cardiorespiratory fitness, body composition, muscular strength and endurance, flexibility, speed, and balance (e.g., vital capacity, BMI, RHR, standing long jump, handgrip strength, pull-ups/sit-ups, 50-m Sprint, step test index, sit-and-reach test, and SLST) as the outcomes (O), and randomized controlled trials (RCTs) as the research design (S).

A set of keywords and subject phrases was used, including: (“Traditional Chinese mind-body practice” OR “mind-body practice” OR “Traditional Chinese workout” OR “Tai Chi” OR “Qigong” OR “Wu Qin Xi” OR “Baduanjin” OR “Yijinjing” OR “Liuzijue”) AND (“physical wellness” OR “health” OR “wellness” OR “physical fitness”) AND (“college learners” OR “undergraduate students” OR “university attendees”). The complete search methodology is provided in the **Supplementary File**.

### 2.4 Study selection process

The selection of studies closely followed the PRISMA framework. All identified records were uploaded to Zotero version 7.0 to eliminate duplicates. Two reviewers independently assessed titles and abstracts to exclude irrelevant studies. The level of agreement between reviewers was measured using Cohen’s κ statistic, with outcomes categorized as inclusion (1) or exclusion (0) (Cohen, 1960). Full texts of studies that appeared to meet eligibility criteria were obtained and assessed using the PICOS framework. Any differences in judgment were resolved through discussions involving a third reviewer. Data extraction was conducted independently by two reviewers using standardized forms, and essential variables, such as participant demographics and intervention details, were cross-verified. Any inconsistencies were resolved through consensus among the research team.

### 2.5 Data synthesis

Meta-analyses were conducted in R (version 4.3.3) using the meta, metafor, and ggplot2 packages. For continuous outcomes, either MD or SMD was chosen as the effect size, based on the uniformity of measurement tools across the studies. SMDs were calculated using Hedges’ g, with effect sizes classified as small (g = 0.3), moderate (g = 0.5), and large (≥ 0.8) (Nakagawa and Cuthill, 2007). The main analyses were conducted using multilevel (three-level) random-effects models via the *rma.mv* function in the metafor package. Because several studies reported multiple effect sizes, the data exhibited a hierarchical structure with effect sizes nested within studies. The three-level model accounts for sampling variance, within-study variance, and between-study variance, thereby addressing the potential dependency among effect sizes (Assink and Wibbelink, 2016).

The evaluation of statistical heterogeneity was performed using Cochran’s Q test (P < 0.10 indicating significance) and the I² statistic; values exceeding 50% suggest considerable heterogeneity (Lau et al., 1997). Publication bias was examined using funnel plots and Egger’s regression test, with adjustments made using the trim-and-fill approach (Hong et al., 2020). Influential outliers were identified by analyzing standardized residuals (|Z| > 2.5) and Cook’s distance (exceeding 3× the average) (Viechtbauer and Cheung, 2010). Sensitivity analyses included: (1) leave-one-out assessments using the metainf function, (2) subgroup analyses, and (3) conventional two-level random-effects models were additionally conducted for comparison and to examine the robustness of the pooled estimates. To further explore potential sources of heterogeneity for outcomes with high I² values, meta-regression analyses were conducted, including intervention mode, frequency, intervention cycle, session duration, and total training dose. To harmonize outcomes measured on different scales, raw data were transformed using the Hedges–Olkin method as follows:


$$\text{SMD}=\frac{M_{\text{Intervention}}-M_{\text{Control}}}{{\text{SD}}_{\text{Pooled}}},\;{\text{SD}}_{\text{Pooled}}=\sqrt{\frac{(n_{1}-1){\text{SD}}_{1}^{2}+(n_{2}-1){\text{SD}}_{2}^{2}}{n_{1}+n_{2}-2}}$$

(Hedges and Olkin, 2014).

### 2.6 Risk of bias (quality) assessment

The evaluation of bias in the randomized controlled trials included in the study was conducted using the updated Cochrane Risk of Bias tool (RoB 2.0, 2019 edition), which examined the following areas: (1) bias from the randomization process, (2) bias from deviations from intended interventions, (3) bias from missing outcome data, (4) bias in measuring outcomes, and (5) bias in the selection of reported results (Sterne et al., 2019). This evaluation was conducted in a blinded, independent manner. Two reviewers assessed the methodological quality of each trial, categorizing trials as low risk, some concerns, or high risk of bias. Any disagreements were resolved through discussion, and unresolved matters were referred to a third reviewer. The overall confidence in the evidence for primary outcomes (indicators of physical health) and all subgroup analyses was assessed using the GRADE framework, following specific criteria for downgrading. Findings were summarized in tables created with the GRADEpro GDT online tool (Guyatt et al., 2008).

## 3 Results

### 3.1 Study selection

**Figure 1:**
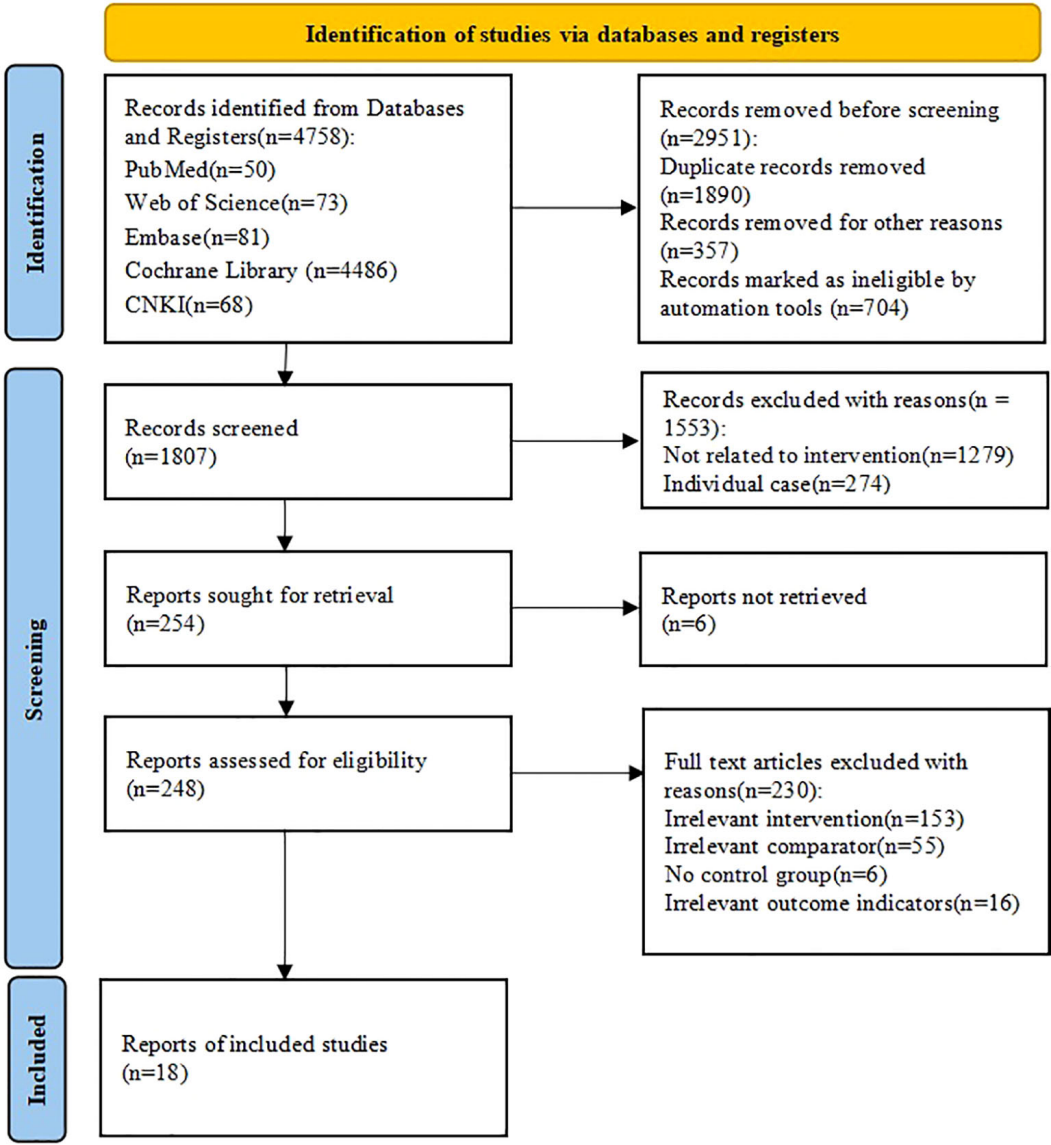
Flow diagram of the selection process.

A comprehensive search across five databases identified 4,758 records (**Figure 1**). After removing duplicate records (n = 1,890), excluding records for other reasons (n = 357), and filtering out records using automated screening tools (n = 704), 1,807 records remained for title and abstract screening. At this stage, inter-rater agreement was substantial (Cohen’s κ = 0.82). Following screening, 1,553 records were excluded, leaving 254 reports for full-text retrieval. Of these, 6 reports were not retrieved, and 248 full-text articles were assessed for eligibility. Inter-rater agreement during full-text evaluation was also high (Cohen’s κ = 0.83) (see **Supplementary File**). After full-text assessment, 230 articles were excluded due to irrelevant intervention (n = 153), irrelevant comparator (n = 55), no control group (n = 6), or irrelevant outcome indicators (n = 16). Ultimately, 18 RCTs met the inclusion criteria and were included in the meta-analysis.

### 3.2 Risk of bias of included studies

**Figure 2:**
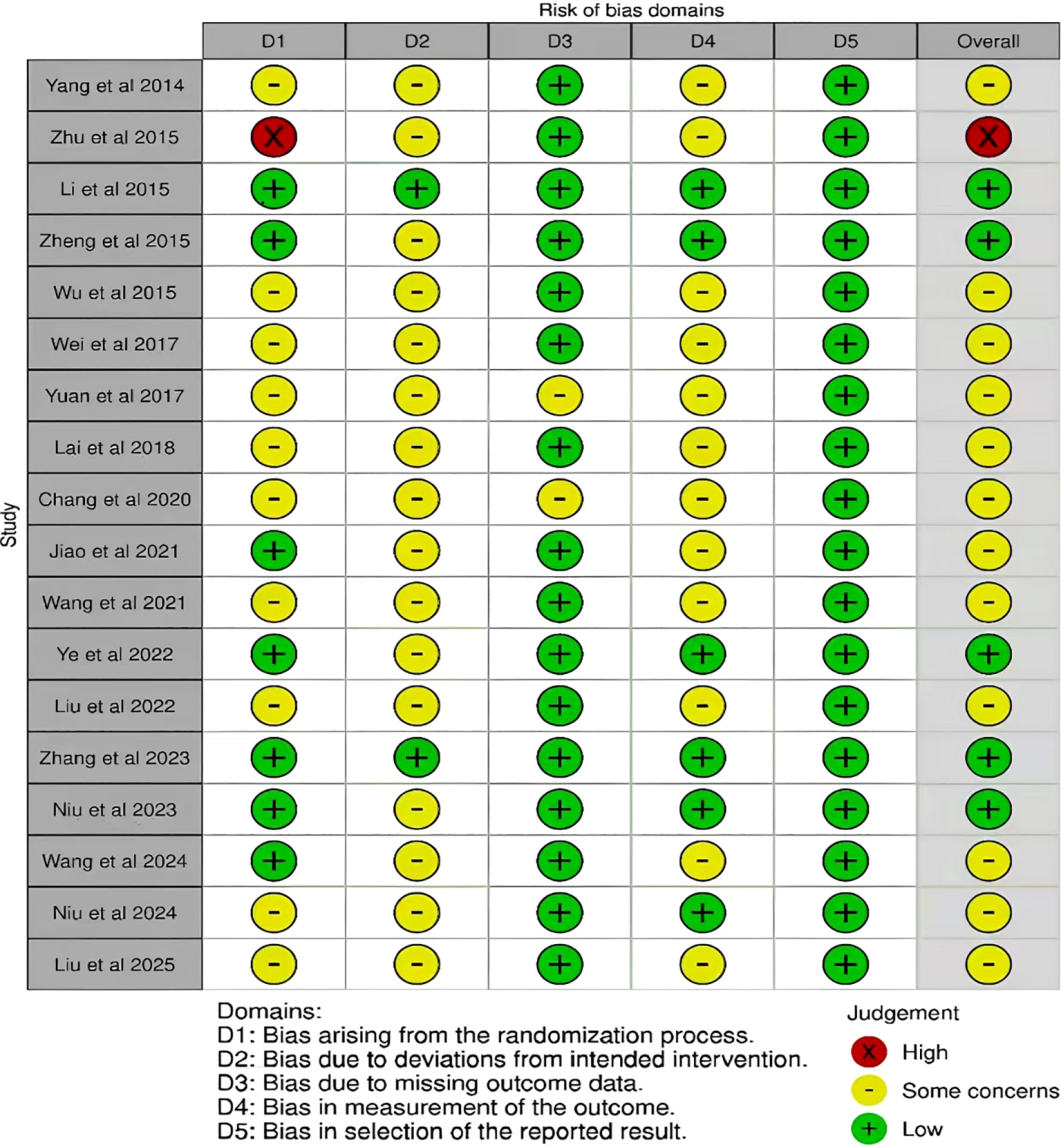
Risk of bias summary.

**Figure 3:**
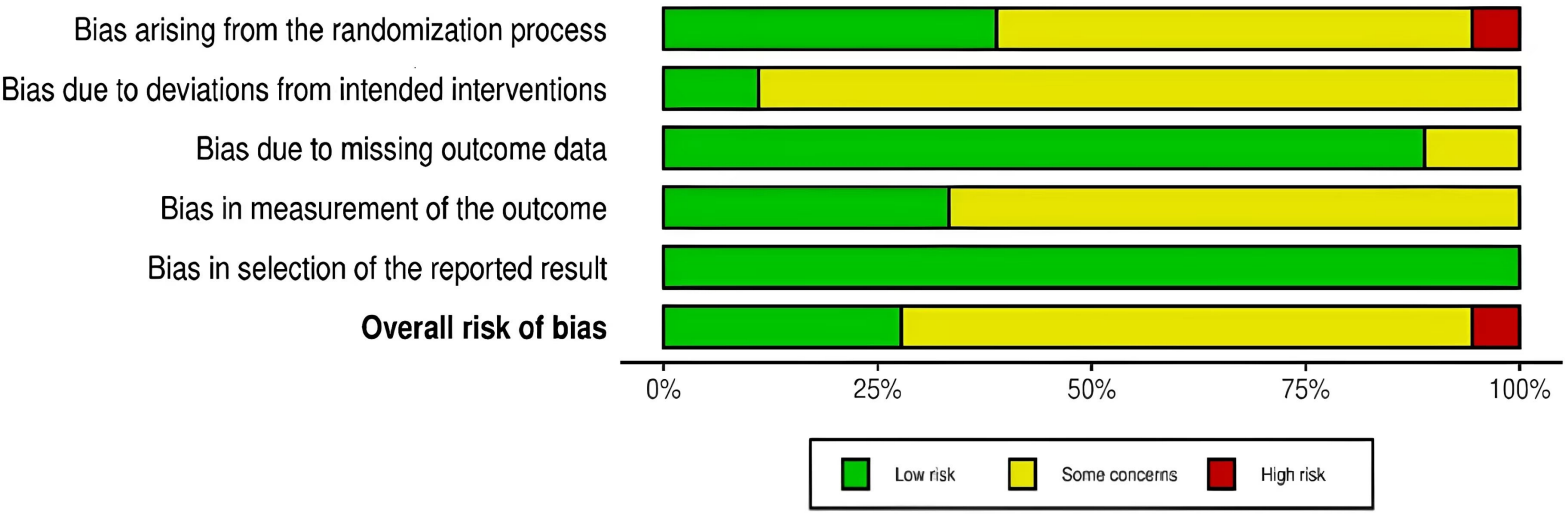
Risk of bias graph for included studies.

**Figures 2** and **3** present the risk-of-bias evaluations for the 18 RCTs included in the analysis, with a red “×” denoting high risk, a yellow “–” indicating some concerns, and a green “+” representing low risk. Each study was independently assessed by two reviewers using the Cochrane Risk of Bias tool (RoB 2.0) across five domains: bias from the randomization process (D1), bias from deviations in intended interventions (D2), bias from missing outcome data (D3), bias in measuring outcomes (D4), and bias in the selection of reported results (D5). Any disagreements were resolved through discussion or by involving a third reviewer.

The majority of studies effectively outlined the processes of random sequence generation and allocation concealment; however, one study was classified as high risk due to inadequate methodological details (Zhu, 2015). Given the nature of the interventions, it was challenging to achieve blinding for both participants and personnel. Regarding data completeness, multiple studies documented attrition and used intention-to-treat analyses (Li et al., 2015; Zheng et al., 2015; Ye et al., 2022; Niu et al., 2023; Zhang and Jiang, 2023; Niu et al., 2024), while others raised certain issues. The inter-rater agreement across various domains was as follows: for D1 (randomization process), the simple agreement rate was 72.2% (13/18), with Cohen’s κ at 0.50 and weighted κ at 0.63, indicating a “Moderate” level; for D2 (deviations from intended interventions), the agreement rate was 55.6% (10/18), with Cohen’s κ at 0.11 and weighted κ at 0.28, categorized as “Fair”; for D3 (missing outcome data), the agreement rate was 100.0% (18/18), with Cohen’s κ at 1.00 and weighted κ at 1.00, deemed “Almost Perfect”; for D4 (measurement of outcomes), the agreement rate was 66.7% (12/18), with Cohen’s κ at 0.36 and weighted κ at 0.54, interpreted as “Moderate”; for D5 (selection of the reported result), the agreement rate was 88.9% (16/18), with Cohen’s κ at 0.77 and weighted κ at 0.87, classified as “Very Good.”

In general, the majority of the studies assessed were considered to have a low likelihood of D3 and D5, suggesting that follow-up data were largely complete and outcome reporting was transparent. However, the areas concerning D1, D2, and D4 showed a mix of low risk and some issues, with one study classified as high risk in both D1 and the overall bias assessment.

The results indicated that several methodological shortcomings persisted across studies, especially regarding the generation of random sequences, allocation concealment, blinding, adherence management, and outcome measurement or assessor blinding. As a result, any potential systematic bias linked to D1 and D2 was approached with caution in both primary and subgroup analyses and was carefully taken into account during sensitivity analyses and evaluations of evidence certainty (e.g., GRADE).

### 3.3 Study characteristics

Eighteen RCTs were analyzed, involving 1,724 individuals, and published between 2014 and 2025 in China and Thailand (see **Table 1**). The number of participants in each trial ranged from 28 to 222. Of the total participants, approximately 44.2% identified as male and 55.8% as female, although one trial did not disclose a gender breakdown. The majority of participants were general university students, with certain groups including smokers and those not majoring in physical education.

**Table 1:** Characteristics of the studies in the multilevel meta-analysis.

Authors	Country	Participant type	Intervention frequency (times/week)	Intervention duration (weeks)	Exercise time (min/session)	Total sample	Intervention mode	Scale type
(Yang, 2014)	China	University students	5	15	90	132	Tai Chi (High frequency vs. Low frequency)	Vital capacity, Step Test, Sit-and-reach test, Step test index
(Zhu, 2015)	China	University students	3	104	45	100	Tai Chi vs. Non-exercise	Vital capacity, Handgrip strength, Step test index
(Li et al., 2015)	China	University students	5	12	60	222	Baduanjin vs. Regular activities	Handgrip strength, Vital capacity, Sit-and-reach test, Standing long jump, Step test index, RHR
(Zheng et al., 2015)	China	University students	5	12	60	96	24-form Tai Chi vs. Daily activities	Vital capacity, RHR
(Wu and Hou, 2015)	China	University students	3	24	60	80	24-form Tai Chi vs. Daily activities	BMI, SLST, Step test index, RHR
(Wei et al., 2017)	China	University students	5	13	60	120	Yijinjing vs. Non-intervention	Handgrip strength, Vital capacity, Sit-and-reach test, Standing long jump, Step test index
(Yuan et al., 2017)	China	University students	4	20	60	28	Tai Chi vs. Non-intervention	BMI, Vital capacity, RHR
(Lai et al., 2018)	China	Female university students	5	13	60	60	Baduanjin vs. Non-intervention	Vital capacity, Sit-and-reach test, Standing long jump
(Chang and Zhang, 2020)	China	Female university students of non-sports majors	3	16	60	65	The 18 Essential Forms of Chen-style Tai Chi vs. Non-intervention	Vital capacity, RHR
(Jiao et al., 2021)	China	Female university students	5	16	80	80	Wuqinxi vs. Other Exercises	BMI, Handgrip strength, Vital capacity
(Wang, 2021)	China	Female university students of non-sports majors	3-4	17	60-90	60	Tai Chi vs. Aerobic	BMI, Vital capacity, SLST, Step test index, RHR
(Ye et al., 2022)	China	Healthy university students	4	12	60	130	Baduanjin vs. Non-intervention	BMI, Handgrip strength, Vital capacity, 50-Meter Sprint, Sit-and-reach test, Standing long jump, Sit-and-reach test, Pull-ups/sit-ups, Step test index
(Liu and Zhang, 2022)	China	University students	7	12	60	128	Baduanjin + Yijinjing vs. Non-intervention	BMI, Vital capacity, 50-Meter Sprint, Sit-and-reach test, Standing long jump, Sit-and-reach test, Pull-ups/sit-ups
(Zhang and Jiang, 2023)	China	Female university students	3	12	60	78	Baduanjin vs. Non-intervention	BMI
(Niu et al., 2023)	China	Overweight male university students	3	12	60	81	Eight-step and Five-Method Tai Chi vs. He-style Taijiquan vs. Non-intervention	BMI
(Wang et al., 2024)	China	Disadvantaged university students in sports	3	16	60	93	Baduanjin vs. Non-intervention	RHR
(Niu et al., 2024)	Thailand	Overweight male university students	3	12	60	81	Bafa Wubu Taijiquan vs. Tai Chi vs. Non-intervention	SLST
(Liu et al., 2025)	China	Female university students	5	10	45	90	Baduanjin vs. Brisk walking vs. Non-intervention	Vital capacity, RHR

times/week, Times per week; x/day, times per day; min/session, Minutes per session; BMI, Body Mass Index; SLST, Single-Leg Stance Test; RHR, resting heart rate.

All research used traditional Chinese mind-body practices as the primary intervention. The most commonly studied interventions were Tai Chi, including variations such as the 24-form Tai Chi, 18-form Chen-style Tai Chi, Babu Wufa, and He-style Tai Chi, as well as standalone practices such as Baduanjin, Yijinjing, and Wuqinxi. Interventions typically occurred three to five times a week, although a few studies included daily sessions. Intervention lengths ranged from 3 to 24 weeks, comprising 10 short-term (≤12 weeks), 7 medium-term (13–24 weeks), and 1 long-term (≥24 weeks) program. Each session lasted between 25 and 90 minutes, with 60 minutes the most common duration across 16 studies.

The control groups included several comparators: no intervention, usual or daily activities, and alternative exercise programs (e.g., aerobic exercise or brisk walking). These comparator conditions were recorded and categorized in the study characteristics table. Every study included in the review documented at least one physical health outcome. In detail, 13 studies focused on vital capacity (Yang, 2014; Li et al., 2015; Zheng et al., 2015; Zhu, 2015; Wei et al., 2017; Yuan et al., 2017; Lai et al., 2018; Chang and Zhang, 2020; Jiao et al., 2021; Wang, 2021; Liu and Zhang, 2022; Ye et al., 2022; Liu et al., 2025), 8 examined BMI (Wu and Hou, 2015; Yuan et al., 2017; Jiao et al., 2021; Wang, 2021; Liu and Zhang, 2022; Ye et al., 2022; Niu et al., 2023; Zhang and Jiang, 2023), another 8 assessed RHR (Li et al., 2015; Wu and Hou, 2015; Zheng et al., 2015; Yuan et al., 2017; Chang and Zhang, 2020; Wang, 2021; Wang et al., 2024; Liu et al., 2025), 7 looked into the step test index (Yang, 2014; Li et al., 2015; Wu and Hou, 2015; Zhu, 2015; Wei et al., 2017; Wang, 2021; Ye et al., 2022), 7 evaluated sit-and-reach test (Yang, 2014; Li et al., 2015; Wei et al., 2017; Lai et al., 2018; Liu and Zhang, 2022; Ye et al., 2022; Niu et al., 2024), 6 measured handgrip strength (Li et al., 2015; Zhu, 2015; Wei et al., 2017; Jiao et al., 2021; Ye et al., 2022; Niu et al., 2024), 5 investigated the standing long jump (Li et al., 2015; Wei et al., 2017; Lai et al., 2018; Liu and Zhang, 2022; Ye et al., 2022). In comparison, 2 studies each reported on the 50-meter Sprint (Liu and Zhang, 2022; Ye et al., 2022), pull-ups/sit-ups (Liu and Zhang, 2022; Ye et al., 2022), and SLST (Wu and Hou, 2015; Wang, 2021).

### 3.4 Multilevel meta-analysis results

#### 3.4.1 Vital capacity

**Figure 4:**
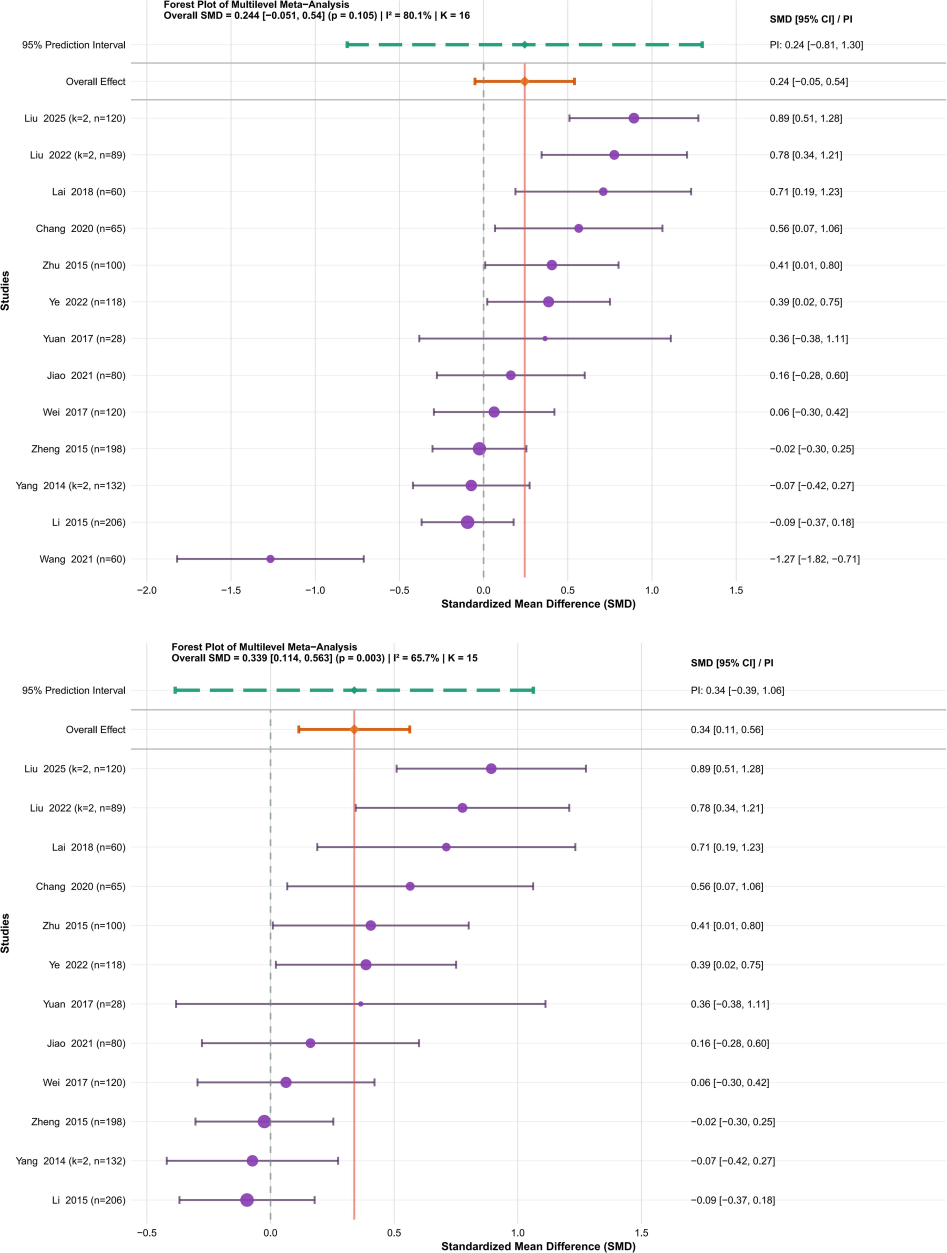
Forest plot showing the effect of traditional Chinese mind-body training on the vital capacity of university students. *Upper panel:* Initial analysis including 13 studies (SMD = 0.24, 95% CI: -0.05 to 0.54, P = 0.105); *Lower panel:* Updated analysis after excluding one outlier (Wang et al. (2021) (Wang, 2021)), including 12 studies (SMD = 0.34, 95% CI: 0.11 to 0.56, P = 0.003). For all forest plots, SMD, standardized mean difference; p, Statistical significance p-value; I^2^, Inconsistency index; K, Number of combined effect sizes; Statistical significance is assumed if the 95% Confidence Interval (95% CI) does not include zero; n, Sample size.

The analysis encompassed 13 studies on vital capacity (Yang, 2014; Li et al., 2015; Zheng et al., 2015; Zhu, 2015; Wei et al., 2017; Yuan et al., 2017; Lai et al., 2018; Chang and Zhang, 2020; Jiao et al., 2021; Wang, 2021; Liu and Zhang, 2022; Ye et al., 2022; Liu et al., 2025) involving 1,639 university students. Because vital capacity was reported using different units or indicator forms across studies (e.g., mL, L, or ratio-based indices such as vital capacity/BMI), SMD was calculated as the effect size. Heterogeneity analysis indicated substantial variability across studies (I² = 80.1%). In addition, some studies reported multiple effect sizes, resulting in a hierarchical data structure with effect sizes nested within studies. Variance partitioning indicated that between-study heterogeneity accounted for 51.3% of the total variability, with within-study heterogeneity accounting for 48.7% (see **Supplementary File**). The three-level random-effects meta-analysis indicated that traditional Chinese mind-body training did not lead to a statistically significant improvement in vital capacity among university students (SMD = 0.24, 95% CI: −0.05 to 0.54, P = 0.105) (**Figure 4**, upper panel).

Given the significant heterogeneity observed (I² > 50%), the possibility of publication bias in smaller studies was assessed using Egger’s regression test, in accordance with the guidelines outlined in the Cochrane Handbook (version 6.3) (Gillespie et al., 2012). The findings indicated a possible inclination toward publication bias (t = 1.5, P = 0.156) (see **Supplementary File**). Additional influence diagnostics using standardized residuals (|Z| > 2.5) and Cook’s distance criteria (exceeding three times the average) (Viechtbauer and Cheung, 2010) indicated that the research by Wang et al. (2021) (Wang, 2021) exceeded both thresholds; consequently, this study was excluded (see **Supplementary File**). Sensitivity analyses confirmed the stability of the results, with a pooled SMD ranging from 0.24 to 0.34 after excluding individual studies (**Figure 4**, lower panel). A trim-and-fill analysis suggested the possible presence of one missing study, but the results remained stable. (see **Supplementary File**).

In total, the updated review included 12 studies. The random-effects model revealed substantial heterogeneity (I² = 65.7%), and the combined findings indicated a statistically significant improvement in vital capacity with traditional Chinese mind-body practices (SMD = 0.34, 95% CI: 0.11 to 0.56, P = 0.003) (see **Figure 4**, lower section). A thorough evaluation using the GRADE framework provided moderate-quality evidence that traditional Chinese mind-body training notably improved vital capacity among university students (see **Supplementary File**). A two-level random-effects model was conducted as a sensitivity analysis, yielding results similar to those of the main analysis (SMD = 0.35, 95% CI: 0.11 to 0.60, P = 0.008), indicating consistent findings (see **Supplementary File**).

#### 3.4.2 BMI

**Figure 5:**
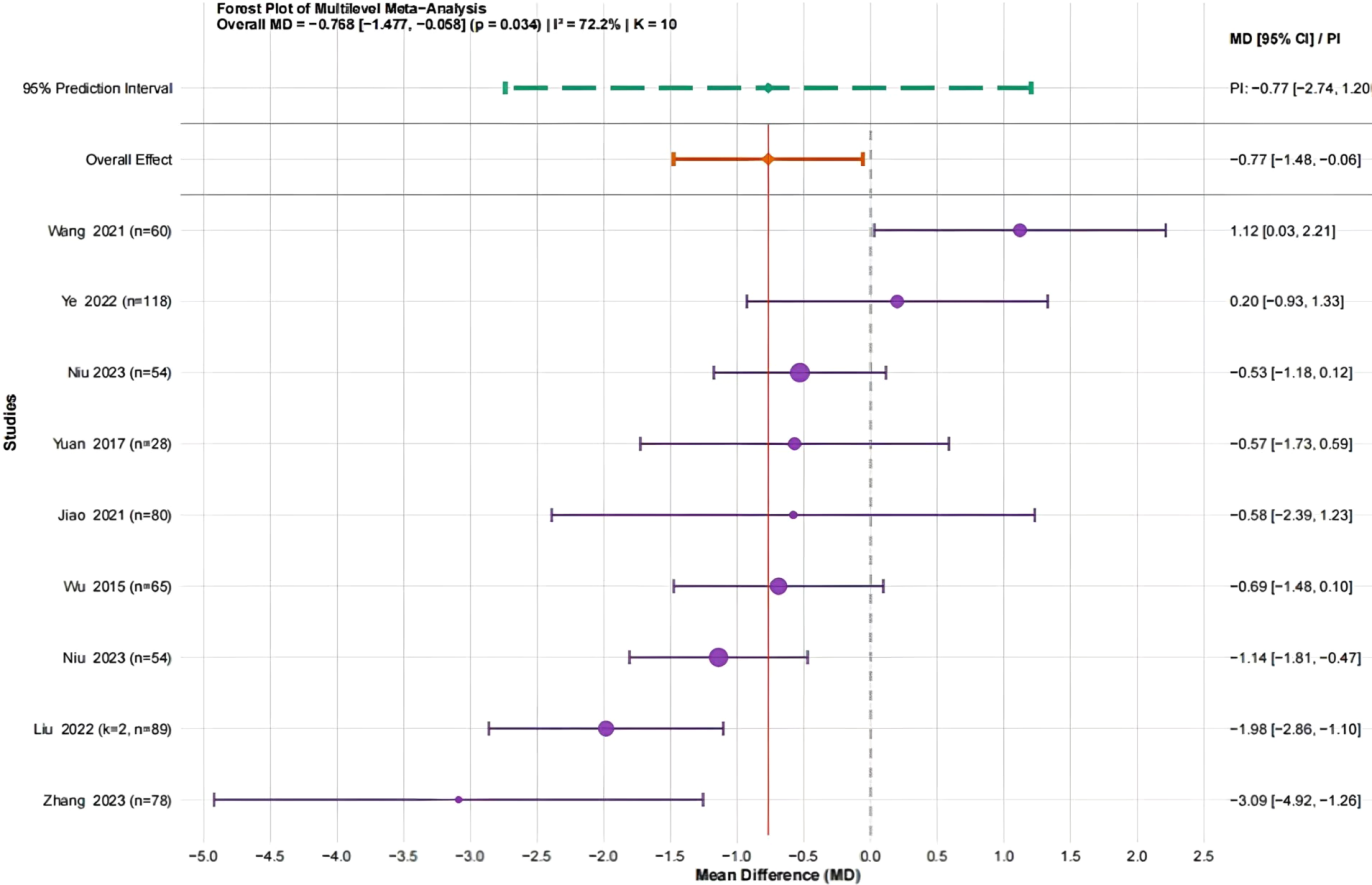
Forest plot showing the effect of traditional Chinese physical and mental training on the BMI of university students.

A total of eight studies (Wu and Hou, 2015; Yuan et al., 2017; Jiao et al., 2021; Wang, 2021; Liu and Zhang, 2022; Ye et al., 2022; Niu et al., 2023; Zhang and Jiang, 2023) involving 746 university students were analyzed. All studies used the same measurement tools, so MD was selected as the effect size. The heterogeneity analysis revealed substantial variability across studies (I² = 72.2%). Variance partitioning indicated that between-study heterogeneity accounted for 75.4% of the total variability, while within-study heterogeneity accounted for 24.6% (see **Supplementary File**). The three-level random-effects meta-analysis indicated that traditional Chinese mind-body training significantly improved BMI in university students (MD = −0.77, 95% CI: −1.48 to −0.06, P = 0.034) (**Figure 5**).

Given the substantial heterogeneity (I² = 72.2%), additional analyses were conducted to examine potential bias and influential studies. Egger’s regression test did not indicate significant publication bias (t = −0.607, P = 0.561); however, the small number of studies may limit the test’s power (see **Supplementary File**). Influence diagnostics using standardized residuals and Cook’s distance identified Wang et al. (2021) (Liu et al., 2025) as having a relatively large influence; however, it did not meet the exclusion criteria. Leave-one-out sensitivity analyses confirmed that the pooled estimates remained stable (see **Supplementary File**). Trim-and-fill analysis suggested the possible presence of one missing study, but the overall pattern of results remained unchanged (see **Supplementary File**).

In summary, traditional Chinese mind–body training significantly reduced BMI among university students (MD = −0.77, 95% CI: −1.48 to −0.06, P = 0.034). The GRADE evaluation rated the evidence as moderate quality, suggesting that while there is some evidence regarding the impact of this training on BMI, further research is needed to confirm the findings. (see **Supplementary File**). A conventional two-level random-effects model was additionally conducted as a sensitivity analysis and yielded a similar effect estimate (MD = −0.85, 95% CI: −1.70 to −0.01, P = 0.047) (see **Supplementary File**), indicating consistent findings.

#### 3.4.3 RHR

**Figure 6:**
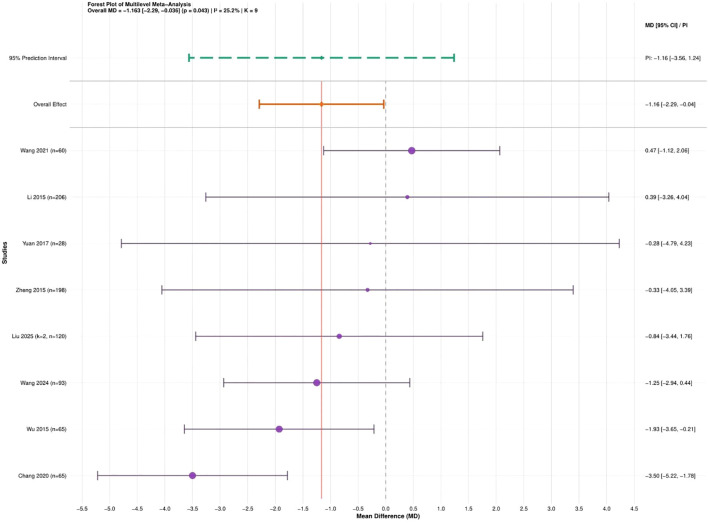
Forest plot of the effect of traditional Chinese mind-body training on RHR in university students.

A total of eight studies (Li et al., 2015; Wu and Hou, 2015; Zheng et al., 2015; Yuan et al., 2017; Chang and Zhang, 2020; Wang, 2021; Wang et al., 2024; Liu et al., 2025) involving 788 university students were analyzed. All studies reported resting heart rate using the same measurement unit; therefore, MD was selected as the effect size. The heterogeneity assessment revealed low variability among studies (I² = 25.2%). Variance partitioning indicated that between-study heterogeneity accounted for 100% of the total variability, while within-study heterogeneity was negligible (see **Supplementary File**). The three-level random-effects meta-analysis showed that traditional Chinese mind-body training significantly reduced RHR among university students compared with the control group (MD = −1.16, 95% CI: −2.29 to −0.04, P = 0.043) (**Figure 6**).

Sensitivity analyses were conducted to assess the robustness of the findings. Sequential exclusion of individual studies did not materially change the pooled estimates, indicating that the results were stable (see **Supplementary File**). Because fewer than ten studies were included (k = 8), Egger’s regression test was not performed.

To conclude, traditional Chinese mind-body training led to a significant decrease in resting heart rate among university students (MD = −1.16, 95% CI: −2.29 to −0.04, P = 0.043). The GRADE evaluation indicated that this finding is supported by moderate-quality evidence (see **Supplementary File**). A conventional two-level random-effects model was also conducted as a sensitivity analysis, yielding a similar effect estimate (MD = −1.16, 95% CI: −2.25 to −0.07, P = 0.039), indicating consistent findings.

#### 3.4.4 Step test index

A total of seven studies (Yang, 2014; Li et al., 2015; Wu and Hou, 2015; Zhu, 2015; Wei et al., 2017; Wang, 2021; Ye et al., 2022) involving 844 university students were analyzed. Because the same measurement units were used, MD was chosen as the effect size. The heterogeneity assessment revealed high variability (I² = 87.1%). The combined results indicated that traditional Chinese mind-body training did not significantly enhance the step test index compared with the control group (MD = 0.85, 95% CI: −2.30 to 4.00, P = 0.598) (see **Supplementary File**).

Given significant heterogeneity (I² > 50%), sensitivity analyses were conducted according to the predetermined protocol. Evaluations using standardized residuals and Cook’s distance indicated that all studies fell within acceptable limits, with no significant outliers detected (see **Supplementary File**). Gradual exclusion of individual studies produced consistent effect directions (MD between 0.04 and 0.32), yet all 95% confidence intervals encompassed zero, suggesting that the results were stable but the evidence was not robust.

In summary, a synthesis of data from seven studies found that conventional Chinese mind-body practices did not meaningfully enhance the step test index among university students (MD = 0.85, 95% CI: −2.30 to 4.00, P = 0.598). The GRADE framework assessed the reliability of this evidence as very low (see **Supplementary File**).

#### 3.4.5 Sit-and-reach test

**Figure 7:**
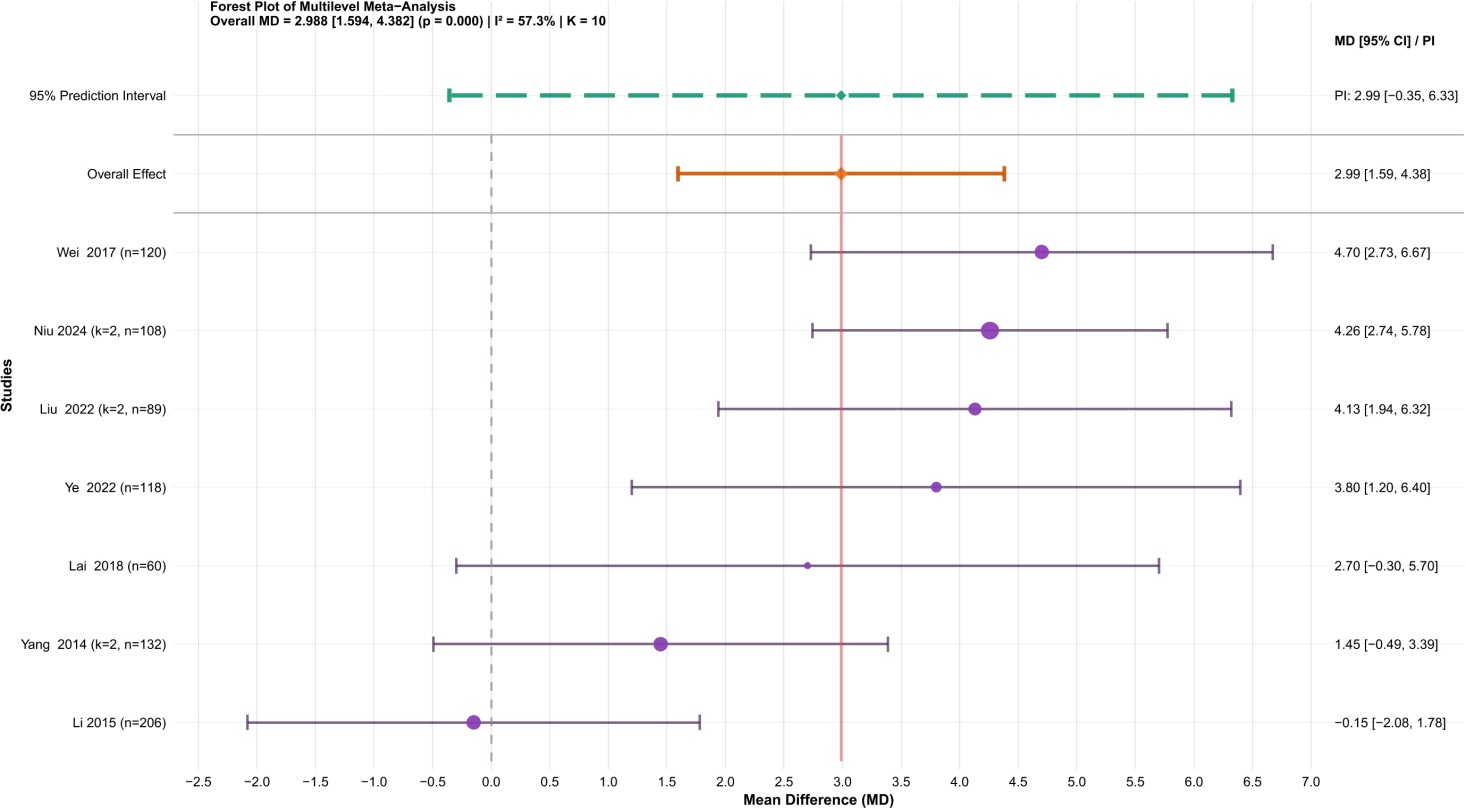
Forest plot of the effect of traditional Chinese mind-body training on the sit-and-reach test in university students.

A total of seven studies (Yang, 2014; Li et al., 2015; Wei et al., 2017; Lai et al., 2018; Liu and Zhang, 2022; Ye et al., 2022; Niu et al., 2024) involving 1082 university students were analyzed. Because the outcome was reported using the same measurement units, MD was selected as the effect size. The heterogeneity assessment revealed moderate variability among studies (I² = 57.3%). Variance partitioning indicated that between-study heterogeneity accounted for most of the total variability (90.3%), while within-study heterogeneity was negligible (9.7%) (see **Supplementary File**). The three-level random-effects meta-analysis showed that traditional Chinese mind-body training significantly enhanced the sit-and-reach test (MD = 2.99, 95% CI: 1.59 to 4.38, P = 0.000) (**Figure 7**).

Egger’s regression assessment revealed no notable publication bias (t = 0.301, P = 0.771). Influence diagnostics indicated that Li et al. (2015) (Li et al., 2015) exceeded the Cook’s distance limit, yet its standardized residual remained within acceptable bounds (see **Supplementary File**). Sensitivity analyses omitting a single study consistently yielded positive, statistically significant pooled effects (MDs between 0.49 and 0.64), demonstrating the results’ strong reliability. The trim-and-fill analysis suggested one potentially missing study, and the revised findings maintained both the effect direction and statistical significance, further reinforcing the conclusion’s stability (see **Supplementary File**).

In conclusion, traditional Chinese mind-body training significantly improved the sit-and-reach test in university students (MD = 2.99, 95% CI: 1.59 to 4.38, P = 0.000). The GRADE evaluation rated the evidence as moderate quality (see **Supplementary File**). A two-level random-effects model was also conducted, yielding results similar to those of the fixed-effects model (MD = 3.07, 95% CI: 1.66 to 4.47, P = 0.0008), confirming the robustness of the findings.

#### 3.4.6 Handgrip strength

A total of six studies (Li et al., 2015; Zhu, 2015; Wei et al., 2017; Jiao et al., 2021; Ye et al., 2022; Niu et al., 2024) involving 814 university students were analyzed. Due to variations in measurement scales (e.g., kg or kgf), SMD was chosen as the effect size. The heterogeneity assessment indicated high variability (I² = 84.9%). The random-effects combined analysis suggested that traditional Chinese mind-body training did not significantly enhance handgrip strength (SMD = 0.21, 95% CI: −0.41 to 0.83, P = 0.500) (see **Supplementary File**). Given the considerable heterogeneity and the small number of studies (k = 6, fewer than 10), Egger’s regression test was not conducted. Influence diagnostics indicated that Zhu et al. (2015) (Zhu, 2015) exceeded the Cook’s distance limit, but its standardized residual did not warrant exclusion (see **Supplementary File**). Leave-one-out analyses revealed consistent effect directions (SMD between −0.09 and 0.28), with all 95% confidence intervals encompassing zero, suggesting stable yet weak evidence (see **Supplementary File**).

In summary, conventional mind-body practices from Chinese traditions did not significantly enhance handgrip strength in university students (SMD = 0.21, 95% CI: −0.41 to 0.83, P = 0.500). According to the GRADE framework, the reliability of this evidence was assessed as very low (see **Supplementary File**).

#### 3.4.7 Standing long jump

**Figure 8:**
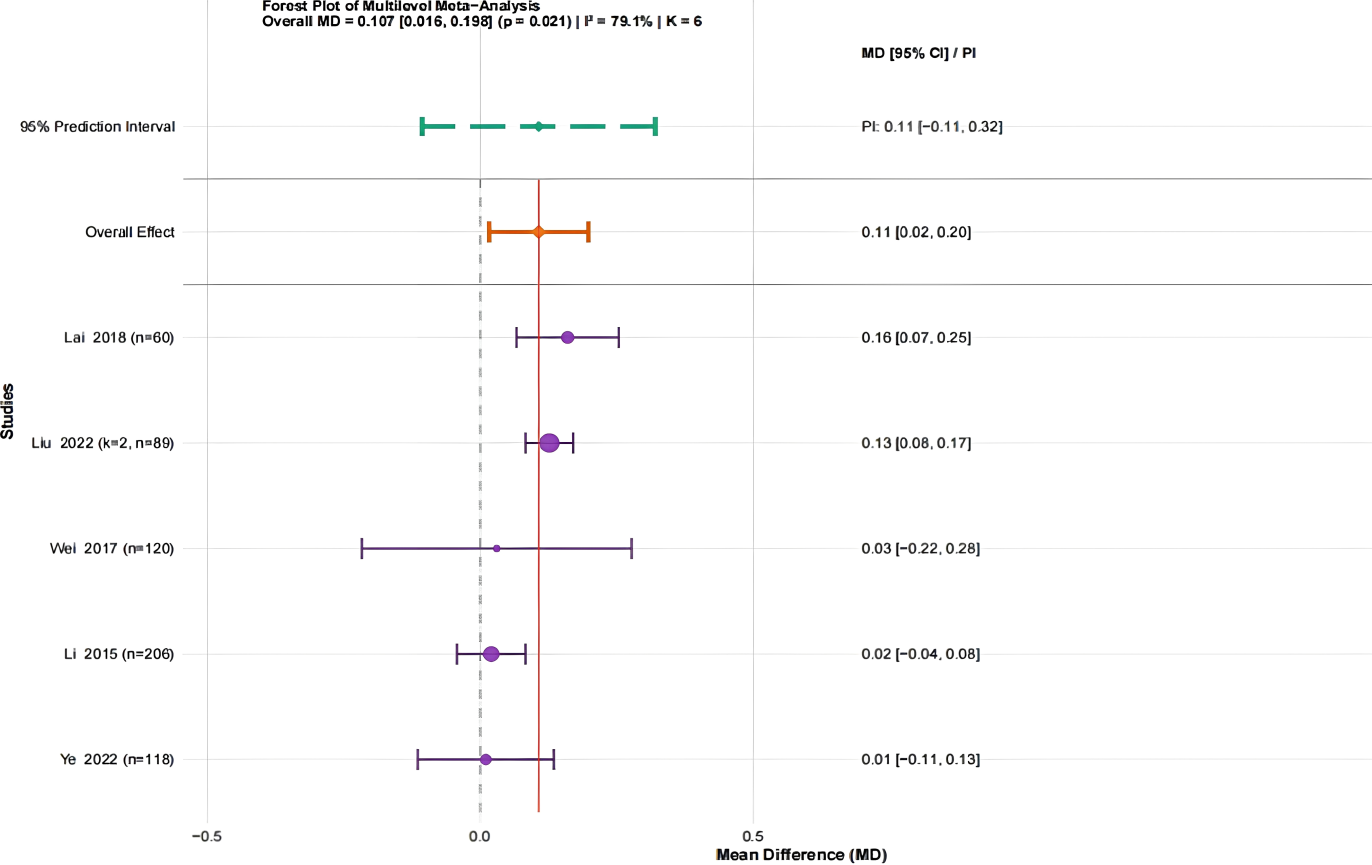
Forest plot of the effect of traditional Chinese mind-body training on the Standing Long Jump in university students. All values are expressed in meters (m).

A total of five studies (Li et al., 2015; Wei et al., 2017; Lai et al., 2018; Liu and Zhang, 2022; Ye et al., 2022) involving 788 university students were analyzed. Because the outcome was reported using the same measurement units, MD was chosen as the effect size. All standing long jump measurements are expressed in meters (m). The heterogeneity assessment revealed high variability (I² = 79.1%). Variance partitioning indicated that nearly all heterogeneity was attributable to within-study variability (100%), with negligible between-study variance (see **Supplementary File**). The meta-analysis indicated that traditional Chinese mind-body training led to a statistically significant improvement in standing long jump performance (MD = 0.11, 95% CI: 0.02 to 0.20, P = 0.021) (**Figure 8**).

To address the significant heterogeneity, sensitivity analyses were conducted. The Egger’s regression test was omitted because the number of studies was limited (k = 5, which is less than 10). Influence diagnostics did not identify any studies with an outsized impact. Leave-one-out analyses indicated consistent effect directions, but all 95% confidence intervals encompassed zero, suggesting that the results were stable but the evidence was weak (see **Supplementary File**).

In summary, the three-level meta-analysis suggested that traditional Chinese mind–body training may improve standing long jump performance among university students (MD = 0.11, 95% CI: 0.02 to 0.20, P = 0.021). However, the conventional two-level random-effects model used for supplementary comparison did not reach statistical significance (MD = 0.11, 95% CI: −0.01 to 0.22, P = 0.066), although the effect direction remained consistent. These findings indicate that the standing long jump result was sensitive to the analytical model and should therefore be interpreted with caution. The GRADE framework rated the certainty of evidence as low (see **Supplementary File**).

#### 3.4.8 50-Meter sprint

A total of two studies (Liu and Zhang, 2022; Ye et al., 2022) involving 386 university students were analyzed. Because the studies used the same measurement tools, MD was chosen as the effect size. The heterogeneity assessment indicated moderate to high variability (I² = 66.8%). The combined results indicated that traditional Chinese mind-body training did not lead to a statistically meaningful improvement in 50-meter sprint times for university students (MD = −1.03, 95% CI: −3.00 to 0.95, P = 0.307) (see **Supplementary File**).

Sensitivity analyses using a leave-one-out approach revealed that the overall effect direction remained the same. However, there was substantial variation (MD ranging from −0.77 to −2.02), with none of the estimates achieving statistical significance. This indicates instability in the results and weak evidence strength (see **Supplementary File**).

In summary, conventional mind-body practices from Chinese traditions did not significantly improve 50-meter sprint times among university students (MD = −1.03, 95% CI: −3.00 to 0.95, P = 0.307). According to the GRADE framework, the evidence was assessed as very low in reliability, indicating a lack of compelling evidence that this training positively affects sprinting performance (see **Supplementary File**).

#### 3.4.9 Pull-ups/sit-ups

**Figure 9:**
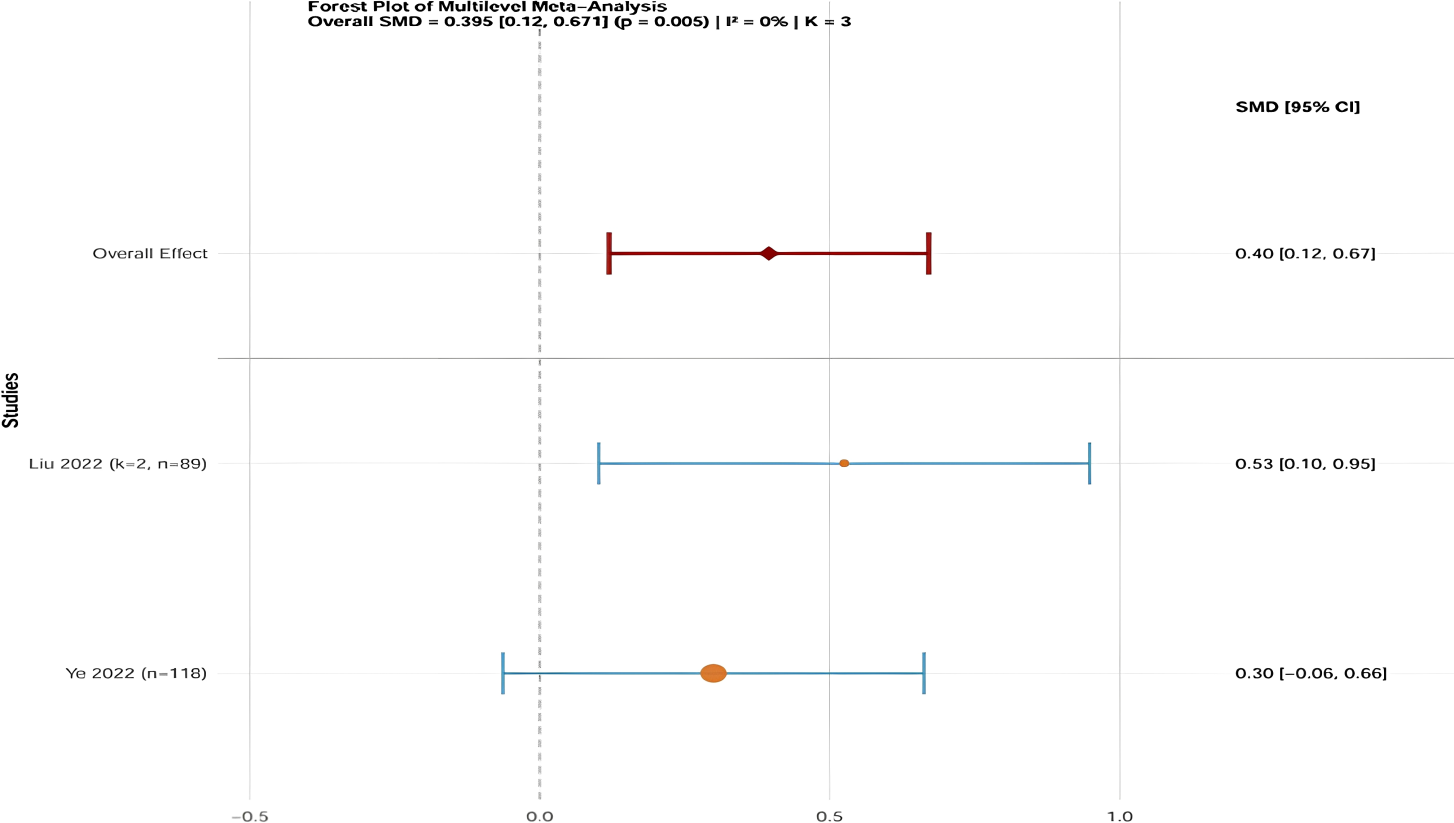
Forest plot of the effect of traditional Chinese mind-body training on Pull-ups/sit-ups in university students.

A total of 386 university students participated in two studies (Liu and Zhang, 2022; Ye et al., 2022). Due to variations in measurement tools, SMD was chosen as the effect size. Because no heterogeneity was observed (I² = 0%), a fixed-effect model was applied. The combined analysis showed that traditional Chinese mind-body training significantly improved pull-up and sit-up performance compared to the control group (SMD = 0.40, 95% CI: 0.12 to 0.67, P = 0.005) (**Figure 9**).

In conclusion, traditional Chinese mind-body training led to a statistically significant improvement in pull-up and sit-up performance among university students (SMD = 0.40, 95% CI: 0.12 to 0.67, P = 0.005). This finding is supported by low-quality evidence according to the GRADE framework (see **Supplementary File**). A two-level random-effects model was further conducted as a sensitivity analysis, and the results remained consistent with the main analysis (SMD = 0.40, 95% CI: 0.12 to 0.67, P = 0.005) (see **Supplementary File**).

#### 3.4.10 SLST

A total of 140 university students participated in two studies (Wu and Hou, 2015; Wang, 2021). Because the same measurement tools were used, MD was chosen as the effect size. The heterogeneity analysis revealed moderate to high heterogeneity across studies (I² = 65.3%). The combined findings indicated that traditional Chinese mind-body training did not yield a statistically significant improvement in SLST performance compared with the control group (MD = −0.39, 95% CI: −1.13 to 0.35, P = 0.299) (see **Supplementary File**).

In summary, the combined findings from two studies showed that conventional mind-body practices from Chinese traditions did not yield a meaningful improvement in SLST performance for university students (MD = −0.39, 95% CI: −1.13 to 0.35, P = 0.299). According to the GRADE evaluation, the level of certainty regarding this evidence was very low, indicating a lack of dependable evidence for a positive impact on balance performance (see **Supplementary File**).

### 3.5 Subgroup analysis and meta-regression results

Analyses of subgroups were conducted to thoroughly investigate the main moderating factors that affect the impact of traditional Chinese mind–body practices on the physical health of university students (**Table 2**). The predetermined subgroup factors included: (1) the type of mind–body practice; (2) comparator type; (3) intervention frequency; (4) session length; and (5) total intervention duration. The GRADE framework was used to assess the certainty of evidence for all subgroup findings (Guyatt et al., 2008). Overall, traditional Chinese mind-body exercises tended to improve students’ physical health. BMI showed significant subgroup differences for intervention frequency and session duration (P_8_ < 0.05), while other outcomes did not. These findings were exploratory and should be interpreted with caution.

**Table 2:** Subgroup analysis of traditional Chinese mind-body exercise on the level of physical health in university students.

Scale type	Dimensionality	K	N	I^2^(%)	Effect model	SMD/MD (95% CI)	GRADE	P	P_m_
Vital capacity	**Intervention mode 1**								0.070
	Tai Chi	6	523	36.3	Random	0.16 (-0.07, 0.39)	Very low	0.168	
	Baduanjin	4	504	76.6	Random	0.55 (0.04, 1.06)	Low	0.034^*^	
	Baduanjin+Yijinjing	2	89	0.0	Random	0.78 (0.34, 1.21)	Moderate	0.000^***^	
	**Intervention mode 2**								0.054
	Control-NoInt	11	552	76.2	Random	0.45 (0.17, 0.73)	Low	0.000^***^	
	Control-Int	4	147	0.0	Random	0.09 (-0.14, 0.33)	Very low	0.521	
	**Frequency**								0.113
	3 times/week	2	165	0.0	Random	0.47 (0.16, 0.78)	Moderate	0.003^**^	
	4 times/week	4	278	18.6	Random	0.16 (-0.11, 0.43)	Very low	0.258	
	5 times/week	7	784	81.6	Random	0.34 (-0.01, 0.69)	Very low	0.054	
	7 times/week	2	89	0.0	Random	0.78 (0.34, 1.21)	Low	0.000^***^	
	**Session Duration**								0.137
	45 min/session	3	220	83.7	Random	0.76 (0.05, 1.46)	Low	0.035^*^	
	60 min/session	9	884	62.9	Random	0.32 (0.09, 0.56)	Moderate	0.007^**^	
	90 min/session	2	132	0.0	Random	-0.07 (-0.42, 0.27)	Very low	0.679	
	**Intervention cycle**								0.446
	10 weeks	2	120	89.3	Random	0.96 (-0.22, 2.14)	Very low	0.112	
	12 weeks	5	611	72.5	Random	0.28 (-0.05, 0.61)	Very low	0.093	
	13 weeks	2	180	75.1	Random	0.36 (-0.28, 0.99)	Very low	0.268	
	16 weeks	2	145	29.5	Random	0.35 (-0.05, 0.74)	Very low	0.085	
BMI	**Intervention mode 1**								0.182
	Tai Chi	5	331	66.9	Random	-0.43 (-1.21, 0.26)	Low	0.181	
	Baduanjin	2	208	88.9	Random	-1.36 (-4.58, 1.86)	Low	0.407	
	Baduanjin+Yijinjing	2	256	50.5	Random	-2.01 (-3.27, -0.76)	Moderate	0.002^**^	
	**Subject type**								0.002^**^
	Normal students	7	233	65.7	Random	-1.16 (-1.96, -0.36)	Moderate	0.005^**^	
	Overweight students	2	54	39.7	Random	-0.83 (-1.43, -0.23)	Low	0.007^**^	
	**Frequency**								0.000^***^
	3x/week	4	320	60.1	Random	-1.02 (-1.69, -0.36)	Moderate	0.003^**^	
	4x/week	2	158	0	Random	-0.18 (-0.98, 0.63)	Moderate	0.670	
	7x/week	2	256	50.5	Random	-2.01 (-3.27, -0.76)	Moderate	0.002^**^	
	**Intervention cycle**								0.316
	12weeks	6	626	72.8	Random	-1.27 (-2.06, -0.47)	Moderate	0.002^**^	
	16weeks	2	140	59.7	Random	0.43 (-1.21, 2.07)	Low	0.607	
Standing long jump	**Intervention mode 1**								0.506
	Baduanjin	3	384	69.3	Random	0.06 (-0.03, 0.16)	Very low	0.189	
	Baduanjin+Yijinjing	2	89	92.2	Random	0.19 (-0.02, 0.40)	Very low	0.071	
	**Frequency**								0.333
	5 times/week	3	386	66.6	Random	0.08 (-0.03, 0.19)	Very low	0.173	
	7 times/week	2	89	92.2	Random	0.19 (-0.02, 0.40)	Very low	0.071	
	**Intervention cycle**								0.610
	12 weeks	4	413	86.3	Random	0.10 (-0.00, 0.21)	Very low	0.052	
	13 weeks	2	180	0.0	Random	0.14 (0.06, 0.23)	Moderate	0.001^**^	
RHR	**Intervention mode 1**								0.719
	Tai Chi	5	416	66.5	Random	-1.32 (-3.07, 0.43)	Very low	0.140	
	Baduanjin	4	419	0.0	Random	-0.93 (-2.25, 0.39)	Very low	0.166	
	**Intervention mode 2**								0.156
	Control-NoInt	7	361	9.7	Random	-1.79 (-2.75, -0.84)	Low	0.000^***^	
	Control-Int	2	60	0.0	Random	0.35 (-1.11, 1.81)	Very low	0.64	
	**Subject type**								0.844
	Normal students	6	303	0.0	Random	-1.15 (-2.36, 0.06)	Moderate	0.06	
	Physically Weak/Non-PE Majors students	3	111	81.9	Random	-1.41 (3.67, 0.85)	Very Low	0.22	
	**Frequency**								0.069
	3 times/week	3	223	43.1	Random	-2.22 (-3.53, -0.91)	Moderate	0.001^**^	
	5 times/week	4	524	0.0	Random	-0.40 (-2.24, 1.44)	Very low	0.667	
	**Session duration**								0.094
	45min/session	2	120	n/a	Random	-0.84(-3.44,1.76)	Very Low	0.525	
	60 min/session	6	655	24.1	Random	-1.75 (-2.86, -0.65)	Moderate	0.002^**^	
	**Intervention cycle**								0.262
	10weeks	2	120	n/a	Random	-0.84(-3.44,1.76)	Very Low	0.525	
	12weeks	2	404	n/a	Random	0.04(-2.57,2.64)	Very Low	0.978	
	16 weeks	2	158	70.2	Random	-2.37 (-4.57, -0.16)	Moderate	0.035^*^	
Sit-and-reach test	**Intervention mode 1**								0.321
	Tai Chi	4	240	58.1	Random	2.96 (1.09, 4.84)	Moderate	0.002^**^	
	Baduanjin	3	384	68.9	Random	1.97 (-0.60, 4.54)	Very low	0.133	
	Baduanjin+Yijinjing	2	89	41.5	Random	4.29 (1.36, 7.21)	Moderate	0.004^**^	
	**Intervention mode 2**								0.144
	Control-NoInt	8	349	63.4	Random	3.45 (2.06, 4.85)	Moderate	0.000^***^	
	Control-Int	2	77	45.1	Random	1.40 (-1.23, 4.02)	Very low	0.296	
	**Subject type**								0.159
	Normal students	8	353	64.5	Random	2.74 (1.21, 4.28)	Moderate	0.000^***^	
	Overweight students	2	54	0.0	Random	4.26 (2.74, 5.78)	Low	0.000^***^	
	**Frequency**								0.373
	1 times/week	2	89	41.5	Random	4.29 (1.36, 7.21)	Moderate	0.004^**^	
	3 times/week	2	108	0.0	Random	4.26 (2.74, 5.78)	Moderate	0.000^***^	
	4 times/week	3	250	48	Random	2.23 (0.07, 4.39)	Moderate	0.043^*^	
	5 times/week	3	386	83.3	Random	2.40 (-0.78, 5.57)	Very low	0.139	
	**Session duration**								0.173
	60 min/session	8	701	62.4	Random	3.45 (2.06, 4.85)	Moderate	0.000^***^	
	90 min/session	2	132	45.1	Random	1.40 (-1.23, 4.02)	Very low	0.296	
	**Intervention cycle**								0.250
	12 weeks	6	521	69.3	Random	3.35 (1.55, 5.15)	Moderate	0.000^***^	
	15 weeks	2	132	45.1	Random	1.40 (-1.23, 4.02)	Very low	0.296	

K, number of combined effect sizes; N, total sample size across studies; I^2^, inconsistency index; SMD, standardized mean difference; MD, mean difference; Statistical significance is assumed if the 95% Confidence Interval (95% CI) does not include zero. GRADE represents the certainty of evidence (High, Moderate, Low, Very low); P, Statistical significance p-value; Pm, Subgroup interaction p-value; times/week, Times per week; min/session, Minutes per session; Intervention modes: Control-NoInt, a control group receiving no active intervention; Control-Int, a control group receiving another active intervention (e.g., conventional exercise, health education). n/a, not applicable (N/A); *p < 0.05; **p < 0.01; ***p < 0.001.

Bold text indicates subgroup category headings.

**Figure 10:**
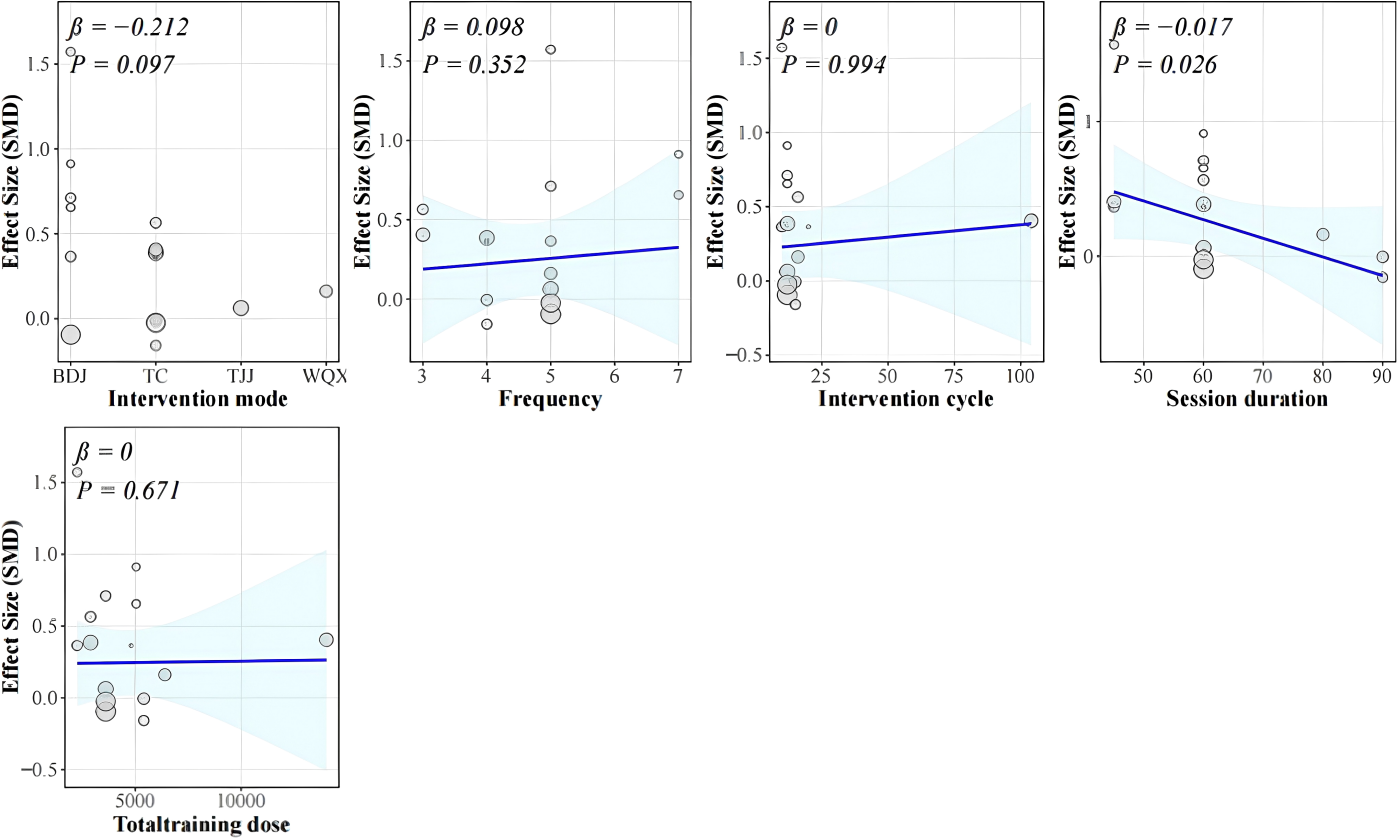
Meta-regression of potential moderators on the effect of traditional Chinese mind-body training on vital capacity in university students. Blue lines represent regression slopes (β) with 95% confidence intervals; circles represent individual study effect sizes.

For vital capacity, Baduanjin combined with Yijinjing showed the largest effect (SMD = 0.78, P = 0.000), Baduanjin alone had a moderate effect (SMD = 0.55, P = 0.034), and Tai Chi had a smaller effect (SMD = 0.16, P = 0.168). Regarding comparator type, outcomes compared with no-intervention controls were generally statistically significant (SMD = 0.45, P < 0.05). In contrast, outcomes compared with active intervention controls were mostly non-significant, highlighting that control type influences effect size. Among intervention frequencies, training three times/week (SMD = 0.47, P = 0.003) and seven times/week (SMD = 0.78, P = 0.000) were associated with statistically significant improvements. Regarding session duration, 45-minute sessions (SMD = 0.76, P = 0.035) and 60-minute sessions (SMD = 0.32, P = 0.007) showed significant improvements, whereas 90-minute sessions did not. Meta-regression indicated that session duration was the only significant moderator (β = −0.017, P = 0.026), suggesting that longer sessions were associated with slightly smaller improvements, whereas other moderators were not significant (**Figure 10**).

For BMI, Baduanjin combined with Yijinjing demonstrated a significant effect (MD = −2.01, P = 0.002), whereas Tai Chi and Baduanjin alone did not reach significance. Significant effects were found in both normal (MD = −1.16, P = 0.005) and overweight (MD = −0.83, P = 0.007) students, with a significant difference between subgroups (P for interaction = 0.002). Greater reductions were associated with training three times per week (MD = −1.02, P = 0.003) and seven times per week (MD = −2.01, P = 0.002), whereas four times per week was not significant. Regarding intervention duration, the 12-week subgroup showed a significant improvement (MD = −1.27, P = 0.002), whereas the 16-week subgroup did not.

For the standing long jump, subgroup analyses indicated that neither intervention mode nor training frequency reached statistical significance. Regarding intervention duration, significant improvements were observed for 13-week interventions (MD = 0.14, P = 0.001), whereas 12-week interventions showed a borderline effect (P = 0.052).

For resting heart rate, Tai Chi and Baduanjin showed non-significant downward trends overall. Subgroup analyses showed larger reductions compared with no-intervention controls (MD = −1.79, P = 0.000), whereas comparisons with active controls were mostly non-significant. No significant effects were observed by subject type. Greater reductions were associated with training three times per week (MD = −2.22, P = 0.001), 60-minute sessions (MD = −1.75, P = 0.002), and a 16-week intervention period (MD = −2.37, P = 0.035).

For the sit-and-reach test, Baduanjin combined with Yijinjing (MD = 4.29, P = 0.004) and Tai Chi (MD = 2.96, P = 0.002) showed significant improvements. Greater gains were observed compared with no-intervention controls (MD = 3.45, P = 0.000), whereas comparisons with active controls were mostly non-significant. Significant improvements were observed in both normal (MD = 2.74, P = 0.000) and overweight (MD = 4.26, P = 0.000) students, with no significant difference between subgroups (P for interaction = 0.159). Significant improvements were associated with training 1, 3, or 4 sessions per week, 60-minute sessions, and a 12-week intervention period.

Overall, subgroup analyses indicate that traditional Chinese mind-body exercises may improve several aspects of physical health in university students. Combined Baduanjin and Yijinjing showed the largest benefits, followed by Baduanjin alone, while Tai Chi generally showed smaller or non-significant effects. Outcomes were generally significant compared with no-intervention controls but mostly non-significant compared with active controls, suggesting that these interventions may provide modest or comparable effects relative to conventional exercise while consistently outperforming no-intervention conditions. Greater improvements were typically observed with moderate session durations (45–60 min), training frequencies of 3–7 sessions per week, and intervention periods of approximately 12–16 weeks, although results varied across outcomes. These findings are exploratory and should be interpreted cautiously, particularly for outcomes based on a limited number of studies.

## 4 Discussion

This comprehensive meta-analysis and systematic review examined the impact of traditional Chinese mind-body techniques on the physical health of university students, assessing changes in 11 key health metrics, including vital capacity, BMI, and RHR. The findings suggest that traditional Chinese mind-body practices were associated with improvements in several outcomes, including vital capacity (SMD = 0.34, 95% CI: 0.11 to 0.56, P = 0.003), BMI (MD = −0.77, 95% CI: −1.48 to −0.06, P = 0.034), RHR (MD = −1.16, 95% CI: −2.29 to −0.04, P = 0.043), sit-and-reach test (MD = 2.99, 95% CI: 1.59 to 4.38, P = 0.000) and performance in pull-ups/sit-ups (SMD = 0.40, 95% CI: 0.12 to 0.67, P = 0.005), suggesting potential benefits for health promotion initiatives targeting university students. Although the three-level model suggested an improvement in standing long jump, this finding was not confirmed in the two-level sensitivity analysis and should therefore be interpreted with caution. Some measures, such as handgrip strength, step test index, 50-meter Sprint, and single-leg balance, did not show significant improvements. This may be related to the relatively moderate intensity of traditional Chinese mind-body exercises and the limited upper-limb resistance or explosive training stimuli involved in these practices (Wan et al., 2025).

These findings are broadly consistent with previous research indicating that practices such as Tai Chi and Qigong can improve flexibility, cardiorespiratory function, and balance in university populations (Webster et al., 2016; Wehner et al., 2021; Lin et al., 2022; Qi et al., 2022). For instance, a randomized controlled trial by Zheng et al. reported that a 12-week Tai Chi program significantly improved flexibility in college students compared with a control group (sit-and-reach: 14.09 cm vs. 12.88 cm, *P* = 0.039) (Zheng et al., 2015). In addition, a systematic review including 76 studies and 9,263 university students found that Tai Chi interventions were associated with improvements in flexibility, lung capacity, and balance among tertiary student populations. By integrating slow, controlled, and rhythmic movements with coordinated breathing, traditional Chinese mind-body exercises may enhance respiratory efficiency, cardiovascular regulation, and neuromuscular coordination (Zou et al., 2018; Wang et al., 2023; Qu et al., 2024). The inclusion of stretching, twisting, and balance-focused movements may further promote joint mobility, muscular endurance, and overall motor control, which could explain the observed improvements in flexibility and muscular endurance outcomes such as pull-ups and sit-ups. In addition, the meditative and attentional components of these practices may reduce academic stress and support physiological balance, thereby contributing to overall physical well-being (Wang et al., 2010).

Subgroup analyses and meta-regression provided additional exploratory insights into potential moderators of intervention effectiveness. Interventions combining Baduanjin and Yijinjing, with session durations of approximately 45–60 minutes and intervention periods of around 12–16 weeks, tended to be associated with greater improvements across several outcomes, although findings were not entirely consistent. Participant-level analyses indicated that both normal/healthy and overweight students may benefit from these interventions, although the magnitude of effects varied across populations and outcomes. Meta-regression further suggested that session duration may partly explain variability in vital capacity outcomes. However, these findings should be interpreted cautiously because the number of studies in some subgroups was limited, and considerable heterogeneity across interventions existed. In addition, variability in comparator conditions (e.g., no intervention, usual activity, or alternative exercise) may have influenced the magnitude of the pooled effects.

Compared with other exercise interventions commonly used in university populations, such as high-intensity interval training or resistance training, traditional Chinese mind-body exercises generally involve lower movement intensity and slower, controlled motion patterns (Wan et al., 2025; Yang et al., 2025; Yin et al., 2025). Previous meta-analyses have shown that high-intensity interval training can produce substantial improvements in aerobic capacity and physical fitness among university students, due to the greater physiological load of these programs (Yang et al., 2025). Similarly, resistance training has been demonstrated to significantly enhance muscular strength and physical performance in various populations (Wehner et al., 2021). In contrast, the improvements observed in the present meta-analysis were generally moderate, highlighting the need to consider their practical and clinical relevance. Although statistically significant reductions in BMI (MD = −0.77) were observed, such changes may not necessarily correspond to substantial clinical benefit at the individual level. Some studies on low-level weight loss suggest improvements in cardiovascular and metabolic indices even with modest reductions in BMI (Dhar et al., 2025). However, large cohort studies indicate that clinically meaningful cardiovascular risk reductions are usually associated with larger BMI category differences, and the long-term impact of small changes on hard outcomes remains uncertain (Khan et al., 2020).

Overall, this meta-analysis suggests that traditional Chinese mind-body exercises may improve several aspects of physical health among university students. Another issue to consider when interpreting these findings is how the intervention characteristics align with current physical activity guidelines. According to the World Health Organization’s physical activity guidelines, adults are recommended to accumulate at least 150–300 minutes of moderate-intensity physical activity per week to achieve substantial health benefits (Bull et al., 2020). In the studies included in the present review, most interventions consisted of sessions lasting approximately 45–60 minutes performed three to seven times per week, which may approach the recommended duration but often involve relatively moderate or low exercise intensity. Therefore, although statistically significant improvements were observed for several outcomes, it remains uncertain whether traditional Chinese mind-body practices alone can fully meet the recommended physical activity levels required for optimal long-term health promotion.

## 5 Limitations

Several limitations should be acknowledged. First, the included studies were limited to publications in Chinese and English, which may introduce language bias. Second, the methodological quality of some trials was suboptimal, and blinding was often infeasible given the nature of traditional Chinese mind-body training, potentially introducing performance bias into outcomes dependent on participant effort. These issues were considered in the GRADE assessment, and outcomes with a higher risk of bias were downgraded accordingly. In addition, variability in intervention types and protocols across studies, together with the limited number of studies for some outcomes, may have influenced the reliability and generalizability of the pooled estimates. Although several outcomes reached statistical significance, the long-term clinical relevance of these effects remains uncertain. Finally, most studies were conducted in East Asia, particularly China and Thailand, where traditional mind-body exercises are integrated into university programs; randomized controlled trials on these interventions in other countries are extremely limited, which may constrain the generalizability of these findings to other cultural contexts.

## 6 Conclusions

This comprehensive review and meta-analysis suggests that traditional Chinese mind-body practices may improve several physical health indicators among university students, including vital capacity, BMI, resting heart rate, sit-and-reach test, and pull-up/sit-up performance. However, no significant improvements were observed for handgrip strength, step test index, 50-meter Sprint, or single-leg balance. For the standing long jump, the result was not robust in the two-level sensitivity analysis and should therefore be interpreted with caution. Further large-scale and methodologically rigorous randomized controlled trials are needed to confirm these findings.

## Data Availability

The raw data supporting the conclusions of this article will be made available by the authors, without undue reservation.
