# Supplementary material for: The effects of traditional Chinese mind-body training on physical health in university students: a multilevel meta-analysis
Source: Front Physiol. 2026 Apr 10;17:1792981. doi: 10.3389/fphys.2026.1792981 (PMC13106537; doi:10.3389/fphys.2026.1792981)
Supplement: Supplementary file 1 [file Table1.docx]

**Table of Contents**

[**Supplementary document Retrieval Strategy** 4](#_Toc223780383)

[**Quality Assessment** 5](#_Toc223780384)

[**Study selection consistency and Cohen’s κ** 5](#_Toc223780385)

[**Inter-Rater Agreement Results for Each ROB2 Domain** 6](#_Toc223780386)

[**Vital capacity** 6](#_Toc223780387)

[Variance decomposition and multilevel I². 6](#_Toc223780388)

[Egger’s Test Funnel Plot for Publication Bias Assessment. 7](#_Toc223780389)

[Influence diagnostics for included studies using standardized residuals and Cook’s distance. 7](#_Toc223780390)

[Figure of the Initial Sensitivity Analysis. 8](#_Toc223780391)

[Trim-and-Fill Funnel Plot. 8](#_Toc223780392)

[Forest plot of traditional Chinese mind body training effects on vital capacity in university students. 9](#_Toc223780393)

[Summary of Findings — Effects of Chinese Traditional Mind-Body Practices on Vital Capacity in College Students. 9](#_Toc223780394)

[**BMI** 10](#_Toc223780395)

[Variance decomposition and multilevel I². 10](#_Toc223780396)

[Egger’s Test Funnel Plot for Publication Bias Assessment. 11](#_Toc223780397)

[Influence diagnostics for included studies using standardized residuals and Cook’s distance. 11](#_Toc223780398)

[Figure of the Initial Sensitivity Analysis. 12](#_Toc223780399)

[Trim-and-Fill Funnel Plot. 12](#_Toc223780400)

[Forest plot of traditional Chinese mind body training effects on BMI in university students. 13](#_Toc223780401)

[Summary of Findings — Effects of Chinese Traditional Mind-Body Practices on BMI in College Students. 13](#_Toc223780402)

[**Resting Heart Rate** 14](#_Toc223780403)

[Variance decomposition and multilevel I². 14](#_Toc223780404)

[Influence diagnostics for included studies using standardized residuals and Cook’s distance. 15](#_Toc223780405)

[Figure of the Initial Sensitivity Analysis. 15](#_Toc223780406)

[Trim-and-Fill Funnel Plot. 15](#_Toc223780407)

[Forest plot of traditional Chinese mind body training effects on Resting Heart Rate in university students. 16](#_Toc223780408)

[Summary of Findings — Effects of Chinese Traditional Mind-Body Practices on Resting Heart Rate in College Students. 16](#_Toc223780409)

[**Step Test Index** 17](#_Toc223780410)

[Forest plot showing the effect of traditional Chinese physical and mental training on the Step Test Index of college students. 17](#_Toc223780411)

[Influence diagnostics for included studies using standardized residuals and Cook’s distance. 18](#_Toc223780412)

[Figure of the Initial Sensitivity Analysis. 18](#_Toc223780413)

[Summary of Findings — Effects of Chinese Traditional Mind-Body Practices on Step Test Index in College Students. 18](#_Toc223780414)

[**Sit-and-reach test** 20](#_Toc223780415)

[Variance decomposition and multilevel I². 20](#_Toc223780416)

[Egger’s Test Funnel Plot for Publication Bias Assessment. 20](#_Toc223780417)

[Figure of the Initial Sensitivity Analysis. 21](#_Toc223780418)

[Trim-and-Fill Funnel Plot. 22](#_Toc223780419)

[Forest plot of traditional Chinese mind body training effects on sit-and-reach test in university students. 22](#_Toc223780420)

[Summary of Findings — Effects of Chinese Traditional Mind-Body Practices on sit-and-reach test in College Students. 22](#_Toc223780421)

[**Handgrip Strength** 24](#_Toc223780422)

[Forest plot showing the effect of traditional Chinese physical and mental training on the Handgrip Strength of college students. 24](#_Toc223780423)

[Influence diagnostics for included studies using standardized residuals and Cook’s distance. 24](#_Toc223780424)

[Figure of the Initial Sensitivity Analysis. 25](#_Toc223780425)

[Summary of Findings — Effects of Chinese Traditional Mind-Body Practices on Handgrip Strength in College Students. 25](#_Toc223780426)

[**Standing Long Jump** 26](#_Toc223780427)

[Variance decomposition and multilevel I². 26](#_Toc223780428)

[Influence diagnostics for included studies using standardized residuals and Cook’s distance. 27](#_Toc223780429)

[Figure of the Initial Sensitivity Analysis. 27](#_Toc223780430)

[Forest plot of traditional Chinese mind body training effects on Standing Long Jump in university students. 28](#_Toc223780431)

[Summary of Findings — Effects of Chinese Traditional Mind-Body Practices on Standing Long Jump in College Students. 28](#_Toc223780432)

[**50-Meter Sprint** 29](#_Toc223780433)

[Forest plot showing the effect of traditional Chinese physical and mental training on the 50-Meter Sprint of college students. 29](#_Toc223780434)

[Figure of the Initial Sensitivity Analysis. 29](#_Toc223780435)

[Summary of Findings — Effects of Chinese Traditional Mind-Body Practices on 50-Meter Sprint in College Students. 30](#_Toc223780436)

[**Pull-ups/sit-ups** 31](#_Toc223780437)

[Forest plot of traditional Chinese mind body training effects on Pull-ups/Sit-ups in university students. 31](#_Toc223780438)

[Summary of Findings — Effects of Chinese Traditional Mind-Body Practices on Pull-ups/Sit-ups in College Students. 31](#_Toc223780439)

[**SLST** 32](#_Toc223780440)

[Forest plot showing the effect of traditional Chinese physical and mental training on the SLST of college students. 32](#_Toc223780441)

[Summary of Findings — Effects of Chinese Traditional Mind-Body Practices on SLST in College Students. 32](#_Toc223780442)

[**References** 34](#_Toc223780443)

# **Supplementary document Retrieval Strategy**

| **Data** | **Query** | **Results** |
| --- | --- | --- |
| **PubMed** | ((("Traditional Chinese mind-body exercise"[Title/Abstract] OR "mind-body exercise"[Title/Abstract] OR "Traditional Chinese exercise"[Title/Abstract] OR "Tai Chi"[Title/Abstract] OR "Qigong"[Title/Abstract] OR "Wu Qin Xi"[Title/Abstract] OR "Baduanjin"[Title/Abstract] OR "Yijinjing"[Title/Abstract] OR "Liuzijue"[Title/Abstract])) AND (("physical health"[Title/Abstract] OR "health"[Title/Abstract] OR "well-being"[Title/Abstract] OR "physical well-being"[Title/Abstract]))) AND (("college students"[Title/Abstract] OR "undergraduates"[Title/Abstract] OR "university students"[Title/Abstract])) | 50 |
| **Web of Science** | (“Traditional Chinese mind-body exercise” OR “mind-body exercise” OR “Traditional Chinese exercise” OR “Tai Chi” OR “Qigong” OR “Wu Qin Xi” OR “Baduanjin” OR “Yijinjing” OR “Liuzijue”) AND (“physical health” OR “health” OR “well-being” OR “physical well-being”) AND (“college students” OR “undergraduates” OR “university students”)) | 73 |
| **Embase** | ('traditional chinese mind-body exercise':ti,ab,kw OR 'mind-body exercise':ti,ab,kw OR 'traditional chinese exercise':ti,ab,kw OR 'tai chi':ti,ab,kw OR 'qigong':ti,ab,kw OR 'wu qin xi':ti,ab,kw OR 'baduanjin':ti,ab,kw OR 'yijinjing':ti,ab,kw OR 'liuzijue':ti,ab,kw) AND ('physical health':ti,ab,kw OR 'health':ti,ab,kw OR 'well-being':ti,ab,kw OR 'physical well-being':ti,ab,kw) AND ('college students':ti,ab,kw OR 'undergraduates':ti,ab,kw OR 'university students':ti,ab,kw) | 81 |
| **Cochrane Library** | “Traditional Chinese mind-body exercise” OR “mind-body exercise” OR “Traditional Chinese exercise” OR “Tai Chi” OR “Qigong” OR “Wu Qin Xi” OR “Baduanjin” OR “Yijinjing” OR “Liuzijue” in Title Abstract Keyword AND “physical health” OR “health” OR “well-being” OR “physical well-being” in Title Abstract Keyword AND “college students” OR “undergraduates” OR “university students” | 4486 |
| **CNKI** | （主题：传统运动 + 传统运动项目 + 传统运动疗法 + 传统运动功法）OR（主题：太极拳 + 太极拳运动 + 太极拳练习 + 太极拳训练 + 24式太极拳 + 陈氏太极拳）OR (八段锦 + 八段锦训练 + 健身气功八段锦 + 坐式八段锦) AND （主题：大学生 + 在校大学生）AND （身心健康 + 身心健康水平 + 身心健康状态 + 身心健康素质） | 68 |

Abbreviations: CNKI = Chinese National Knowledge Infrastructure.

# **Quality Assessment**

| **authors** | **D1** | **D2** | **D3** | **D4** | **D5** | **Overall** |
| --- | --- | --- | --- | --- | --- | --- |
| Yang, et al 2014 (1) | Some concerns | Some concerns | Low | Some concerns | Low | Some concerns |
| Zhu et al 2015 (2) | High | Some concerns | Low | Some concerns | Low | High |
| Li et al 2015 (3) | Low | Low | Low | Low | Low | Low |
| Zheng et al 2015 (4) | Low | Some concerns | Low | Low | Low | Low |
| Wu et al 2015 (5) | Some concerns | Some concerns | Low | Some concerns | Low | Some concerns |
| Wei et al 2017 (6) | Some concerns | Some concerns | Low | Some concerns | Low | Some concerns |
| Yuan et al 2017 (7) | Some concerns | Some concerns | Some concerns | Some concerns | Low | Some concerns |
| Lai et al 2018 (8) | Some concerns | Some concerns | Low | Some concerns | Low | Some concerns |
| Chang et al 2020 (9) | Some concerns | Some concerns | Some concerns | Some concerns | Low | Some concerns |
| Jiao et al 2021 (10) | Low | Some concerns | Low | Some concerns | Low | Some concerns |
| Wang et al 2021 (11) | Some concerns | Some concerns | Low | Some concerns | Low | Some concerns |
| Ye et al 2022 (12) | Low | Some concerns | Low | Low | Low | Low |
| Liu et al 2022 (13) | Some concerns | Some concerns | Low | Some concerns | Low | Some concerns |
| Zhang et al 2023 (14) | Low | Low | Low | Low | Low | Low |
| Niu et al 2023 (15) | Low | Some concerns | Low | Low | Low | Low |
| Wang et al 2024 (16) | Low | Some concerns | Low | Some concerns | Low | Some concerns |
| Niu et al 2024 (17) | Some concerns | Some concerns | Low | Low | Low | Some concerns |
| Liu et al 2025 (18) | Some concerns | Some concerns | Low | Some concerns | Low | Some concerns |

# **Study selection consistency and Cohen’s κ**

1. Title/abstract screening (records screened = 1807)

Independent 2×2 table (Y.C. vs J.X.): both include = 230; Y.C. include / J.X. exclude = 60; Y.C. exclude / J.X. include = 24; both exclude = 1493 (total n = 1807; reports sought for retrieval a + b + c = 314, with 254 records retained after consensus, consistent with the PRISMA flowchart).

- Observed agreement: $P_{o}=\left( 230+1493 \right)/1807=9535.$
- Expected agreement: $P_{e}\approx0.7431$.
- Cohen’s κ $\approx0.819$(more precisely 0.8190).
- Approximate standard error: $SE\approx0.019$.
- 95% CI: $\kappa\approx0.819$, 95% CI ≈ 0.782–0.856.
- z ≈ 43.1 (p ≪ 0.001).

Interpretation (Landis & Koch): κ ≈ 0.82, indicating substantial agreement.

2. Full-text screening (reports assessed = 248)

Independent 2×2 table (Y.C. vs J.X.): both include = 16; Y.C. include / J.X. exclude = 4; Y.C. exclude / J.X. include = 2; both exclude = 226 (total n = 248; a + b + c = 22, consistent with the PRISMA flowchart).

- Observed agreement: $P_{o}=(13+226)/248=0.9758$.
- Expected agreement: $P_{e}\approx0.8570$.
- Cohen’s κ $\approx0.831$(more precisely 0.8310).
- Approximate standard error: $SE\approx0.053$.
- 95% CI: ≈ 0.727–0.935 (upper bound truncated at 1.00 as customary when the normal approximation slightly exceeds 1).
- z ≈ 15.7 (p ≪ 0.001).

Interpretation (Landis & Koch): κ ≈ 0.83, indicating almost perfect agreement.

# **Inter-Rater Agreement Results for Each ROB2 Domain**

The table below displays the simple agreement rate, Cohen's Kappa, and Weighted Kappa calculated separately for each domain (D1-D5).

| **Domain** | **Simple Agreement Rate** | **Cohen’s κ** | **Weighted κ** | **Strength of Agreement** |
| --- | --- | --- | --- | --- |
| **D1 (Randomization process)** | 72.2% (13/18) | 0.50 | 0.63 | Moderate |
| **D2 (Deviations from intended interventions)** | 55.6% (10/18) | 0.11 | 0.28 | Fair |
| **D3 (Missing outcome data)** | 100.0% (18/18) | 1.00 | 1.00 | Almost Perfect |
| **D4 (Measurement of the outcome)** | 66.7% (12/18) | 0.36 | 0.54 | Moderate |
| **D5 (Selection of the reported result)** | 88.9% (16/18) | 0.77 | 0.87 | Very Good |

# **Vital capacity**


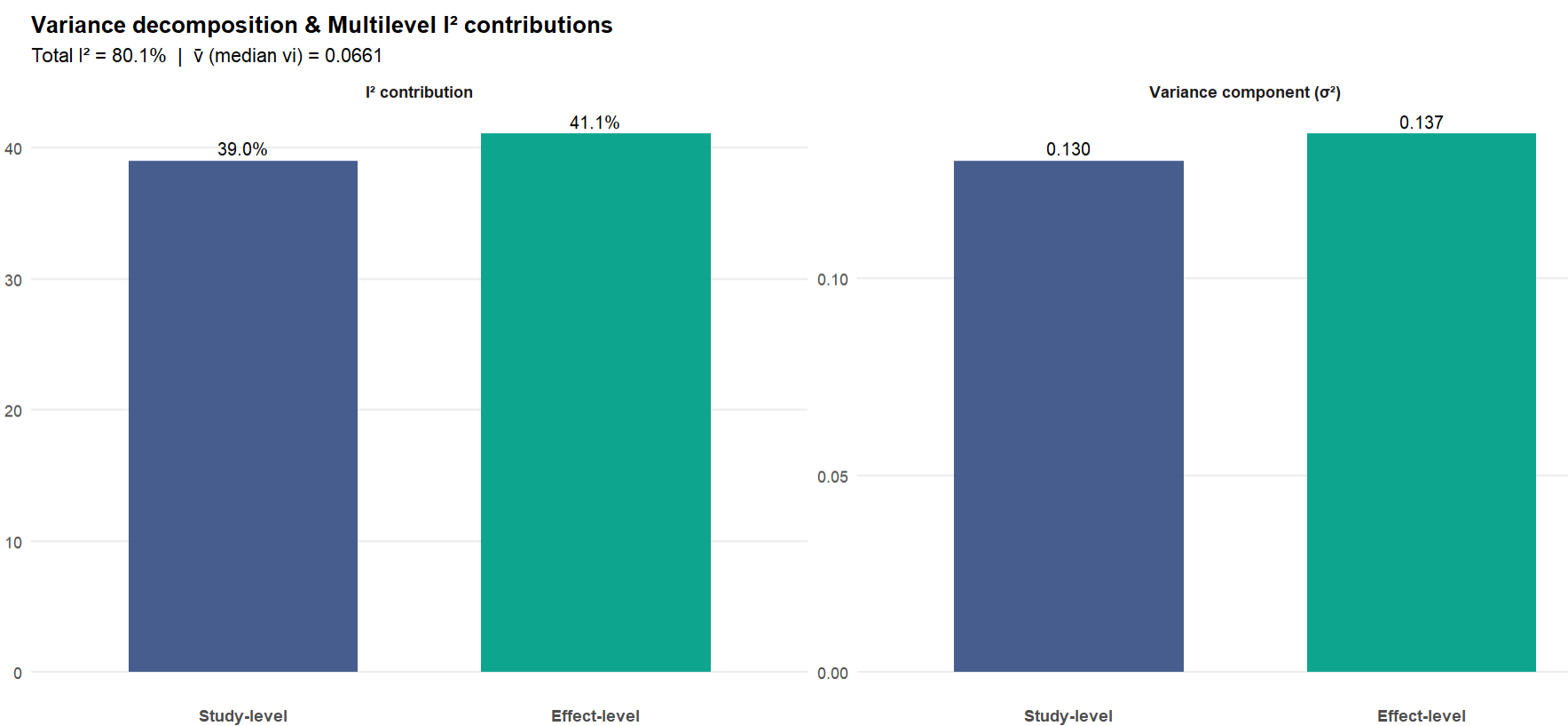


## Variance decomposition and multilevel I².


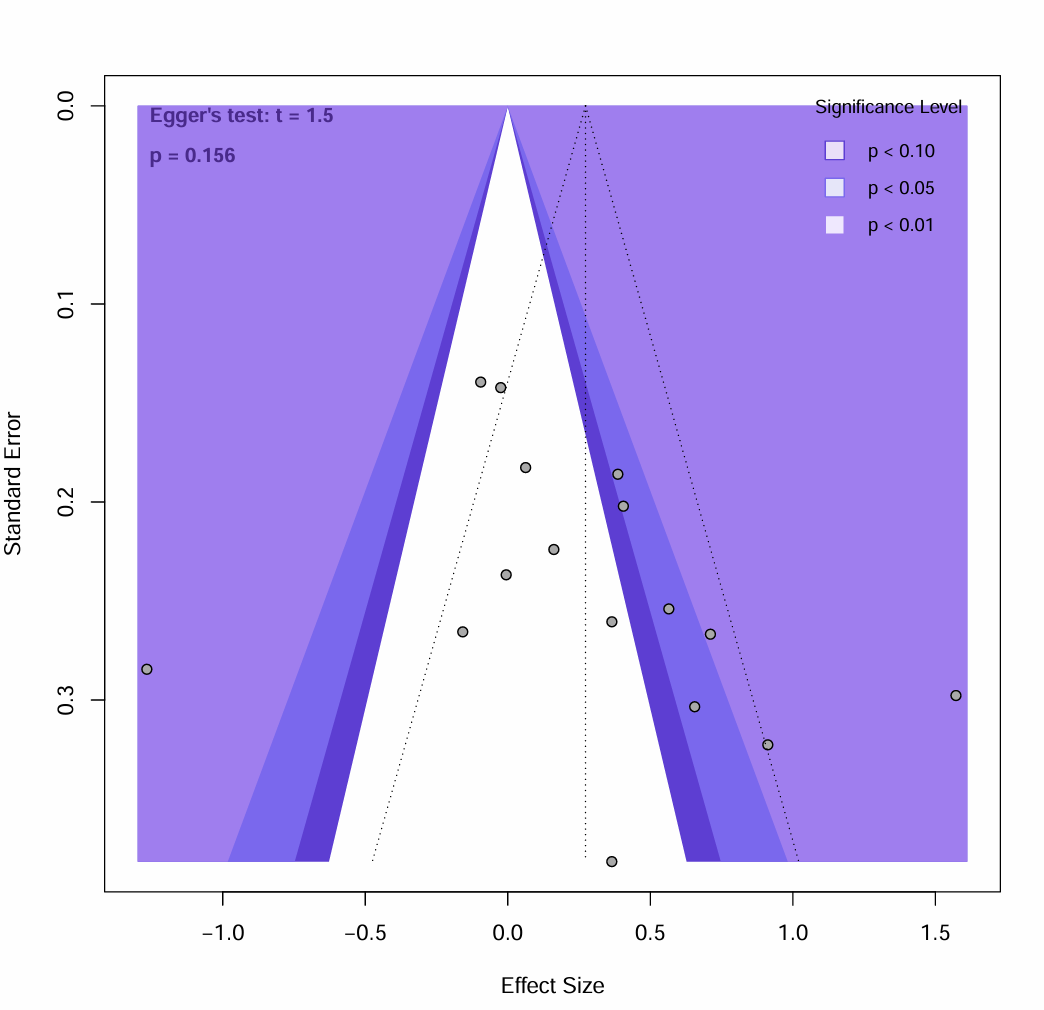


## Egger’s Test Funnel Plot for Publication Bias Assessment.


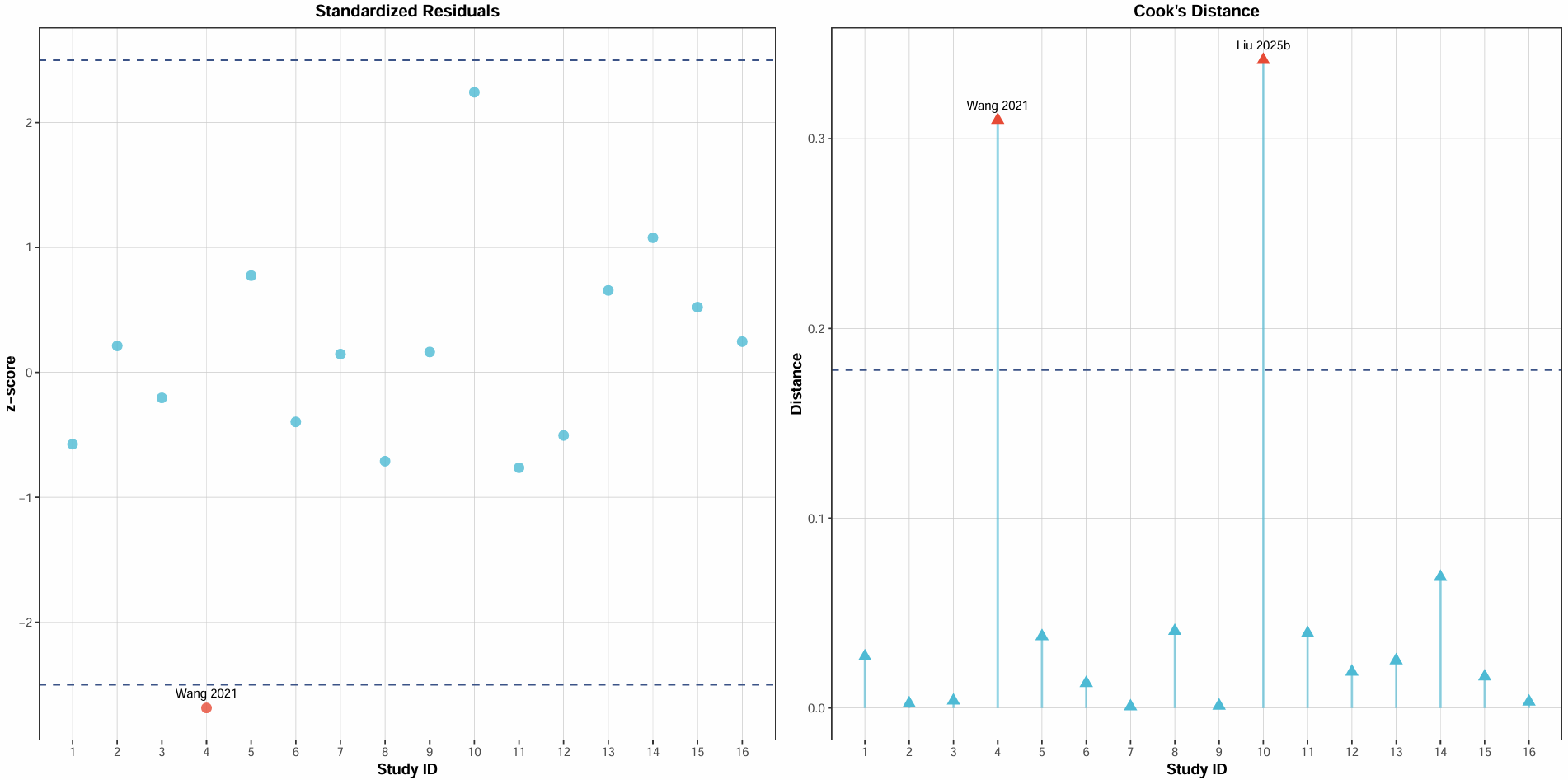


## Influence diagnostics for included studies using standardized residuals and Cook’s distance.

Wang et al. (2021) exceeded both thresholds, indicating high influence and potential outlier status.


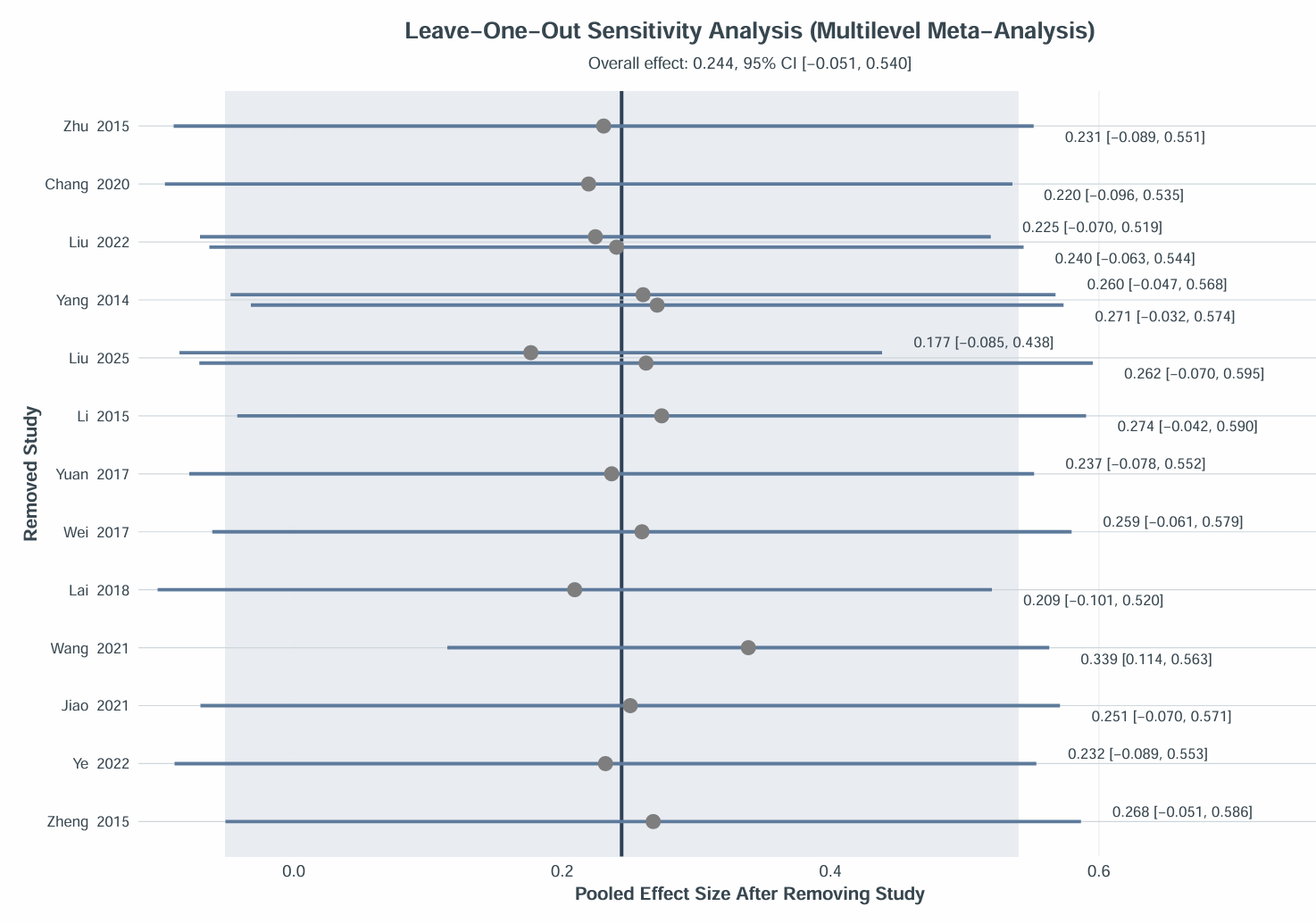


## Figure of the Initial Sensitivity Analysis.


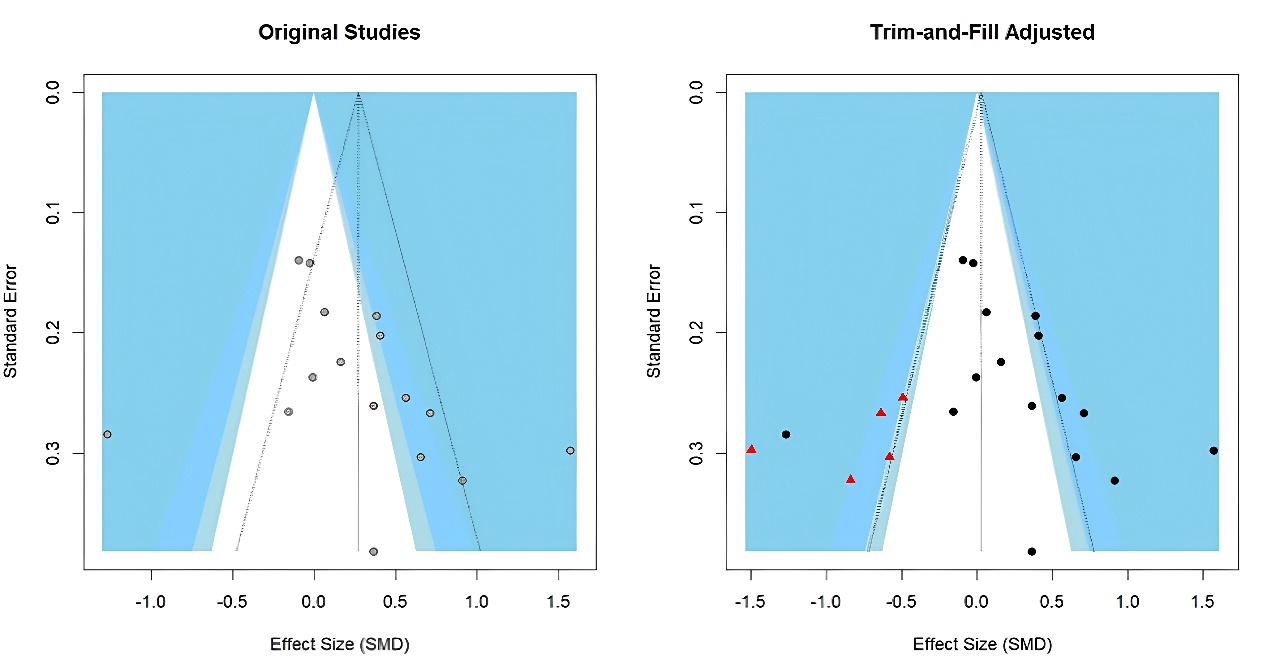


## Trim-and-Fill Funnel Plot.


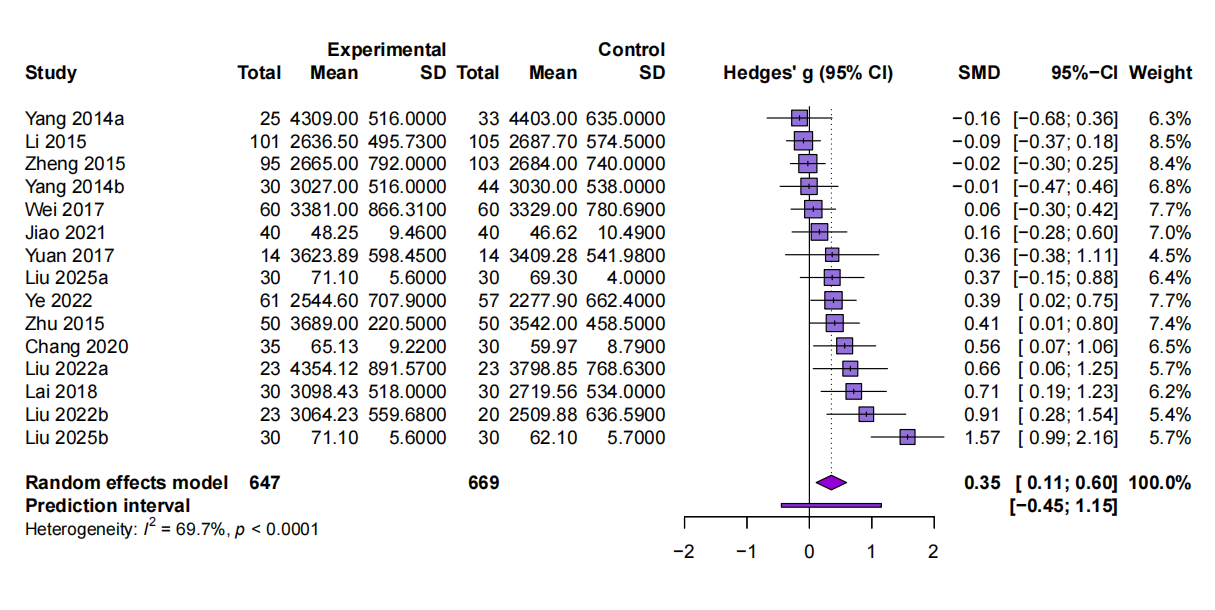


## Forest plot of traditional Chinese mind body training effects on vital capacity in university students.

## Summary of Findings — Effects of Chinese Traditional Mind-Body Practices on Vital Capacity in College Students.

| GRADE Domain | Judgment | Rationale (Strictly Based on the Original Article) |
| --- | --- | --- |
| Risk of Bias | Serious (Downgraded by 1 level) | Among the 13 included studies, most had "some concerns" in the randomization process (D1, Cohen’s κ = 0.50) and deviations from intended interventions (D2, Cohen’s κ = 0.18), with 1 study rated as high risk. Only missing outcome data (D3) and selective reporting (D5) were rated as low risk. |
| Inconsistency | Serious (Downgraded by 1 level) | Heterogeneity was very high (I² = 80.1% → 65.7% after excluding outliers). Although subgroup analysis identified the optimal intervention scheme, heterogeneity was not fully explained. |
| Indirectness | Not downgraded | The population, intervention, comparator, and outcomes were directly aligned with the research question, with no concerns regarding applicability. |
| Imprecision | Not downgraded | A total of 12 studies (1,639 participants) were included, with a pooled effect size of SMD = 0.34 (95%CI: 0.11 to 0.56, P = 0.003). The confidence interval did not include 0, and the sensitivity analysis showed stable results. |
| Publication Bias | Not downgraded | Egger’s test suggested a potential publication bias (P = 0.156), but after applying the trim-and-fill correction, the core conclusion remained unchanged, with a pooled SMD = 0.34 (95% CI: 0.11 to 0.56, P = 0.003), indicating no significant impact from potential publication bias. |
| Overall quality of evidence | Moderate | Evidence from randomized controlled trials initially started at a high level but was downgraded by two levels due to concerns regarding risk of bias and substantial heterogeneity across studies. No further downgrading was applied for indirectness, imprecision, or publication bias. Therefore, the overall certainty of the evidence was rated as **moderate** according to the GRADE framework. |

# **BMI**


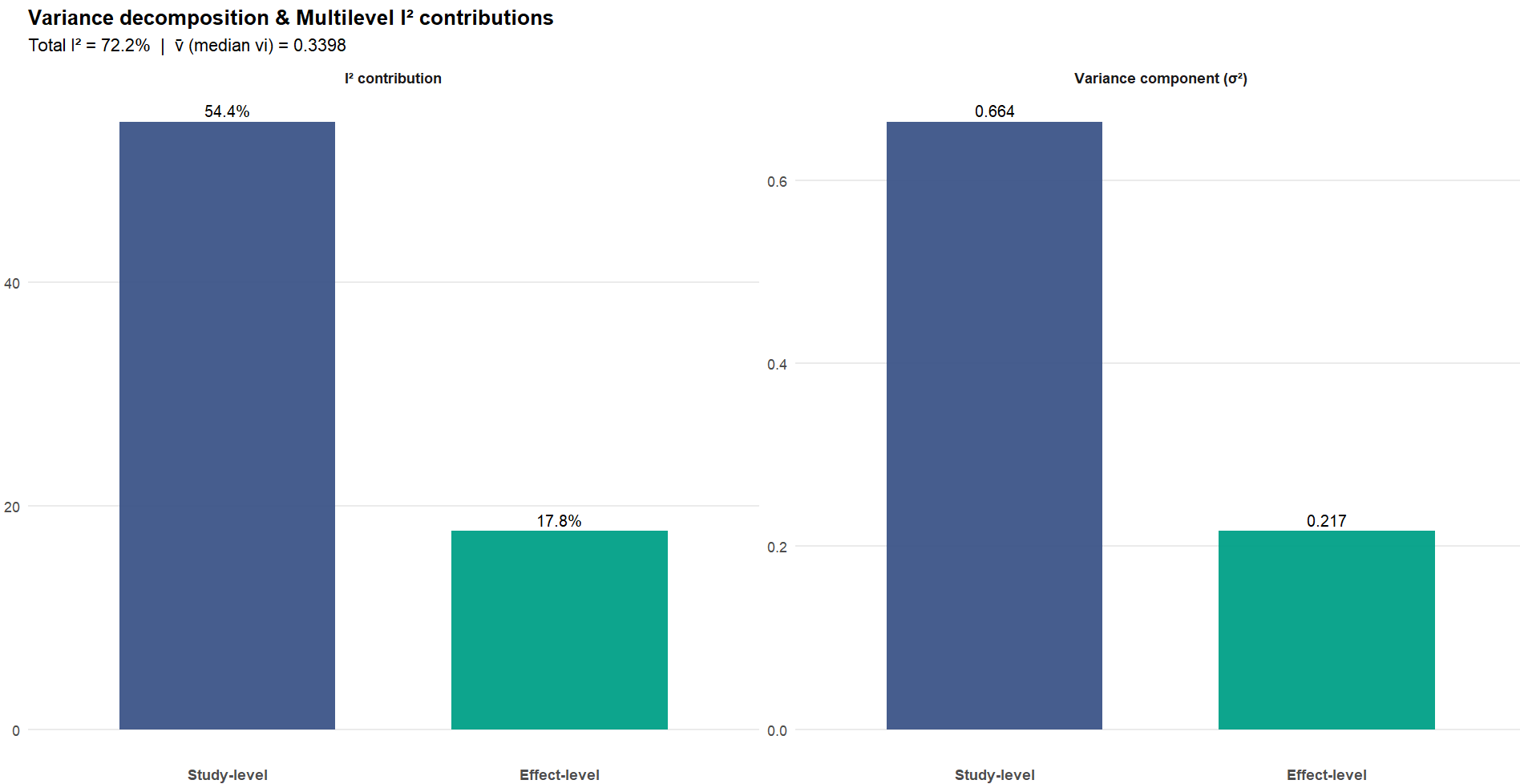


## Variance decomposition and multilevel I².


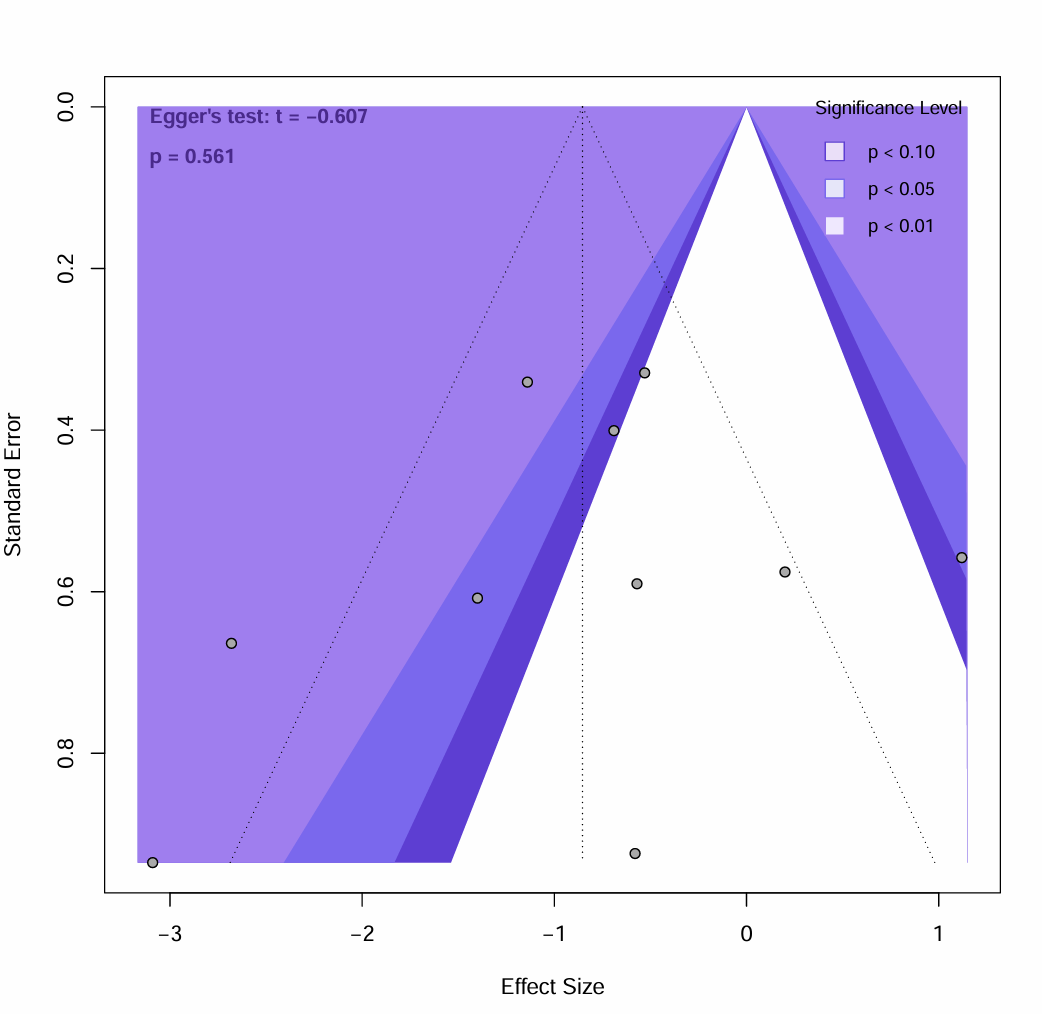


## Egger’s Test Funnel Plot for Publication Bias Assessment.


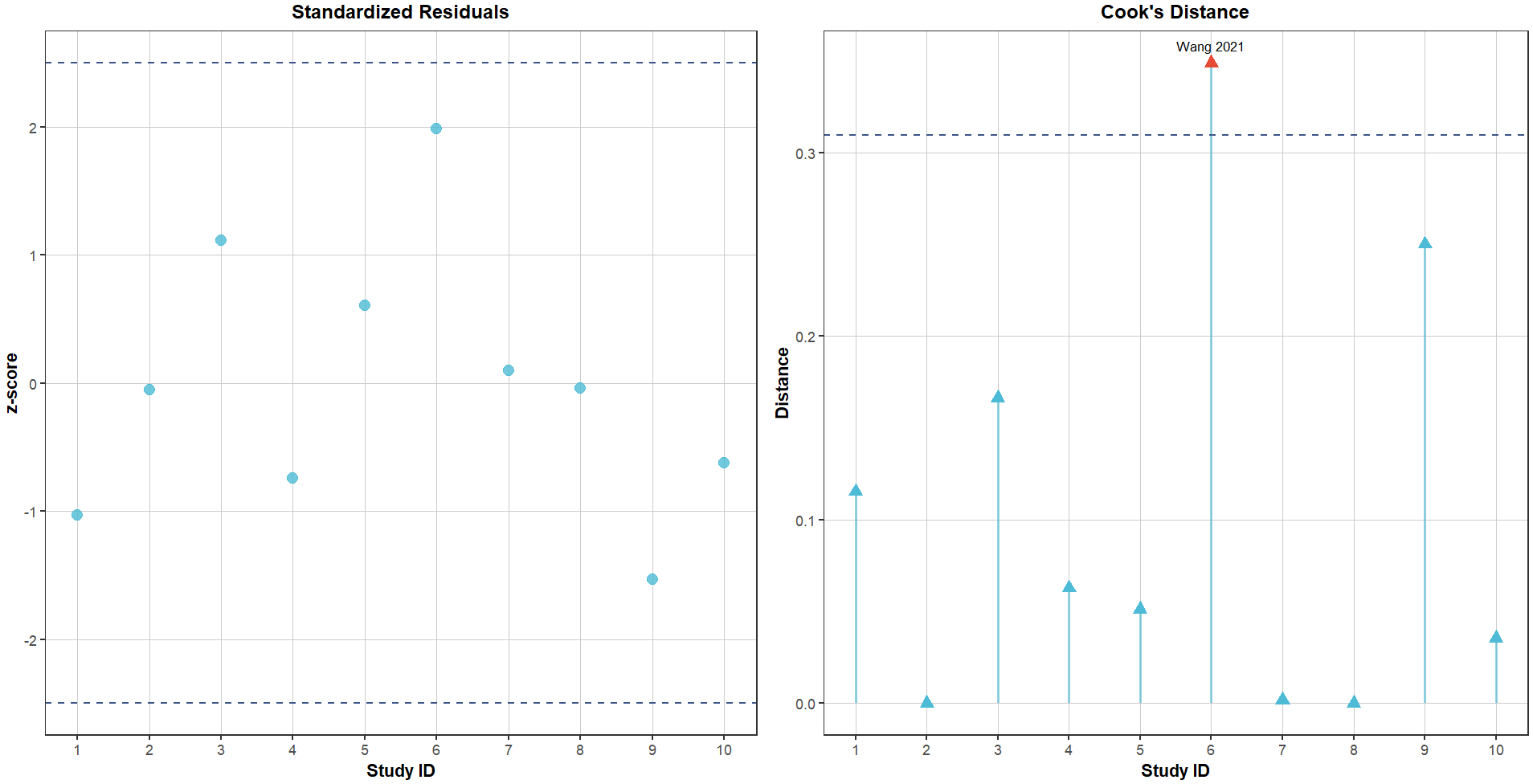


## Influence diagnostics for included studies using standardized residuals and Cook’s distance.


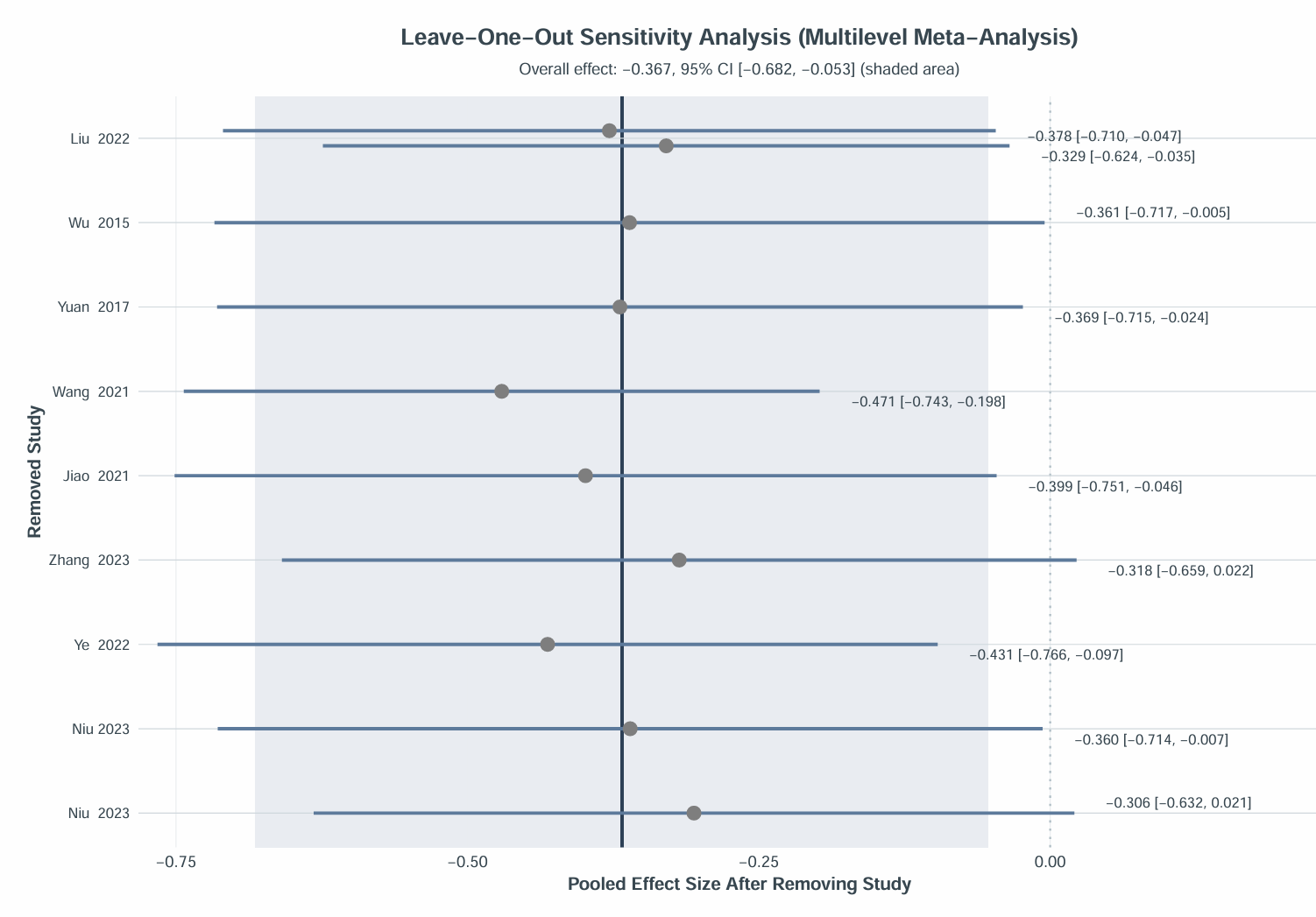


## Figure of the Initial Sensitivity Analysis.


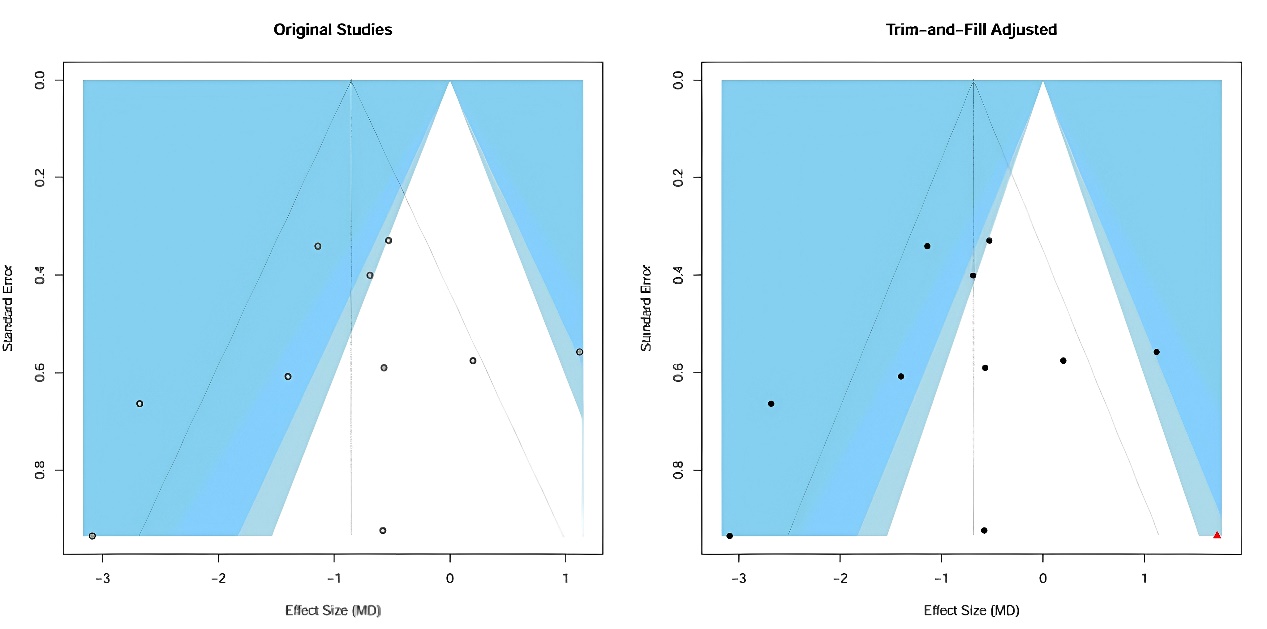


## Trim-and-Fill Funnel Plot.


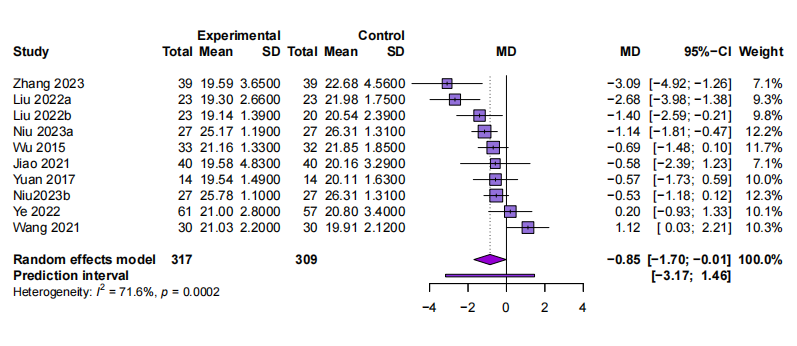


## Forest plot of traditional Chinese mind body training effects on BMI in university students.

## Summary of Findings — Effects of Chinese Traditional Mind-Body Practices on BMI in College Students.

| GRADE Domain | Judgment | Rationale (Strictly Based on the Original Article) |
| --- | --- | --- |
| Risk of Bias | Serious (Downgraded by 1 level) | Among the 8 included studies, there were concerns about the randomization process (D1, Cohen’s κ = 0.47) and deviations from intended interventions (D2, Cohen’s κ = 0.11). No study was rated as high risk, and missing outcome data (D3) was rated as low risk. |
| Inconsistency | Serious (Downgraded by 1 level) | Heterogeneity was significant (I² = 72.2%). Although subgroup analysis identified an optimal intervention scheme, heterogeneity was not fully explained. Variance partitioning showed that between-study heterogeneity accounted for 75.4% of total variability. |
| Indirectness | Not downgraded | The population, intervention, comparator, and outcomes were directly aligned with the research question, with no concerns regarding applicability. |
| Imprecision | Not downgraded | Eight studies involving 746 participants were included. The pooled effect size showed a statistically significant reduction in BMI (MD = −0.77, 95% CI: −1.48 to −0.06, P = 0.034). The confidence interval did not cross the line of no effect, and sensitivity analyses confirmed stable results. |
| Publication Bias | Not downgraded | Egger’s test showed no significant publication bias (P = 0.561), and the result remained stable after trim-and-fill correction, with no changes to the overall pattern of results. |
| Overall quality of evidence | Moderate | Evidence from randomized controlled trials initially started at a high level but was downgraded by two levels due to concerns regarding risk of bias and substantial heterogeneity across studies. No further downgrading was applied for indirectness, imprecision, or publication bias. Therefore, the overall certainty of the evidence was rated as **moderate** according to the GRADE framework. |

# **Resting Heart Rate**


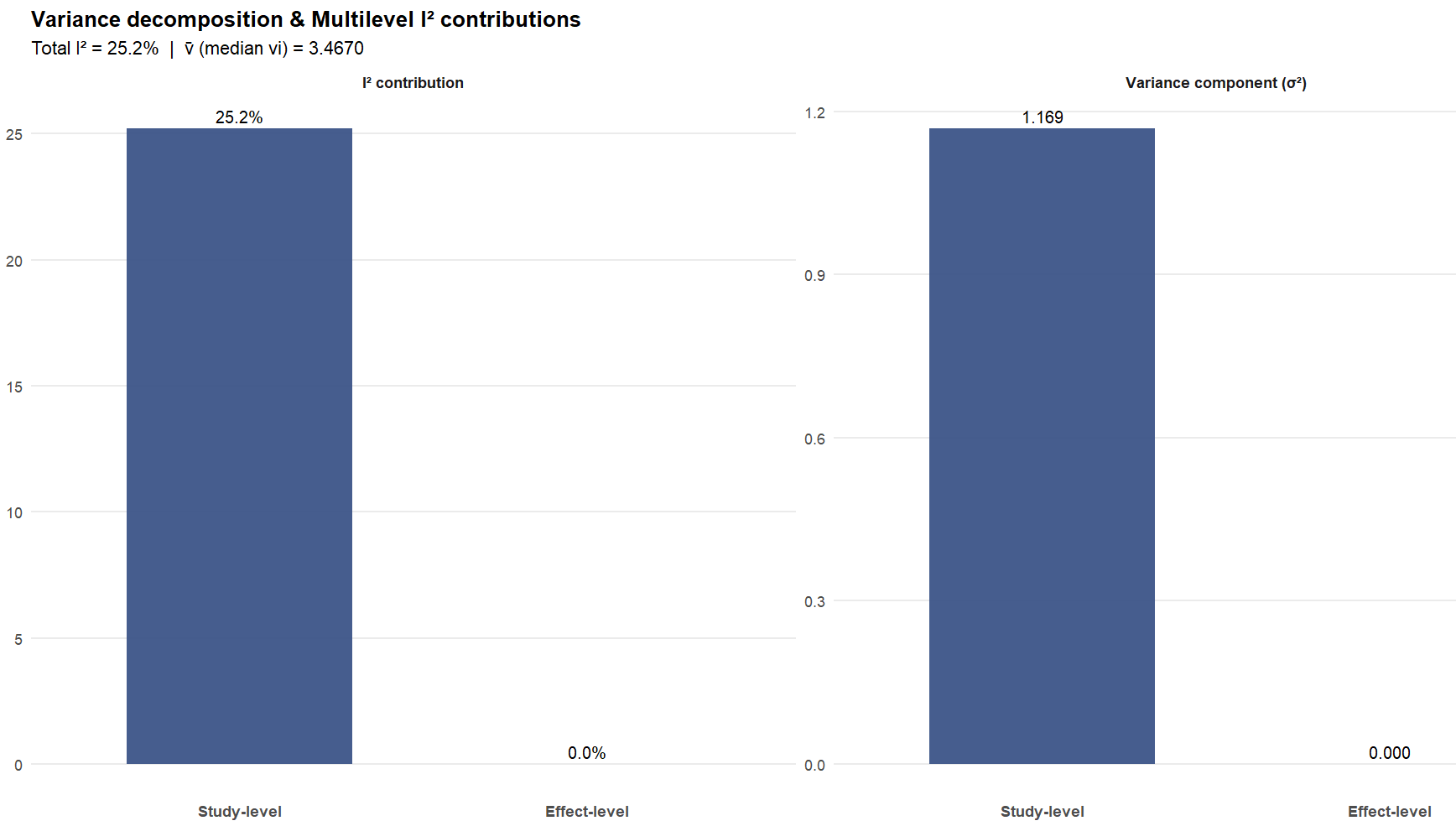


## Variance decomposition and multilevel I².


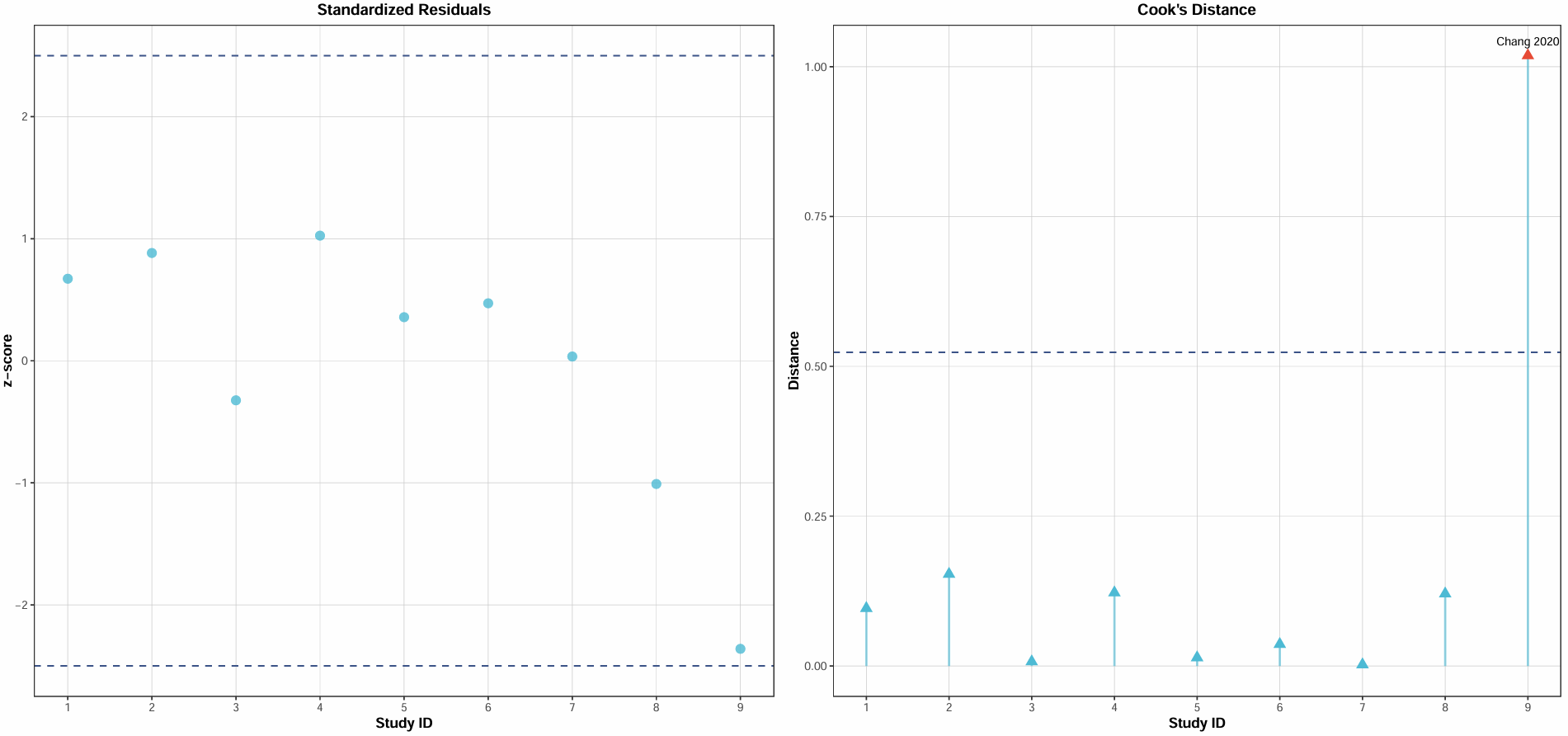


## Influence diagnostics for included studies using standardized residuals and Cook’s distance.


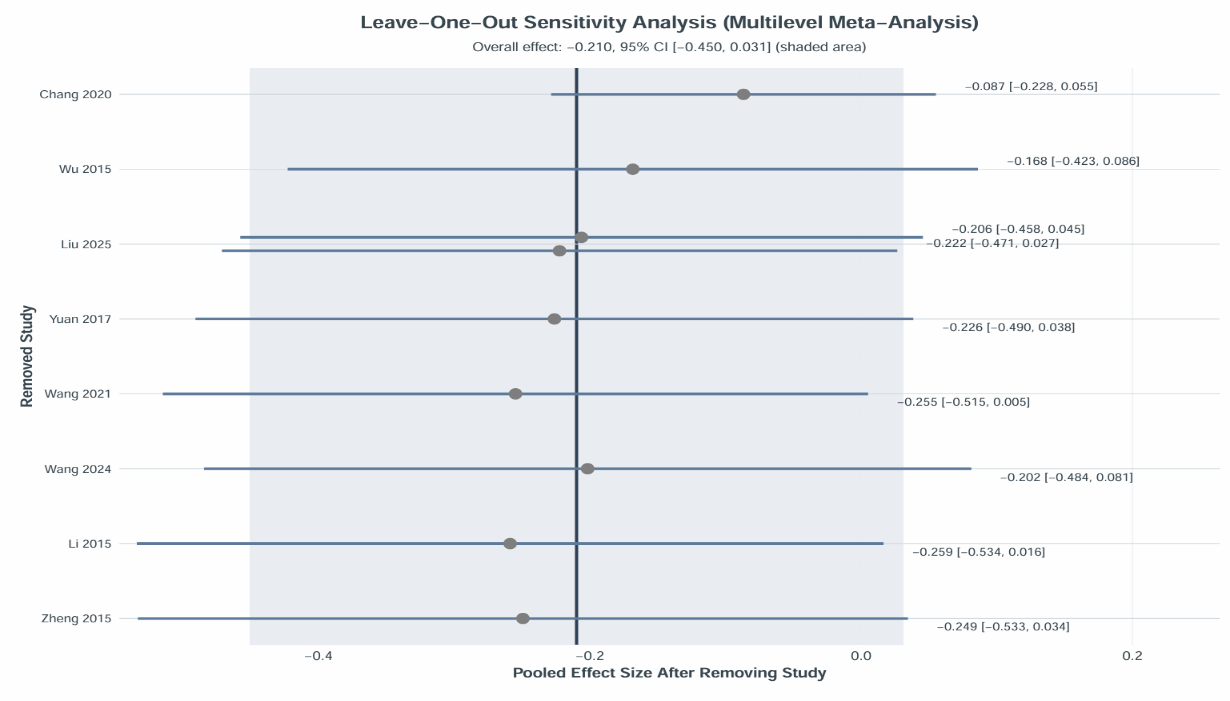


## Figure of the Initial Sensitivity Analysis.


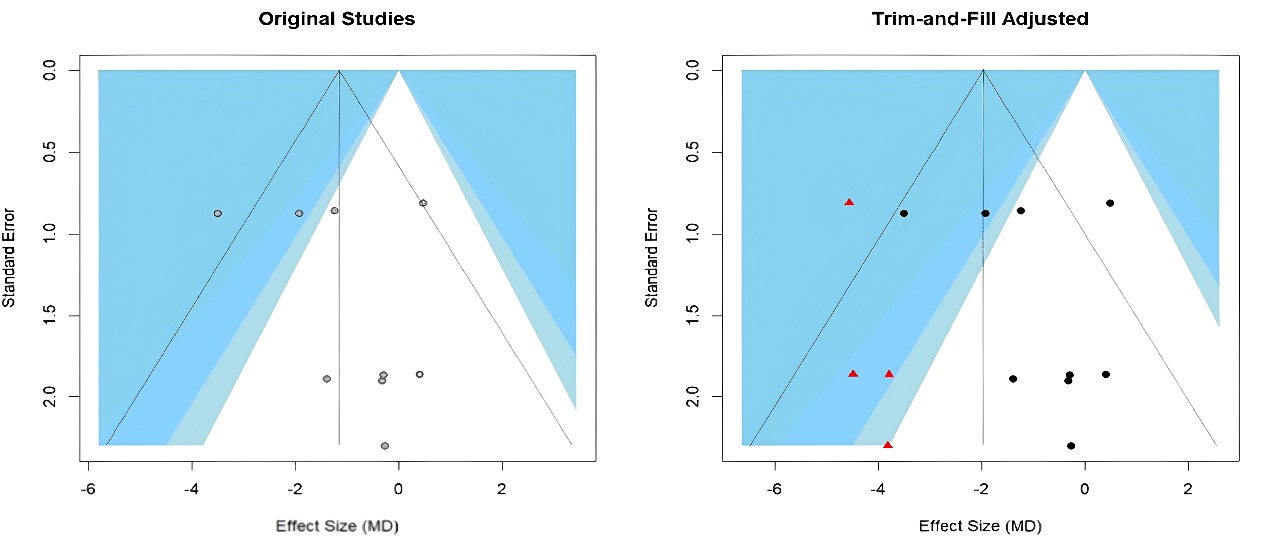


## Trim-and-Fill Funnel Plot.


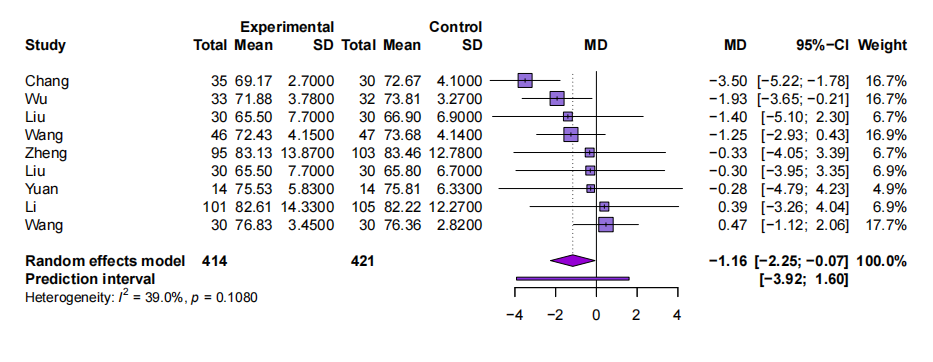


## Forest plot of traditional Chinese mind body training effects on Resting Heart Rate in university students.

## Summary of Findings — Effects of Chinese Traditional Mind-Body Practices on Resting Heart Rate in College Students.

| GRADE Domain | Judgment | Rationale (Strictly Based on the Original Article) |
| --- | --- | --- |
| Risk of Bias | Serious (Downgraded by 1 level) | Among the eight included studies, some concerns were identified in the randomization process (D1, Cohen’s κ ≈ 0.50) and deviations from intended interventions (D2, Cohen’s κ ≈ 0.11). No study was judged as high risk, and missing outcome data (D3) was consistently rated as low risk. |
| Inconsistency | Not downgraded | Statistical heterogeneity was low (I² = 25.2%). Variance partitioning indicated that between-study heterogeneity accounted for nearly all observed variability, while within-study heterogeneity was negligible, suggesting consistent findings across studies. |
| Indirectness | Not downgraded | The population (university students), interventions (traditional Chinese mind-body practices), comparators (no intervention or routine activities), and outcome (resting heart rate) directly matched the research question without concerns regarding applicability. |
| Imprecision | Not downgraded | Eight studies involving 788 participants were included. The pooled analysis showed a statistically significant reduction in resting heart rate (MD = −1.16, 95% CI: −2.29 to −0.04, P = 0.043). The confidence interval did not cross the null effect, and sensitivity analyses confirmed the robustness of the results. |
| Publication Bias | Not downgraded | Because fewer than ten studies were included (k = 8), Egger’s regression test was not performed. However, sensitivity analyses indicated stable results, and no evidence suggested that publication bias materially influenced the findings. |
| Overall quality of evidence | Moderate | Evidence from randomized controlled trials initially started at a high level but was downgraded by one level due to concerns regarding risk of bias in several included studies. No downgrading was applied for inconsistency, indirectness, imprecision, or publication bias. Therefore, the overall certainty of the evidence was rated as **moderate** according to the GRADE framework. |

# **Step Test Index**


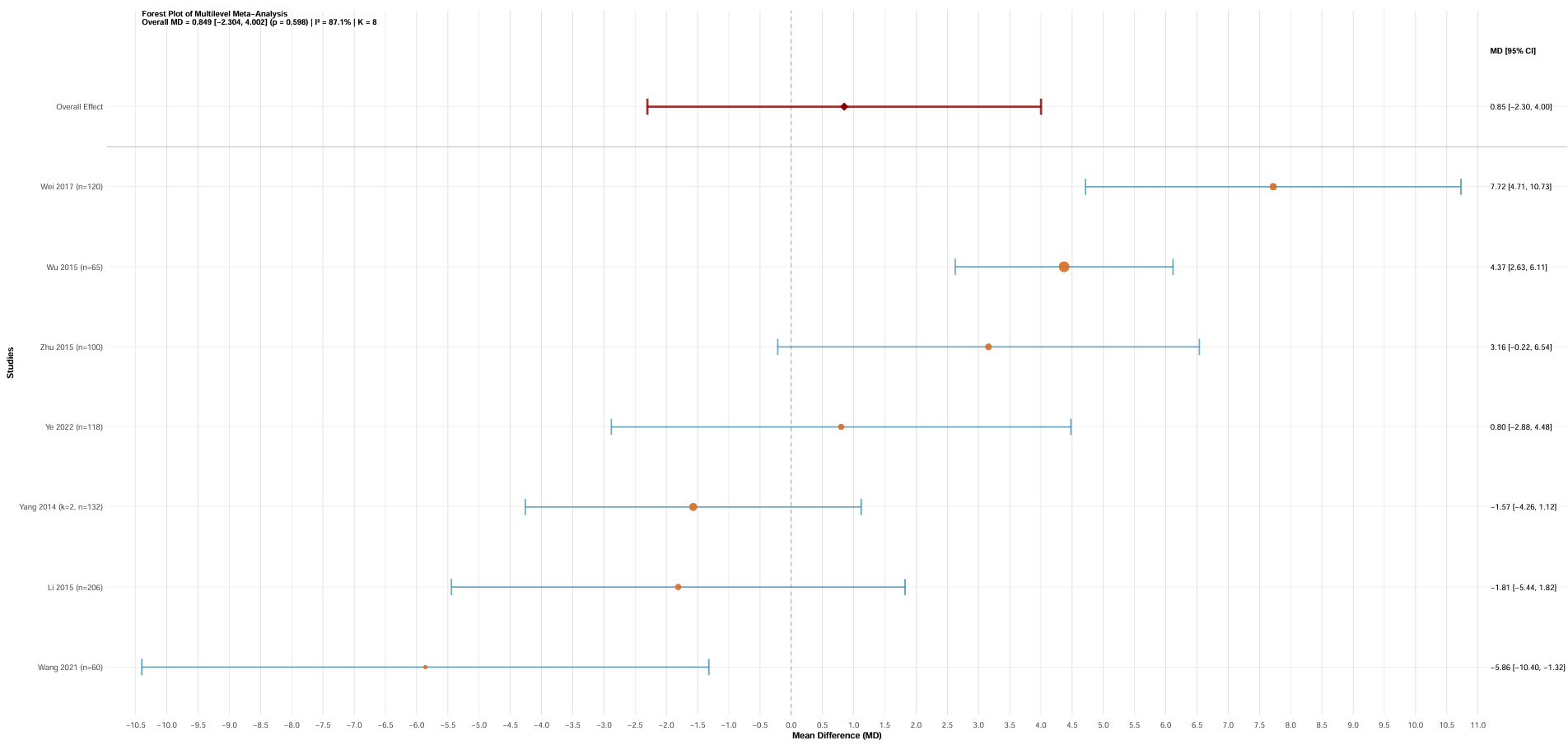


## Forest plot showing the effect of traditional Chinese physical and mental training on the Step Test Index of college students.


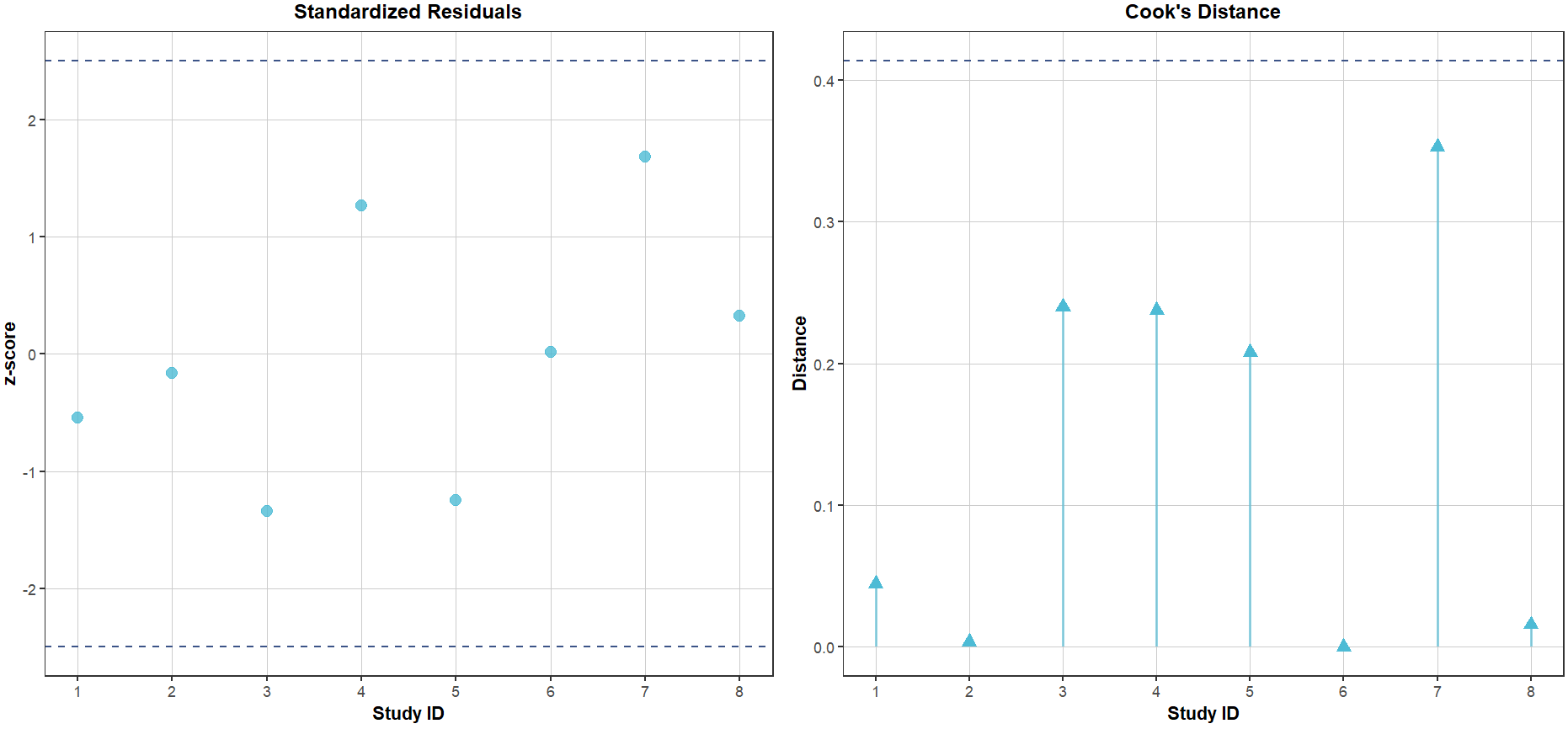


## Influence diagnostics for included studies using standardized residuals and Cook’s distance.


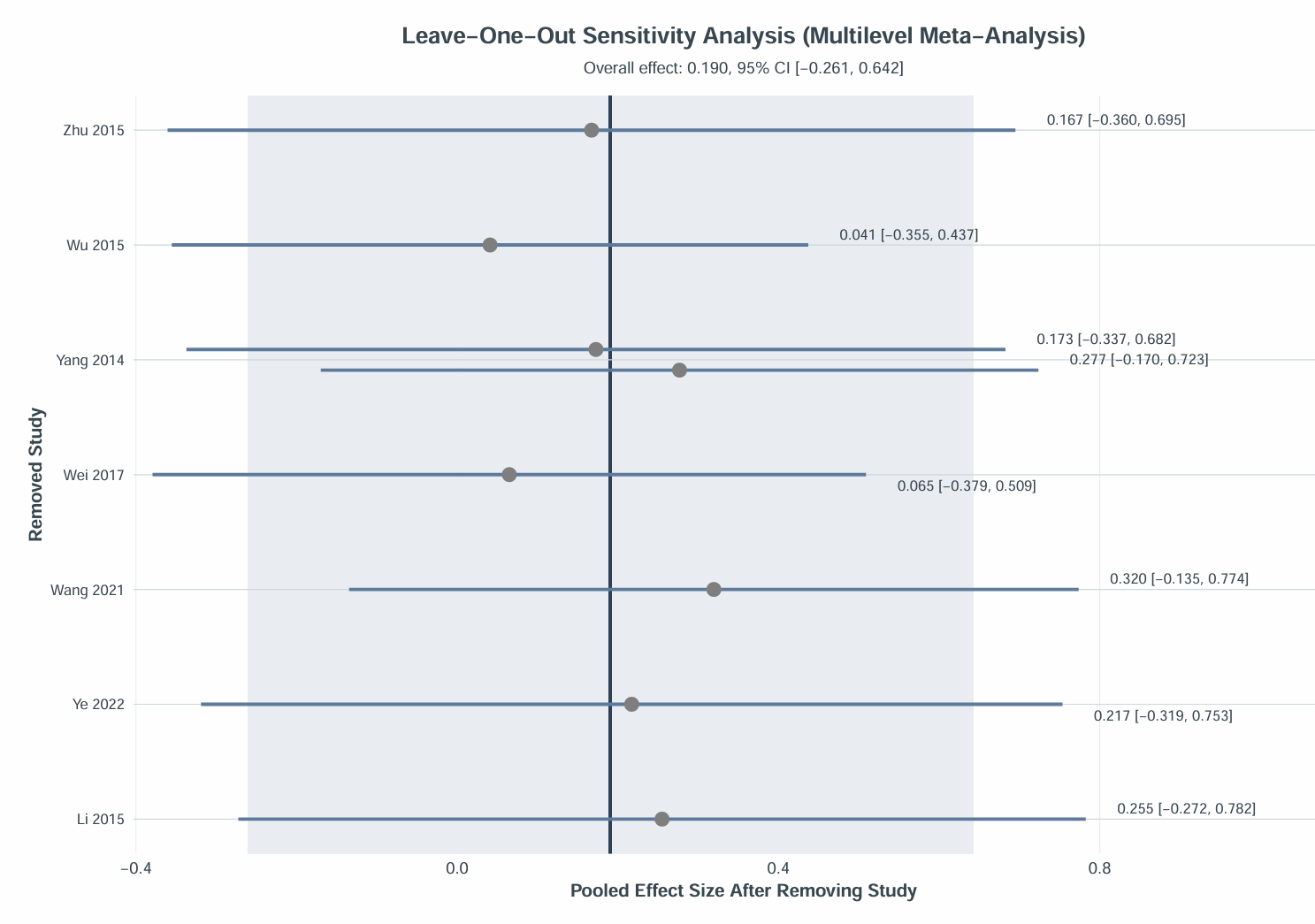


## Figure of the Initial Sensitivity Analysis.

## Summary of Findings — Effects of Chinese Traditional Mind-Body Practices on Step Test Index in College Students.

| GRADE Domain | Judgment | Rationale (Strictly Based on the Original Article) |
| --- | --- | --- |
| Risk of Bias | Serious (Downgraded by 1 level) | Among the seven included studies, several trials presented methodological concerns regarding the randomization process (D1, Cohen’s κ ≈ 0.50) and deviations from intended interventions (D2, Cohen’s κ ≈ 0.11). No study was rated as high risk, while missing outcome data (D3) and selective reporting (D5) were generally assessed as low risk. |
| Inconsistency | Serious (Downgraded by 1 level) | Substantial heterogeneity was observed across studies (I² = 87.1%), indicating considerable variability in effect estimates. Sensitivity and subgroup analyses failed to identify a clear source of heterogeneity, and the inconsistency among studies remained unexplained. |
| Indirectness | Not downgraded | The population (university students), interventions (traditional Chinese mind-body practices), comparators (no intervention or routine activities), and outcome measure (step test index) directly corresponded to the research question, with no concerns regarding indirectness. |
| Imprecision | Serious (Downgraded by 1 level) | The pooled estimate showed no statistically significant effect (MD = 0.85, 95% CI: −2.30 to 4.00, P = 0.598). The confidence interval was wide and crossed the null value, suggesting considerable uncertainty in the magnitude and direction of the effect. |
| Publication Bias | Not downgraded | Because fewer than ten studies were included (k = 7), formal assessment of publication bias using Egger’s regression was not conducted. Influence diagnostics and sensitivity analyses did not identify any outlier studies that materially affected the pooled estimates. |
| Overall quality of evidence | Very Low | Evidence from randomized controlled trials initially started at a high level but was downgraded by three levels due to concerns regarding risk of bias, substantial heterogeneity across studies, and imprecision of the pooled estimates. No further downgrading was applied for indirectness or publication bias. Therefore, the overall certainty of the evidence was rated as **very low** according to the GRADE framework. |

# **Sit-and-Reach Test**


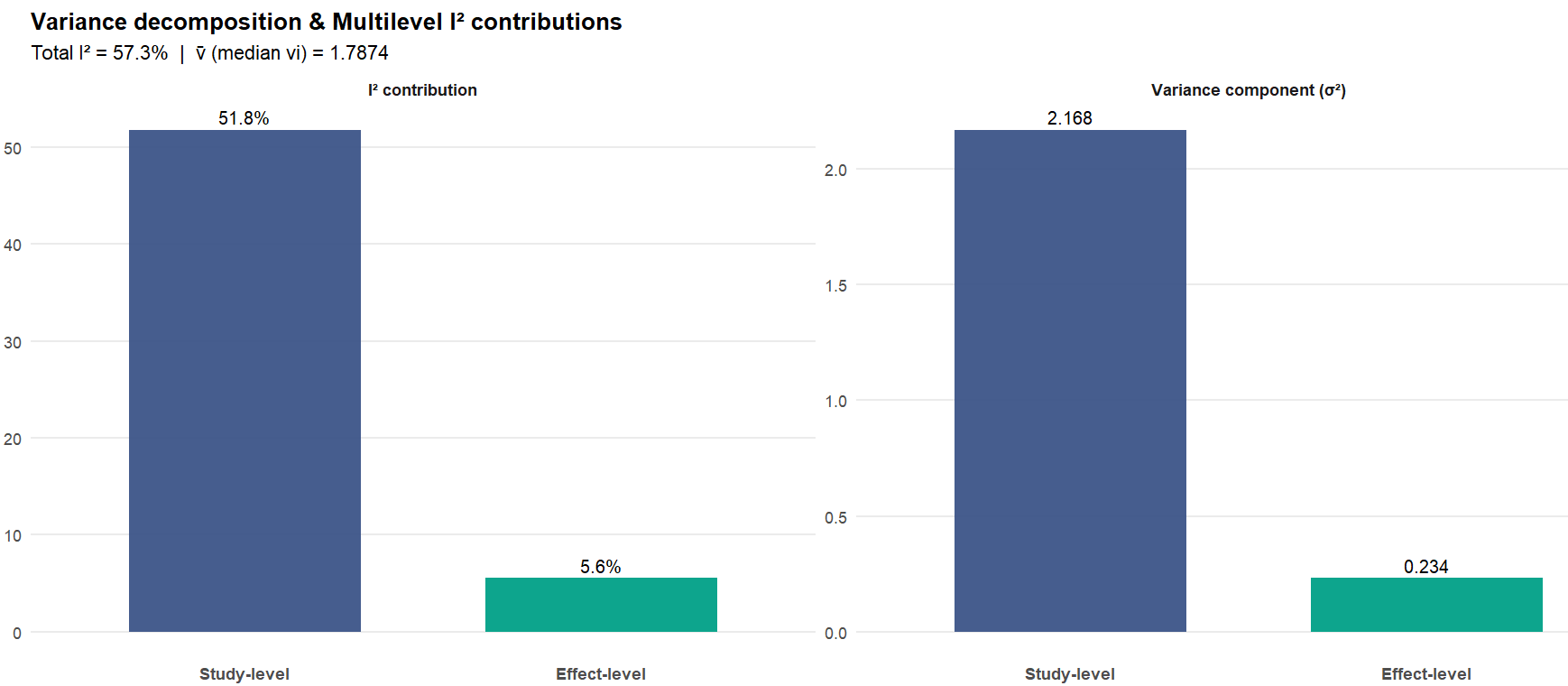


## Variance decomposition and multilevel I².


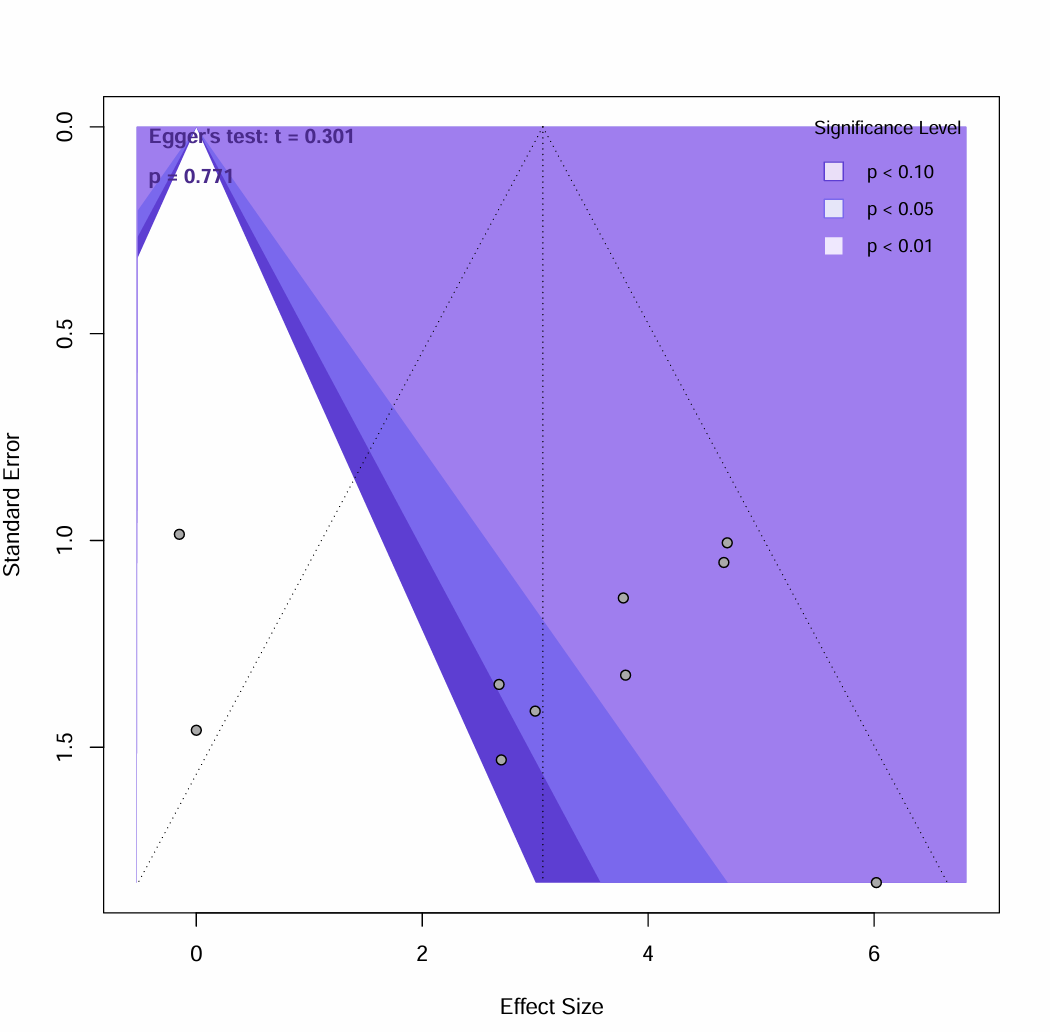


## Egger’s Test Funnel Plot for Publication Bias Assessment.


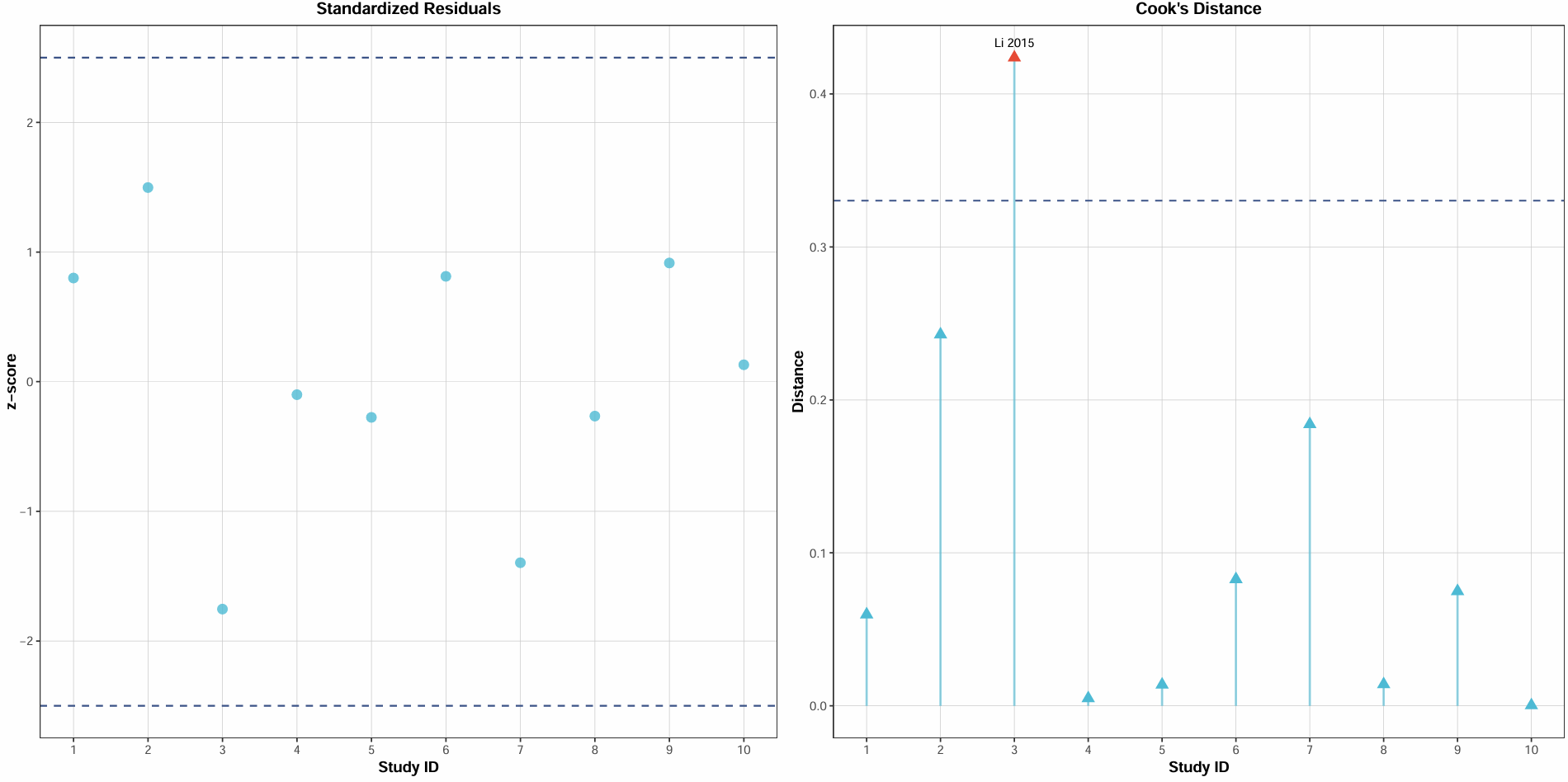
Influence diagnostics for included studies using standardized residuals and Cook’s distance.


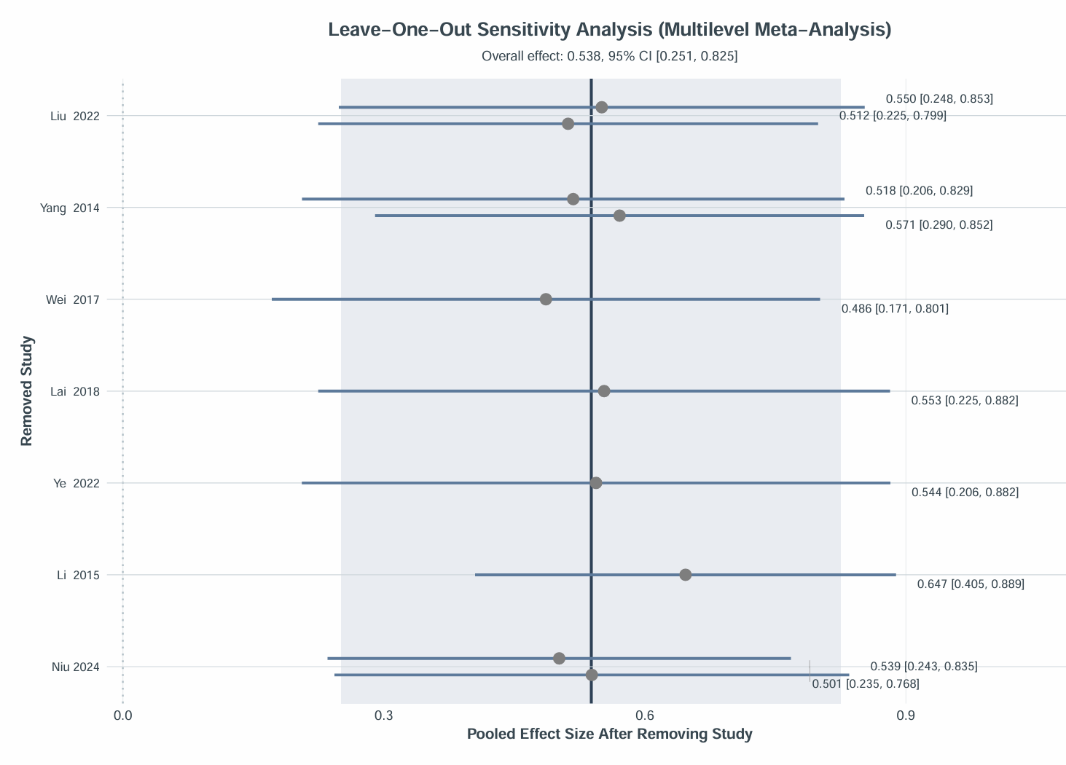


## Figure of the Initial Sensitivity Analysis.


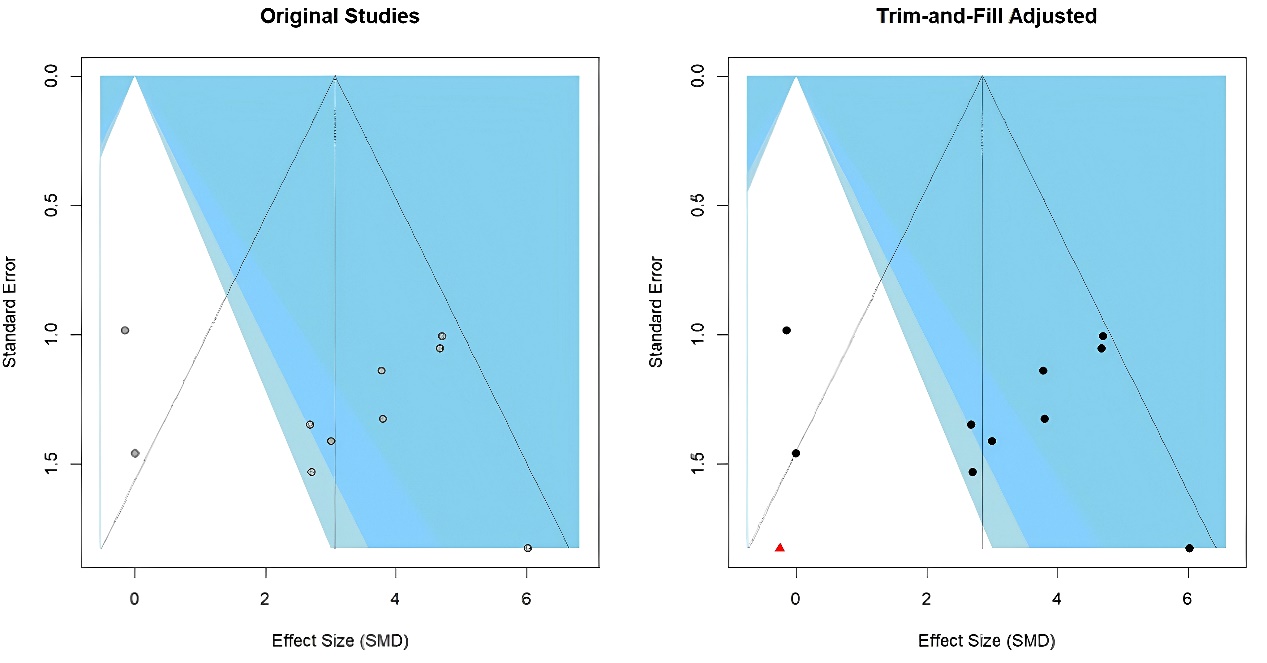


## Trim-and-Fill Funnel Plot.


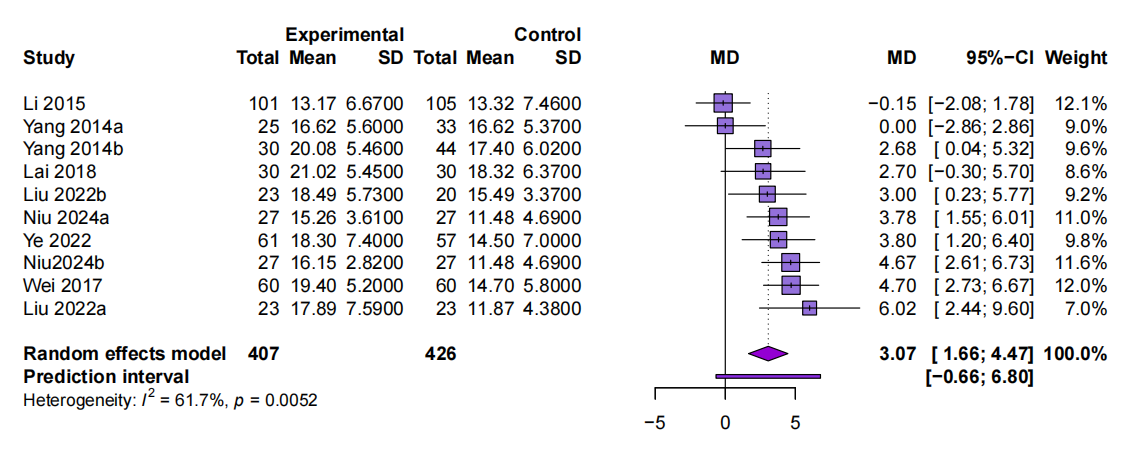


## Forest plot of traditional Chinese mind body training effects on sit-and-reach test in university students.

## Summary of Findings — Effects of Chinese Traditional Mind-Body Practices on sit-and-reach test in College Students.

| GRADE Domain | Judgment | Rationale (Strictly Based on the Original Article) |
| --- | --- | --- |
| Risk of Bias | Serious (Downgraded by 1 level) | Among the 7 included studies, there were concerns about the randomization process (D1, Cohen’s κ = 0.48) and deviations from intended interventions (D2, Cohen’s κ = 0.15). No study was rated as high risk, and missing outcome data (D3) was rated as low risk. |
| Inconsistency | Not downgraded | Heterogeneity was moderate (I² = 57.3%). Variance partitioning showed that between-study heterogeneity accounted for 90.3% of the total variability, while within-study heterogeneity was negligible (9.7%). |
| Indirectness | Not downgraded | The population, intervention, comparator, and outcomes were directly aligned with the research question, with no concerns regarding applicability. |
| Imprecision | Not downgraded | A total of 7 studies (1,082 participants) were included, with a pooled effect size of **MD = 2.99** (95% CI: 1.59 to 4.38, P = 0.000). The confidence interval did not include 0, and the sensitivity analysis showed stable results. |
| Publication Bias | Not downgraded | Egger’s test showed no significant publication bias (P = 0.771), and the funnel plot was symmetric. |
| Overall quality of evidence | Moderate | Evidence from randomized controlled trials started at a high level but was downgraded by one level due to concerns about the risk of bias in some studies. No further downgrading was applied for inconsistency, imprecision, or publication bias. Therefore, the overall certainty of the evidence was rated as **moderate** according to the GRADE framework. |

# **Handgrip Strength**


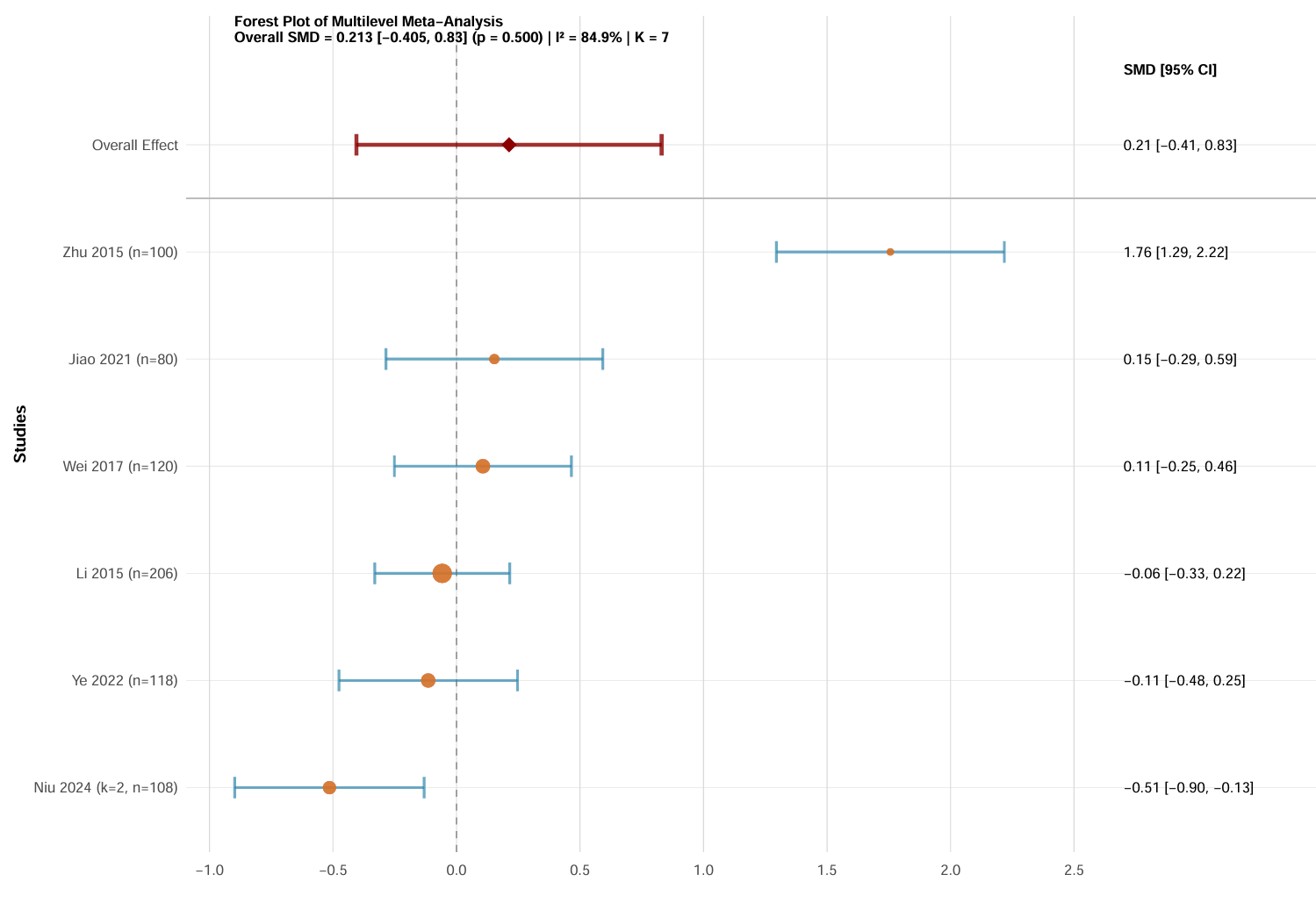


## Forest plot showing the effect of traditional Chinese physical and mental training on the Handgrip Strength of college students.


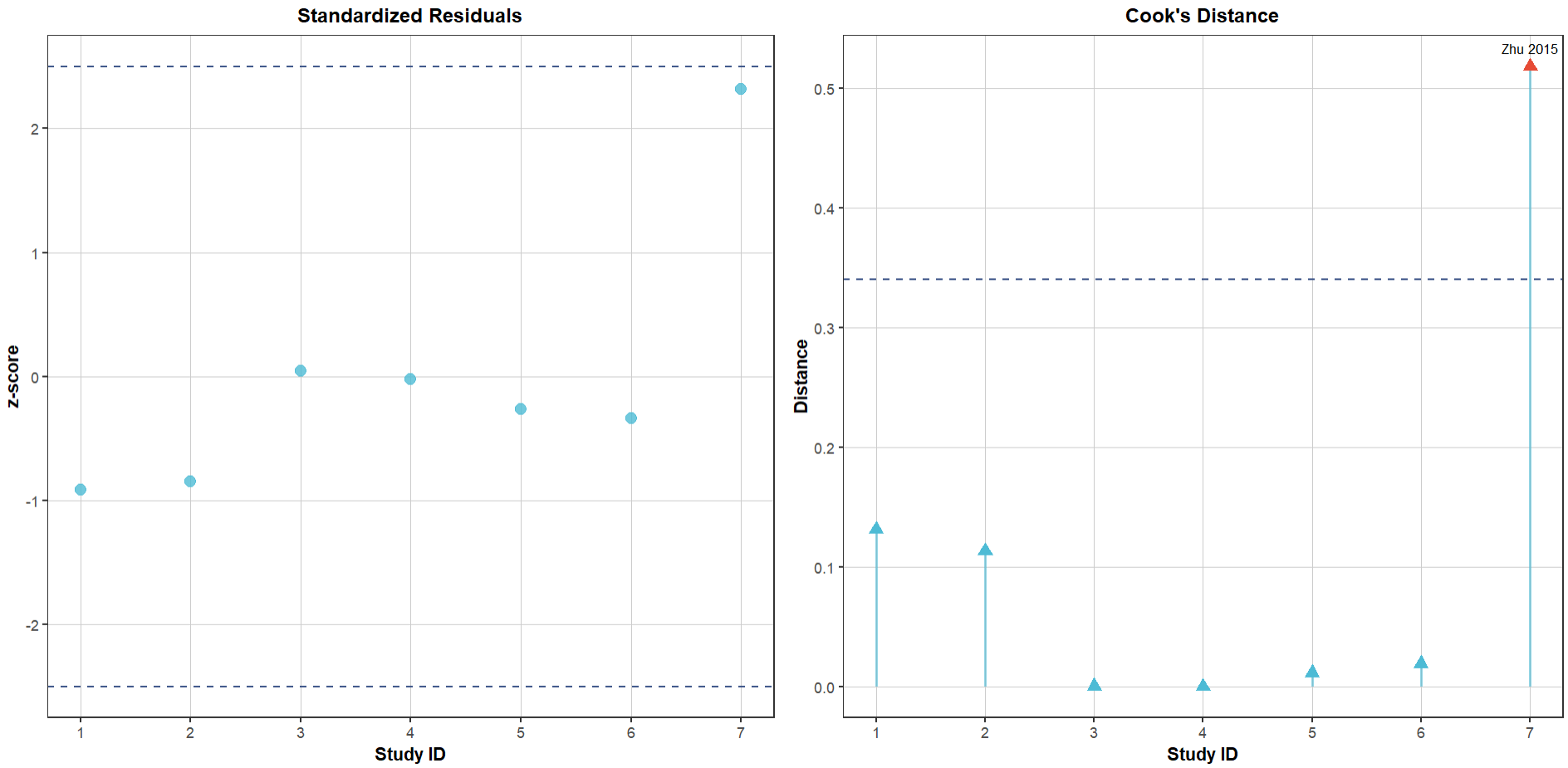


## Influence diagnostics for included studies using standardized residuals and Cook’s distance.


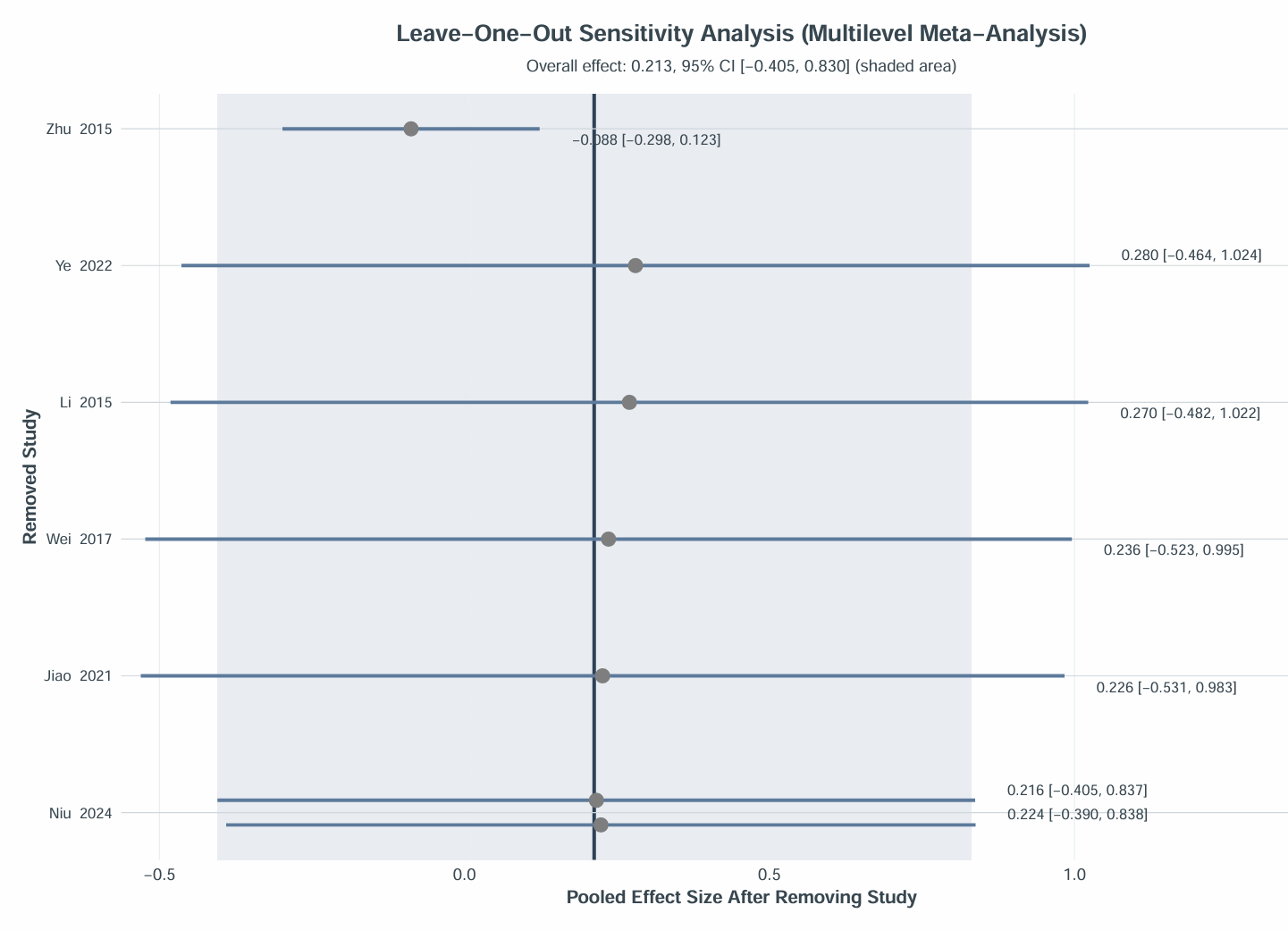


## Figure of the Initial Sensitivity Analysis.

## Summary of Findings — Effects of Chinese Traditional Mind-Body Practices on Handgrip Strength in College Students.

| GRADE Domain | Judgment | Rationale (Strictly Based on the Original Article) |
| --- | --- | --- |
| Risk of Bias | Serious (Downgraded by 1 level) | Among the 6 included studies, there were concerns about the randomization process (D1, Cohen’s κ = 0.43) and deviations from intended interventions (D2, Cohen’s κ = 0.09). No study was rated as high risk, and missing outcome data (D3) was rated as low risk. |
| Inconsistency | Serious (Downgraded by 1 level) | Heterogeneity was extremely high (I² = 84.9%). Subgroup analysis failed to identify the optimal intervention scheme, and heterogeneity could not be explained. |
| Indirectness | Not downgraded | The population, intervention, comparator, and outcomes were directly aligned with the research question, with no concerns regarding applicability. |
| Imprecision | Serious (Downgraded by 1 level) | The pooled effect size was SMD = 0.21 (95%CI: -0.41 to 0.83, P = 0.500), with a confidence interval including 0, failing to reach statistical significance. |
| Publication Bias | Not downgraded | The number of included studies was small (k=6<10), with no outlier studies, and the core conclusion was not disturbed. |
| Overall quality of evidence | Very Low | Evidence from randomized controlled trials started at a high level but was downgraded by three levels due to concerns about risk of bias, extremely high heterogeneity, and imprecision in the effect estimate. No further downgrading was applied for indirectness or publication bias. Therefore, the overall certainty of the evidence was rated as **very low** according to the GRADE framework. |

# **Standing Long Jump**


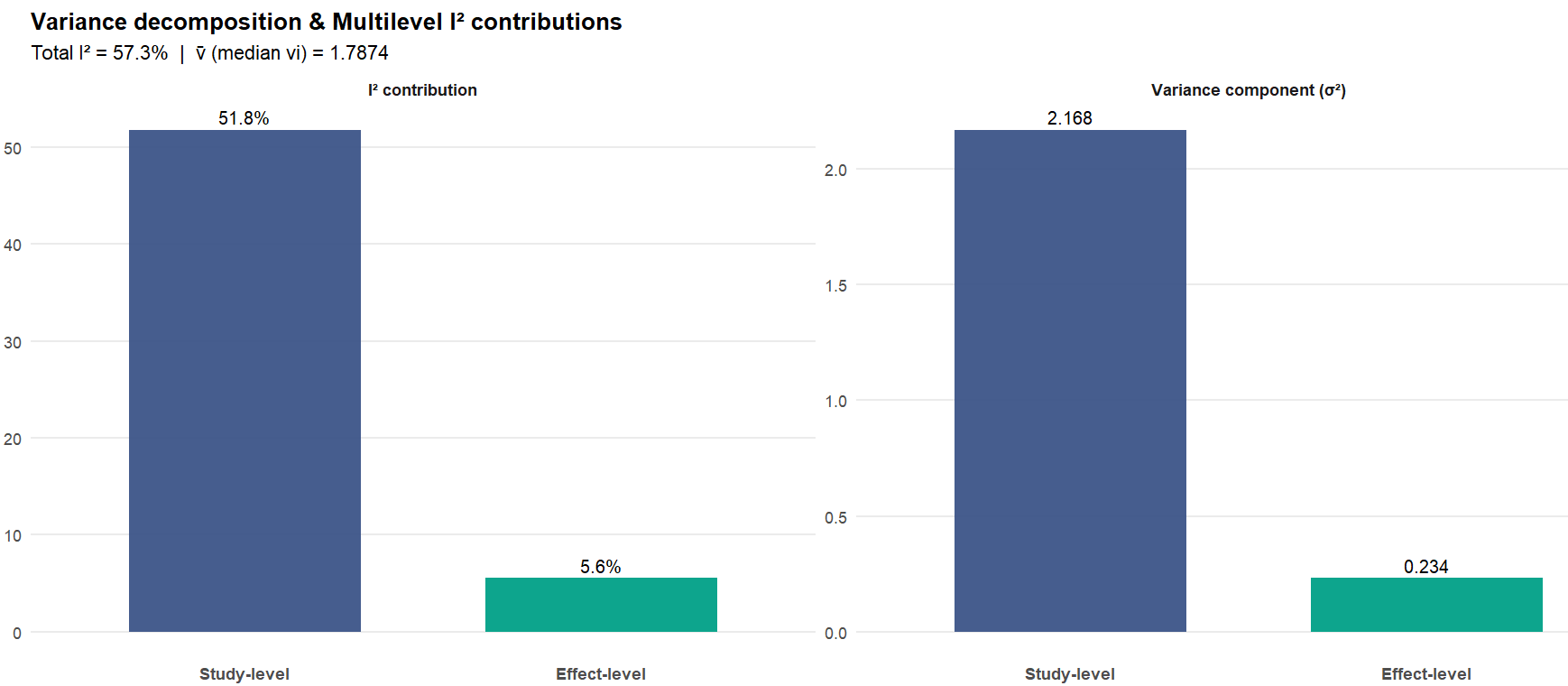


## Variance decomposition and multilevel I².


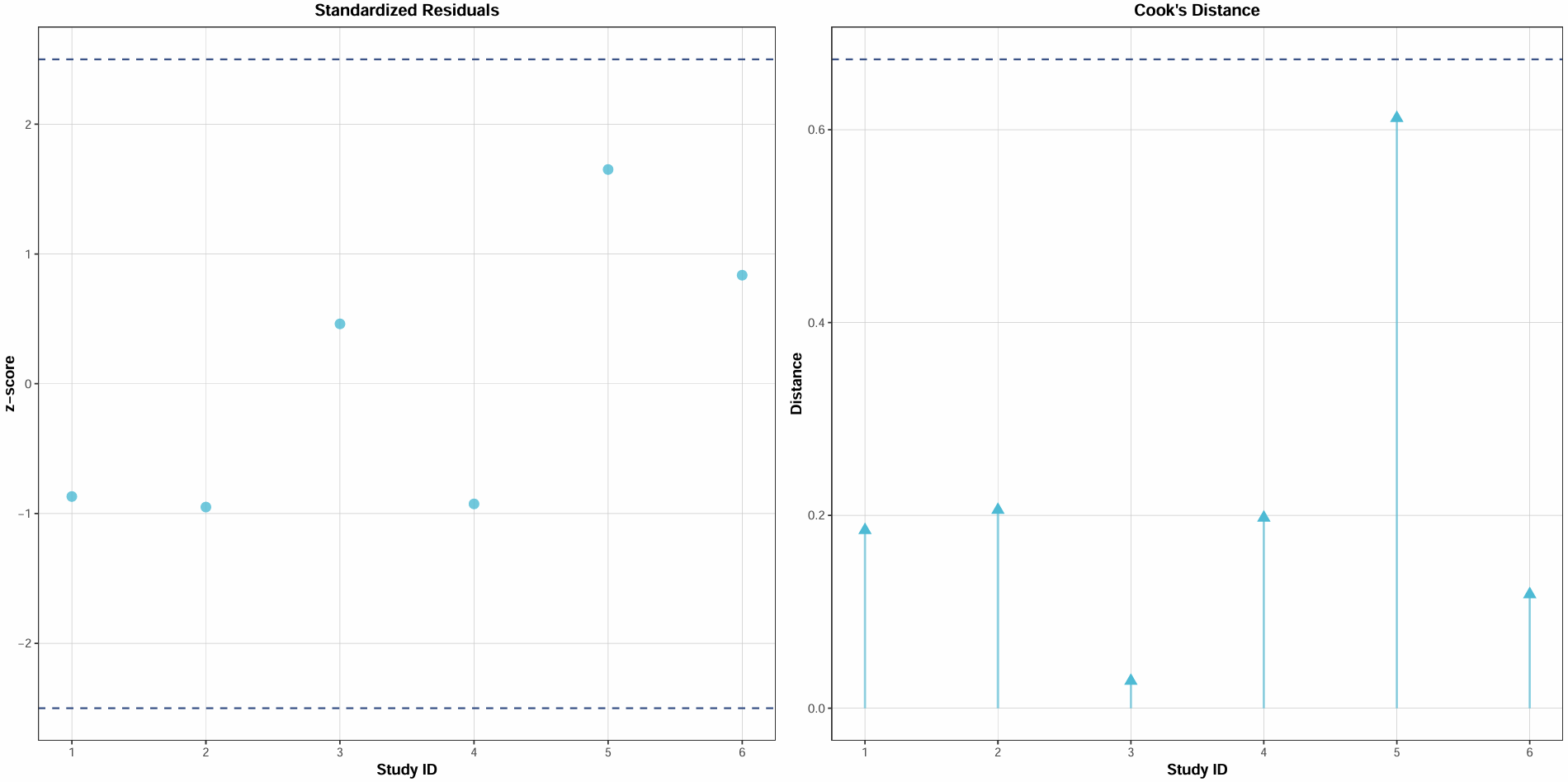


## Influence diagnostics for included studies using standardized residuals and Cook’s distance.


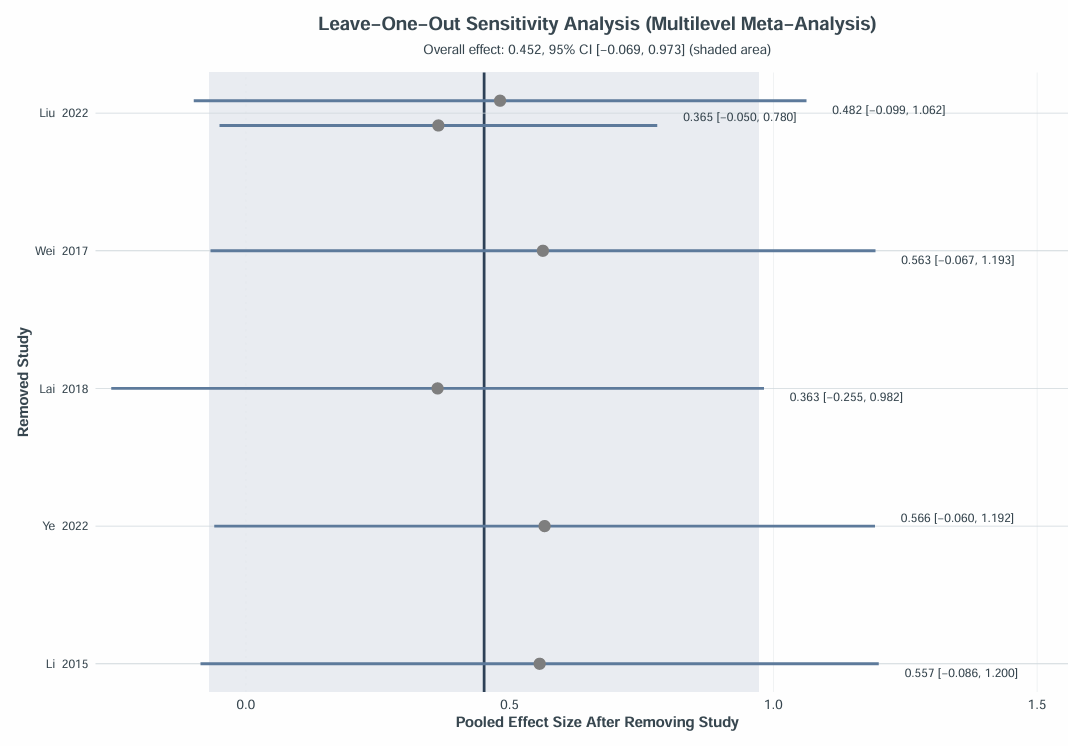


## Figure of the Initial Sensitivity Analysis.


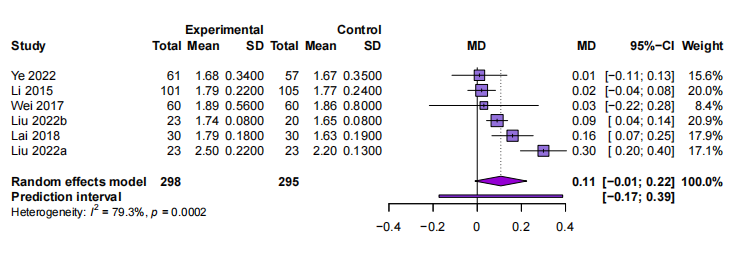


## Forest plot of traditional Chinese mind body training effects on Standing Long Jump in university students.

## Summary of Findings — Effects of Chinese Traditional Mind-Body Practices on Standing Long Jump in College Students.

| GRADE Domain | Judgment | Rationale (Strictly Based on the Original Article) |
| --- | --- | --- |
| Risk of Bias | Serious (Downgraded by 1 level) | Among the 5 included studies, there were concerns about the randomization process (D1, Cohen’s κ = 0.45) and deviations from intended interventions (D2, Cohen’s κ = 0.10). No study was rated as high risk, and missing outcome data (D3) was rated as low risk. |
| Inconsistency | Serious (Downgraded by 1 level) | The heterogeneity was significant (I² = 79.1%), indicating considerable variability between studies. While subgroup analysis suggested an optimal intervention scheme, the source of heterogeneity was not fully explained, leading to a **1-level downgrade**. |
| Indirectness | Not downgraded | The population (university students), intervention (traditional Chinese mind-body practices), comparators (no intervention or alternative exercise), and outcomes (standing long jump) were directly aligned with the research question, indicating no concerns regarding applicability. |
| Imprecision | Not downgraded | Five studies involving 788 participants were included. The pooled effect size was MD = 0.11 (95% CI: 0.02 to 0.20, P = 0.021). The confidence interval did not include 0, and the result was statistically significant, with stable findings from sensitivity analysis. |
| Publication Bias | Not downgraded | The number of included studies was small (k = 5 < 10). No outlier studies were identified, and the core conclusion was not disturbed by potential publication bias. |
| Overall quality of evidence | Low | Evidence from randomized controlled trials initially started at a high level but was downgraded by two levels due to concerns about risk of bias and significant heterogeneity. No further downgrading was applied for indirectness, imprecision, or publication bias. Therefore, the overall certainty of the evidence was rated as **low** according to the GRADE framework. |

# **50-Meter Sprint**


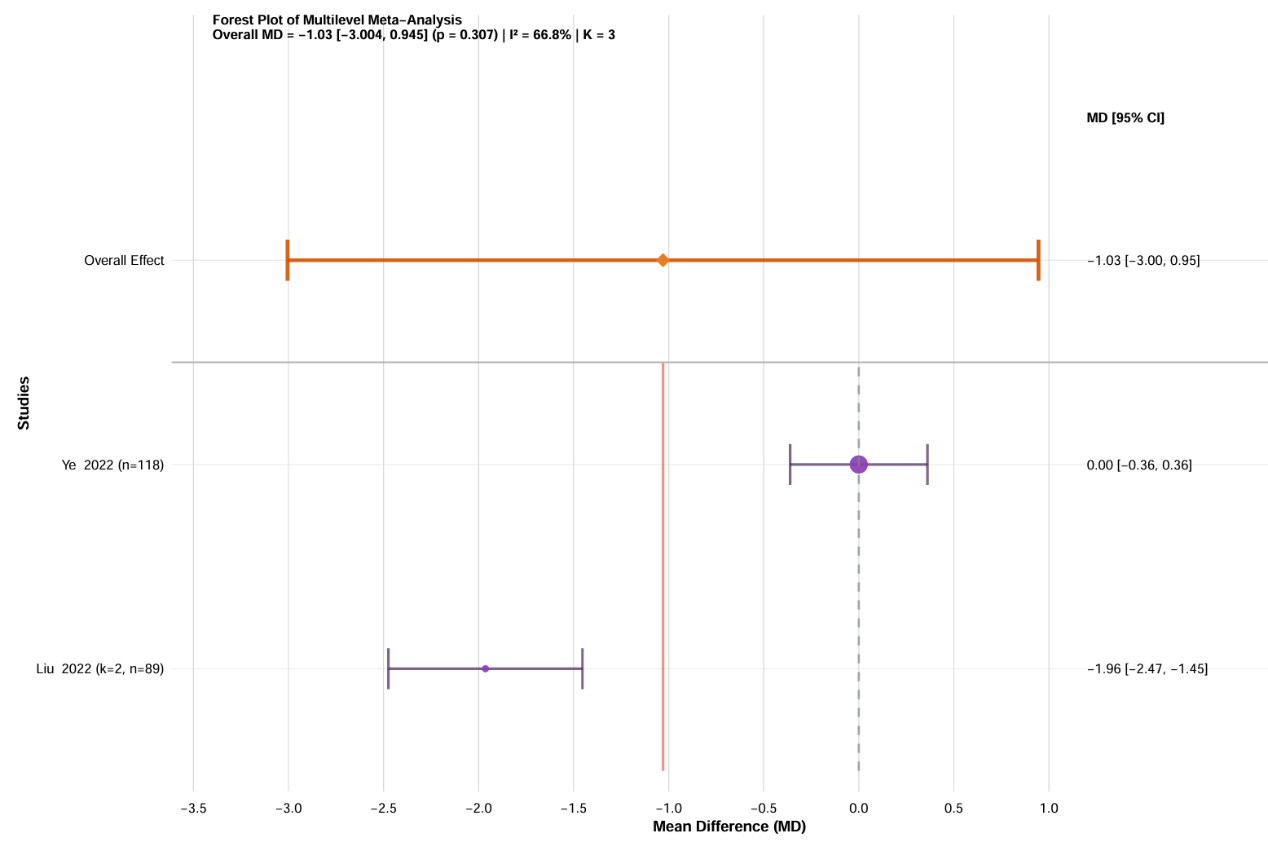


## Forest plot showing the effect of traditional Chinese physical and mental training on the 50-Meter Sprint of college students.


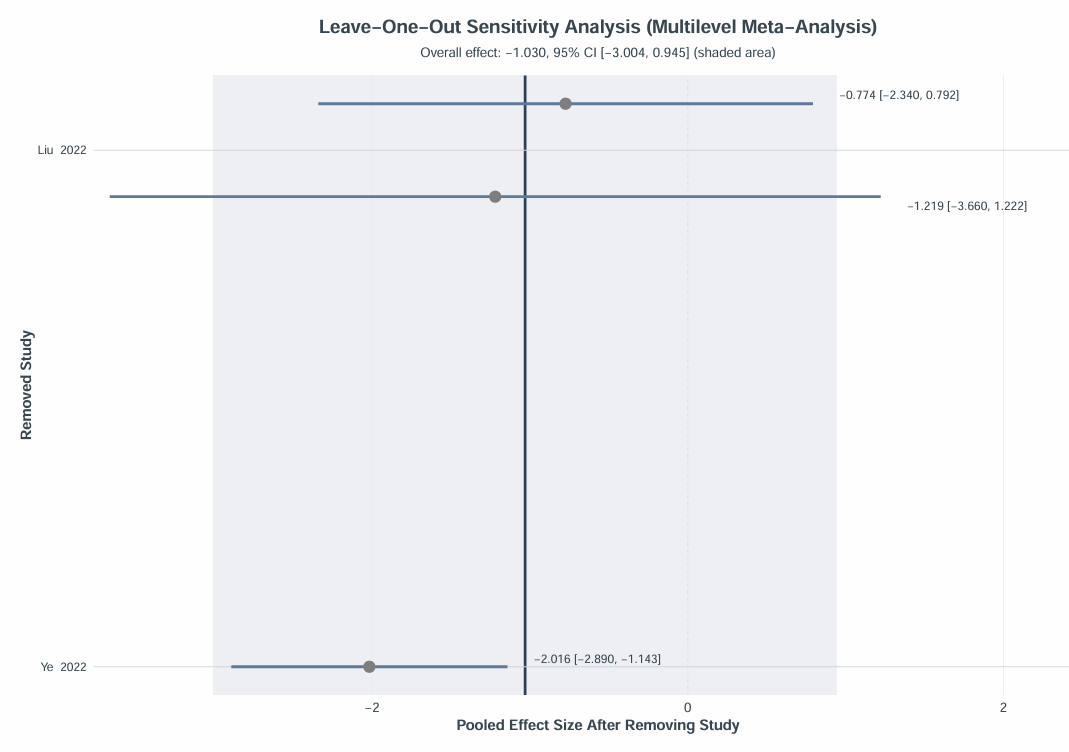


## Figure of the Initial Sensitivity Analysis.

## Summary of Findings — Effects of Chinese Traditional Mind-Body Practices on 50-Meter Sprint in College Students.

| GRADE Domain | Judgment | Rationale (Strictly Based on the Original Article) |
| --- | --- | --- |
| Risk of Bias | Serious (Downgraded by 1 level) | Among the 2 included studies, there were concerns about the randomization process (D1, Cohen’s κ = 0.40) and deviations from intended interventions (D2, Cohen’s κ = 0.07). No study was rated as high risk, and missing outcome data (D3) was rated as low risk. |
| Inconsistency | Not downgraded | Heterogeneity was moderate to high (I² = 66.8%). Although the effect direction remained consistent, sensitivity analyses revealed substantial variation in estimates (MD between −0.77 and −2.02), indicating weak evidence. |
| Indirectness | Not downgraded | The population (university students), intervention (traditional Chinese mind-body practices), comparators (no intervention or alternative exercise), and outcome (50-meter sprint performance) directly aligned with the research question, with no concerns regarding applicability. |
| Imprecision | Serious (Downgraded by 1 level) | Only two studies involving 386 participants were included. The pooled effect size was MD = −1.03 (95% CI: −3.00 to 0.95, P = 0.307). The confidence interval included 0, and the sensitivity analysis showed substantial variation in the results, suggesting weak evidence strength. |
| Publication Bias | Not downgraded | The number of included studies was small (k = 2 < 10), and no significant publication bias was identified, as indicated by the symmetric funnel plot |
| Overall quality of evidence | Very Low | Evidence from randomized controlled trials initially started at a high level but was downgraded by three levels due to concerns regarding risk of bias, significant heterogeneity, and imprecision in the pooled estimates. No further downgrading was applied for indirectness or publication bias. Therefore, the overall certainty of the evidence was rated as **very low** according to the GRADE framework. |

# **Pull-ups/sit-ups**


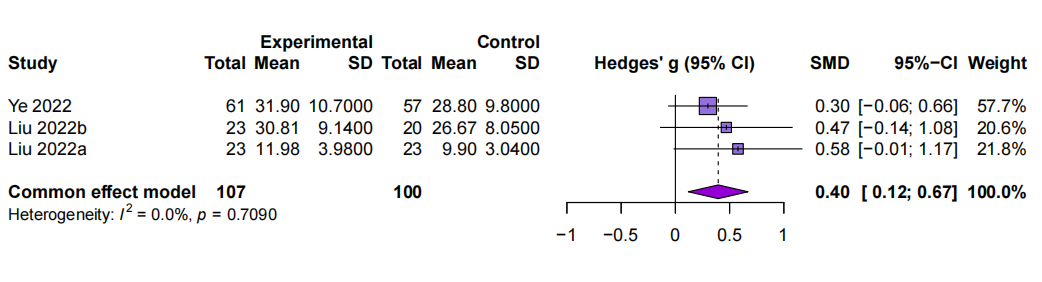


## Forest plot of traditional Chinese mind body training effects on Pull-ups/Sit-ups in university students.

## Summary of Findings — Effects of Chinese Traditional Mind-Body Practices on Pull-ups/Sit-ups in College Students.

| GRADE Domain | Judgment | Rationale (Strictly Based on the Original Article) |
| --- | --- | --- |
| Risk of Bias | Serious (Downgraded by 1 level) | Among the two included studies, there were concerns regarding the randomization process (D1, Cohen’s κ = 0.42) and deviations from intended interventions (D2, Cohen’s κ = 0.08). No study was rated as high risk, and missing outcome data (D3) was rated as low risk. |
| Inconsistency | No serious inconsistency (Not downgraded) | Heterogeneity was low (I² < 50%), and the pooled effect size was stable across studies, indicating that the results were consistent and not significantly influenced by study-level differences. |
| Indirectness | Not downgraded | The population (university students), intervention (traditional Chinese mind-body practices), comparator (no intervention or alternative exercises), and outcome measure (Single-Leg Stance Test) were directly aligned with the research question, with no concerns regarding applicability. |
| Imprecision | Serious (Downgraded by 1 level) | Only two studies involving 386 participants were included. The sample size was small, and the evidence strength was limited, leading to considerable uncertainty in the pooled effect estimate. |
| Publication Bias | Not downgraded | The number of included studies was small (k = 2 < 10), but no obvious publication bias was detected, as the funnel plot showed symmetry, suggesting no major influence from missing data. |
| Overall quality of evidence | Low | Evidence from randomized controlled trials initially started at a high level but was downgraded by two levels due to concerns about risk of bias and imprecision in the pooled estimates. No further downgrading was applied for inconsistency, indirectness, or publication bias. Therefore, the overall certainty of the evidence was rated as **low** according to the GRADE framework. |

# **SLST**


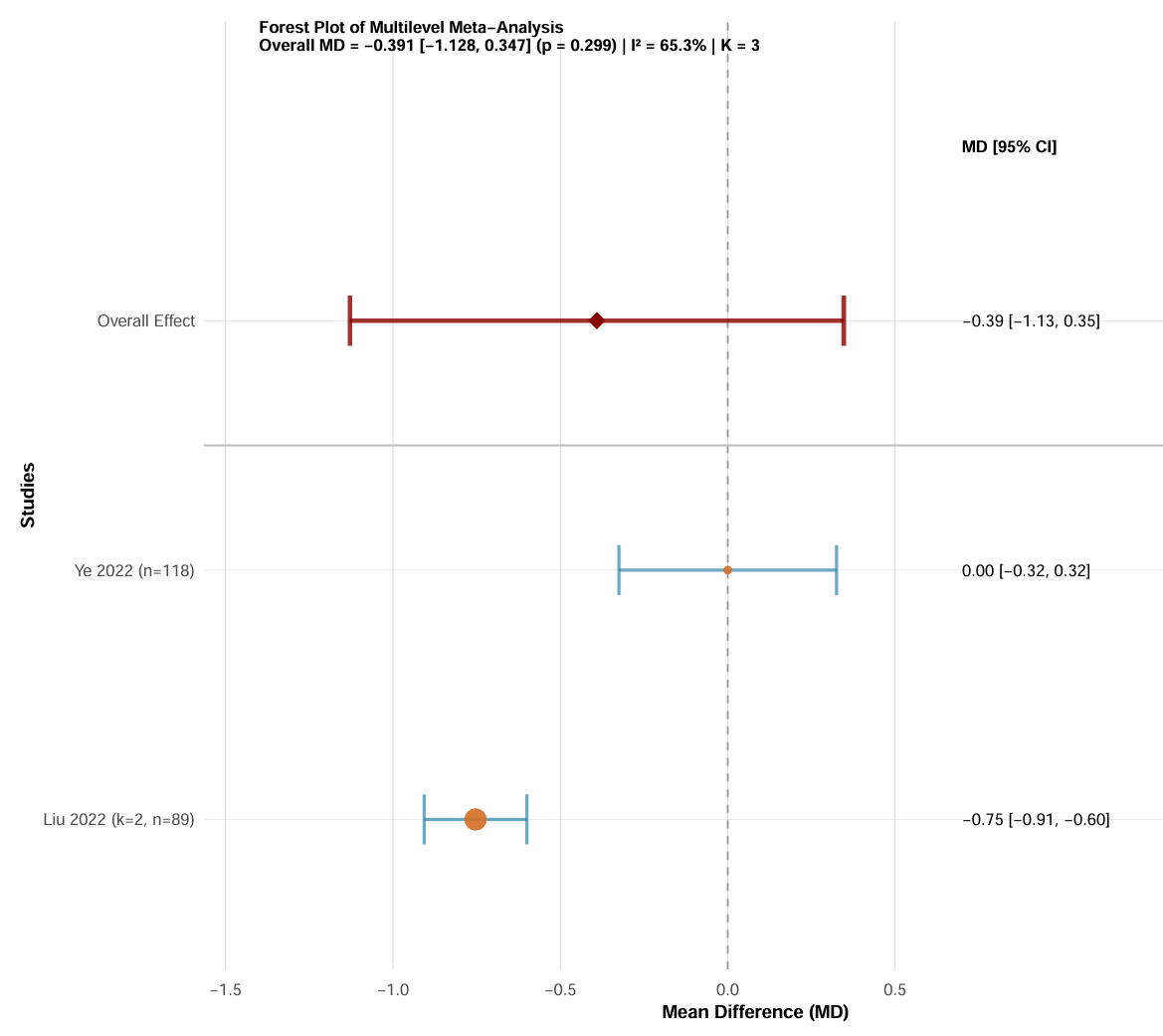


## Forest plot showing the effect of traditional Chinese physical and mental training on the SLST of college students.

## Summary of Findings — Effects of Chinese Traditional Mind-Body Practices on SLST in College Students.

| GRADE Domain | Judgment | Rationale (Strictly Based on the Original Article) |
| --- | --- | --- |
| Risk of Bias | Serious (Downgraded by 1 level) | Among the 2 included studies, there were concerns about the randomization process (D1, Cohen’s κ = 0.41) and deviations from intended interventions (D2, Cohen’s κ = 0.09). No study was rated as high risk, and missing outcome data (D3) was rated as low risk. |
| Inconsistency | Serious (Downgraded by 1 level) | Heterogeneity was moderate to high (I² = 65.3%). Subgroup analysis failed to identify significant differences, and the heterogeneity could not be fully explained, indicating substantial variation between studies. |
| Indirectness | Not downgraded | The population (university students), intervention (traditional Chinese mind-body practices), comparator (no intervention or alternative exercise), and outcomes (Single-Leg Stance Test) were directly aligned with the research question, with no concerns regarding applicability. |
| Imprecision | Serious (Downgraded by 1 level) | Only two studies involving 140 participants were included. The pooled effect size was MD = −0.39 (95% CI: −1.13 to 0.35, P = 0.299), and the confidence interval included 0, failing to reach statistical significance. This indicates substantial uncertainty in the effect estimate. |
| Publication Bias | Not downgraded | The number of included studies was small (k = 2 < 10), but no significant publication bias was detected, as indicated by a symmetric funnel plot. |
| Overall quality of evidence | Very Low | Evidence from randomized controlled trials initially started at a high level but was downgraded by three levels due to concerns about risk of bias, significant heterogeneity, and imprecision in the pooled estimates. No further downgrading was applied for indirectness or publication bias. Therefore, the overall certainty of the evidence was rated as **very low** according to the GRADE framework. |

# **References**

1. Yang Y. An Empirical Study on the Influence of Tai Chi Exercise on College Students’ Physical Health Test Indicators. *Sci Technol Station Sport Goods* (2014)46–47.

2. Zhu X. The Effect of Tai Chi on Cardiopulmonary Function in College Students. *Sci Technol Vis* (2015)168–169. doi: 10.19694/j.cnki.issn2095-2457.2015.13.119

3. Li M, Fang Q, Li J, Zheng X, Tao J, Yan X, Lin Q, Lan X, Chen B, Zheng G. The effect of Chinese traditional exercise-Baduanjin on physical and psychological well-being of college students: a randomized controlled trial. *PloS one* (2015) 10:e0130544.

4. Zheng G, Lan X, Li M, Ling K, Lin H, Chen L, Tao J, Li J, Zheng X, Chen B. Effectiveness of tai chi on physical and psychological health of college students: results of a randomized controlled trial. *PLOS One* (2015) 10:e0132605. doi: 10.1371/journal.pone.0132605

5. Wu J, Hou P. Influence of 24-Form Tai Chi on Balance Function in College Students. *Contemp Sports Technol* (2015) 5:178–179. doi: 10.16655/j.cnki.2095-2813.2015.24.178

6. Wei Q, Zhao Q, Wu Y. Study of Yi Jin Jing on Mental health and Body Constitution in College Students. *Shandong J Tradit Chin Med* (2017) 36:654–56.

7. Yuan M, Xue H, Huang Y. The influence of 20 weeks Taijiquan exercise on the physical function of college students. *J Mil Sports* (2017) 36:85–88.

8. Lai Q, Jing C, Chen C, Jiao R, Pan H. Research on The Baduanjin on Health Promotion of Female College Students under the Background of Healthy China. *Fujian Sports Sci Technol* (2018) 37:44–6.

9. Chang Z, Zhang S. Positive Effects of Chen-Style Tai Chi (18-Form Essence) on College Students’ Cardiopulmonary Function. *Tradit Sports* (2020)73–74.

10. Jiao X, Ji H, Chen J. Effects of traditional Wuqinxi on physical fitness and mental health of female college students. *Chin JSch HEALTH* (2021)1323–1327.

11. Wang L. Effects of body-building exercise and tai chi on body composition, physical function and psychological indicators of female college students. *J Lanzhou Univ Arts Sci (Nat Sci)* (2021) 35:88–93.

12. Ye Y, Zhao F, Sun S, Xiong J, Zheng G. The effect of baduanjin exercise on health-related physical fitness of college students: a randomized controlled trial. *Front Public Health* (2022) 10:965544. doi: 10.3389/fpubh.2022.965544

13. Liu Z, Zhang J. On the Effect of Baduanjin Combined with Yijinjing Exercise on the Physical Health of College Students. *Wushu Stud* (2022) 7:80–83, 103. doi: 10.13293/j.cnki.wskx.009572

14. Zhang Y, Jiang X. The effect of baduanjin exercise on the physical and mental health of college students: a randomized controlled trial. *Medicine (Baltimore)* (2023) 102:e34897. doi: 10.1097/MD.0000000000034897

15. Niu Y, Buranarugsa R, Kuhirunyaratn P. Comparing the effects of Bafa Wubu tai Chi and Traditional He-style tai Chi exercises on physical health risk factors in overweight male college students: a randomized controlled trial. *International journal of environmental research and public health* (2023) 20:6323. doi: 10.3390/ijerph20146323

16. Wang Z, Zhang Z, Wu Y. The effects of baduanjin exercise on the psychological condition and heart rate variability of sports-disadvantaged college students: a randomised trial. *J Health Popul Nutr* (2024) 43:203. doi: 10.1186/s41043-024-00691-4

17. Niu Y, Buranarugsa R, Kuhirunyaratn P. Effects of bafa wubu and He-style tai chi exercise training on physical fitness of overweight male university students: a randomized controlled trial. *PLOS One* (2024) 19:e0297117. doi: 10.1371/journal.pone.0297117

18. Liu N, Hu Lingming, Zhang Xiaohan, LuYanyan, Chen Xiongbo. A randomized controlled trial on effects of Baduanjin and brisk walking on sleep quality in female college students. *Chin Ment HEALTH J* (2025) 39:691–697.
